# A global phylogeny of butterflies reveals their evolutionary history, ancestral hosts and biogeographic origins

**DOI:** 10.1038/s41559-023-02041-9

**Published:** 2023-05-15

**Authors:** Akito Y. Kawahara, Caroline Storer, Ana Paula S. Carvalho, David M. Plotkin, Fabien L. Condamine, Mariana P. Braga, Emily A. Ellis, Ryan A. St Laurent, Xuankun Li, Vijay Barve, Liming Cai, Chandra Earl, Paul B. Frandsen, Hannah L. Owens, Wendy A. Valencia-Montoya, Kwaku Aduse-Poku, Emmanuel F. A. Toussaint, Kelly M. Dexter, Tenzing Doleck, Amanda Markee, Rebeccah Messcher, Y-Lan Nguyen, Jade Aster T. Badon, Hugo A. Benítez, Michael F. Braby, Perry A. C. Buenavente, Wei-Ping Chan, Steve C. Collins, Richard A. Rabideau Childers, Even Dankowicz, Rod Eastwood, Zdenek F. Fric, Riley J. Gott, Jason P. W. Hall, Winnie Hallwachs, Nate B. Hardy, Rachel L. Hawkins Sipe, Alan Heath, Jomar D. Hinolan, Nicholas T. Homziak, Yu-Feng Hsu, Yutaka Inayoshi, Micael G. A. Itliong, Daniel H. Janzen, Ian J. Kitching, Krushnamegh Kunte, Gerardo Lamas, Michael J. Landis, Elise A. Larsen, Torben B. Larsen, Jing V. Leong, Vladimir Lukhtanov, Crystal A. Maier, Jose I. Martinez, Dino J. Martins, Kiyoshi Maruyama, Sarah C. Maunsell, Nicolás Oliveira Mega, Alexander Monastyrskii, Ana B. B. Morais, Chris J. Müller, Mark Arcebal K. Naive, Gregory Nielsen, Pablo Sebastián Padrón, Djunijanti Peggie, Helena Piccoli Romanowski, Szabolcs Sáfián, Motoki Saito, Stefan Schröder, Vaughn Shirey, Doug Soltis, Pamela Soltis, Andrei Sourakov, Gerard Talavera, Roger Vila, Petr Vlasanek, Houshuai Wang, Andrew D. Warren, Keith R. Willmott, Masaya Yago, Walter Jetz, Marta A. Jarzyna, Jesse W. Breinholt, Marianne Espeland, Leslie Ries, Robert P. Guralnick, Naomi E. Pierce, David J. Lohman

**Affiliations:** 1 McGuire Center for Lepidoptera and Biodiversity, Florida Museum of Natural History, University of Florida, Gainesville, FL USA; 2 Entomology and Nematology Department, University of Florida, Gainesville, FL USA; 3 Department of Biology, University of Florida, Gainesville, FL USA; 4 CNRS, Institut des Sciences de l’Evolution de Montpellier (Université de Montpellier), Montpellier, France; 5 Department of Ecology, Swedish University of Agricultural Sciences, Uppsala, Sweden; 6 Department of Biology, Washington University in St. Louis, St. Louis, MO USA; 7 Department of Entomology, National Museum of Natural History, Smithsonian Institution, Washington, DC USA; 8 Center for Biodiversity Research, Department of Biological Sciences, University of Memphis, Memphis, TN USA; 9 Department of Organismic and Evolutionary Biology and Museum of Comparative Zoology, Harvard University, Cambridge, MA USA; 10 Department of Botany and Plant Sciences, University of California, Riverside, Riverside, CA USA; 11 Florida Museum of Natural History, University of Florida, Gainesville, FL USA; 12 Department of Plant and Wildlife Sciences, Brigham Young University, Provo, UT USA; 13 Center for Global Mountain Biodiversity, Globe Institute, University of Copenhagen, Copenhagen, Denmark; 14 Center for Macroecology, Evolution, and Climate, GLOBE Institute, University of Copenhagen, Copenhagen, Denmark; 15 Biology Department, City College of New York, City University of New York, New York, NY USA; 16 Department of Life and Earth Sciences, Perimeter College, Georgia State University, Decatur, GA USA; 17 Department of Entomology, Natural History Museum of Geneva, Geneva, Switzerland; 18 PhD Program in Biology, Graduate Center, City University of New York, New York, NY USA; 19 Animal Biology Division, Institute of Biological Sciences, University of the Philippines Los Baños, Laguna, Philippines; 20 Laboratorio de Ecología y Morfometría Evolutiva, Centro de Investigación de Estudios Avanzados del Maule, Universidad Católica del Maule, Talca, Chile; 21 Millennium Institute Biodiversity of Antarctic and Subantarctic Ecosystems (BASE), Santiago, Chile; 22 Division of Ecology and Evolution, Research School of Biology, The Australian National University, Acton, Canberra, Australian Capital Territory Australia; 23 Australian National Insect Collection, Canberra, Australian Capital Territory Australia; 24 Entomology Section, National Museum of Natural History, Manila, Philippines; 25 African Butterfly Research Institute, Nairobi, Kenya; 26 Biology Centre CAS, České Budějovice, Czech Republic; 27 Department of Biology, University of Pennsylvania, Philadelphia, PA USA; 28 Department of Entomology and Plant Pathology, Auburn University, Auburn, AL USA; 29 Iziko South African Museum, Cape Town, South Africa; 30 Botany and National Herbarium Division, National Museum of the Philippines, Manila, Philippines; 31 College of Life Science, National Taiwan Normal University, Taipei, Taiwan; 32 Chiang Mai, Thailand; 33 Natural History Museum, London, UK; 34 National Centre for Biological Sciences, Tata Institute of Fundamental Research, Bengaluru, India; 35 Museo de Historia Natural, Universidad Nacional Mayor de San Marcos, Lima, Peru; 36 Department of Biology, Georgetown University, Washington, DC USA; 37 Faculty of Science, Department of Zoology, University of South Bohemia, České Budějovice, Czech Republic; 38 Department of Karyosystematics, Zoological Institute of Russian Academy of Sciences, St. Petersburg, Russia; 39 Turkana Basin Institute, Stony Brook University, Stony Brook, NY USA; 40 Hachiôji, Japan; 41 Departamento de Zoologia, Universidade Federal do Rio Grande do Sul, Porto Alegre, Brazil; 42 Vietnam Programme, Fauna & Flora International, Hanoi, Vietnam; 43 Vietnam National Museum of Nature, Vietnam Academy of Science and Technology, Hanoi, Vietnam; 44 Centro de Ciências Naturais e Exatas, Pós-Graduação em Biodiversidade Animal, Universidade Federal de Santa Maria, Santa Maria, Brazil; 45 Australian Museum, Sydney, New South Wales Australia; 46 Center for Integrative Conservation, Xishuangbanna Tropical Botanical Garden, Chinese Academy of Sciences, Mengla, China; 47 University of Chinese Academy of Sciences, Beijing, China; 48 College of Arts and Sciences, Jose Rizal Memorial State University, Tampilisan, Philippines; 49 Aquapro, Villavicencio, Colombia; 50 Entomology Laboratory, Museo de Zoología, Universidad del Azuay, Cuenca, Ecuador; 51 Research Center for Biosystematics and Evolution, National Research and Innovation Agency (BRIN), Cibinong-Bogor, Indonesia; 52 Institute of Silviculture and Forest Protection, University of West Hungary, Sopron, Hungary; 53 The Research Institute of Evolutionary Biology (Insect Study Division), Setagaya, Japan; 54 Köln, Germany; 55 Institut Botànic de Barcelona (IBB, CSIC-Ajuntament de Barcelona), Barcelona, Spain; 56 Institut de Biologia Evolutiva (CSIC-Univ. Pompeu Fabra), Barcelona, Spain; 57 T.G. Masaryk Water Research Institute, Prague, Czech Republic; 58 Department of Entomology, College of Plant Protection, South China Agricultural University, Guangzhou, China; 59 The University Museum, The University of Tokyo, Tokyo, Japan; 60 Department of Ecology & Evolutionary Biology, Yale University, New Haven, CT USA; 61 Center for Biodiversity and Global Change, Yale University, New Haven, CT, USA; 62 Translational Data Analytics Institute, The Ohio State University, Columbus, OH USA; 63 Department of Evolution, Ecology and Organismal Biology, The Ohio State University, Columbus, OH USA; 64 RAPiD Genomics, Gainesville, FL USA; 65 Leibniz Institute for the Analysis of Biodiversity Change, Zoological Research Museum Alexander Koenig, Bonn, Germany

**Keywords:** Phylogenetics, Entomology

## Abstract

Butterflies are a diverse and charismatic insect group that are thought to have evolved with plants and dispersed throughout the world in response to key geological events. However, these hypotheses have not been extensively tested because a comprehensive phylogenetic framework and datasets for butterfly larval hosts and global distributions are lacking. We sequenced 391 genes from nearly 2,300 butterfly species, sampled from 90 countries and 28 specimen collections, to reconstruct a new phylogenomic tree of butterflies representing 92% of all genera. Our phylogeny has strong support for nearly all nodes and demonstrates that at least 36 butterfly tribes require reclassification. Divergence time analyses imply an origin ~100 million years ago for butterflies and indicate that all but one family were present before the K/Pg extinction event. We aggregated larval host datasets and global distribution records and found that butterflies are likely to have first fed on Fabaceae and originated in what is now the Americas. Soon after the Cretaceous Thermal Maximum, butterflies crossed Beringia and diversified in the Palaeotropics. Our results also reveal that most butterfly species are specialists that feed on only one larval host plant family. However, generalist butterflies that consume two or more plant families usually feed on closely related plants.

## Abstract (web summary)

A new phylogenomic tree for butterflies is constructed using 391 genes from 2,300 species, representing 92% of genera. It suggests that butterflies originated around 100 million years ago in what is now the Americas and originally fed on Fabaceae.

## Main

Butterflies have long captivated naturalists, scientists and the public, and they have played a central part in studies of speciation, community ecology, plant–insect interactions, mimicry, genetics and conservation. Despite being the most intensely studied insect group, the evolutionary history and drivers of butterfly diversification remain poorly understood^1,2^. Butterflies are thought to have diversified in relation to multiple abiotic and biotic factors, including adaptations to novel climates and species interactions, with caterpillar–host interactions and geographic history playing a major role^3^. However, these hypotheses have not been tested because a robust phylogenetic framework at the taxonomic scale that would be needed to examine their evolution has not been available. Furthermore, host plant and distribution data have largely been scattered across literature, museum collections, and local databases, limiting our ability to conduct broad, comparative macroevolutionary studies.

**Fig. 1:**
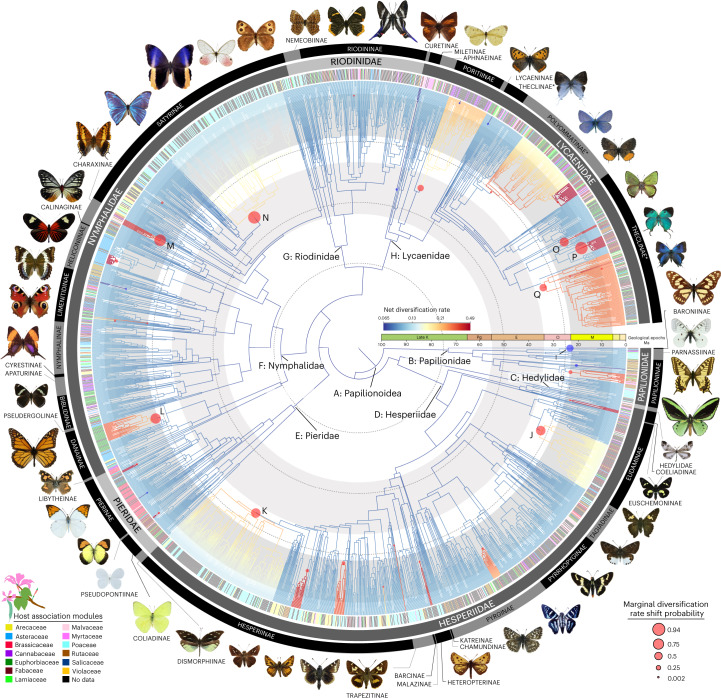
Evolutionary relationships and diversification patterns of butterflies. Time-calibrated tree of 2,244 butterfly species based on 391 loci and 150 amino acid partitions. Branches show distinct changes in diversification (circles) as estimated by clade-specific models. Letters at nodes refer to clades with significant rate shifts (see section 6 of Supplementary Results). Coloured lines in the outer ring beside tips indicate association with one of the 13 host modules (see section 17 of Extended Online Methods). Black lines in the host association ring indicate species without data, and asterisks denote non-monophyletic subfamilies. Supplementary Fig. 1 shows this tree with visible species names and ages for all nodes.

We sequenced 391 genes from nearly 2,300 butterfly species to reconstruct a new phylogenomic tree of butterflies representing 92% of all genera (Fig. 1 and Supplementary Fig. 1), assembled a comprehensive host association dataset and aggregated global distribution records. Using our tree, we inferred the evolutionary timing, patterns of host use, and biogeographic history of butterflies. We addressed three long-standing questions related to butterfly evolution: (1) did butterflies originate in the northern (Laurasia) or southern (Gondwana) hemisphere^4^; (2) what plants did the ancestor of butterflies feed on^5^; and (3) are host repertoires (that is, diets) of butterfly species and clades constrained by host phylogeny^6,7^?

## Results and discussion

To elucidate patterns of global butterfly diversification in space and time, we used targeted exon capture^8^ to assemble a dataset of 391 gene regions (161,166 nucleotides and 53,722 amino acids) from 2,244 butterfly species (Supplementary Table 1). The majority (1,914 specimens) of butterflies sampled were newly sequenced for this study, representing all families, subfamilies and tribes, and 92% of recognized genera, from 90 countries. These were obtained from 28 specimen collections across the world (see section 2 of the Extended Online Methods). Phylogenomic trees were inferred with nucleotides or translated amino acids with nine different subsets and partitioning schemes. Our trees were highly congruent, with strong support for the monophyly of all families and nearly all subfamilies with branch support metrics (SH-aLRT, ultrafast bootstrap) and multispecies coalescent species tree analyses (Supplementary Table 2). We also conducted four-cluster likelihood mapping to identify potentially conflicting signals in our datasets (Supplementary Table 3). Our results strongly support the need for revision of the classification of at least 36 butterfly tribes (27% of total) as currently circumscribed (Supplementary Table 2).

We conducted 24 dating analyses using different fossil and secondary calibration schemes along with sensitivity analyses to assess the impact of analytical and sampling bias. Across analyses, our results revealed largely congruent timing of butterfly divergence events (Supplementary Table 4). Butterflies originated from nocturnal, herbivorous moth ancestors around 101.4 million years ago (Ma) (102.5–100.0 Ma), providing evidence for a mid-Cretaceous origin of butterflies^2,9^.

**Fig. 2:**
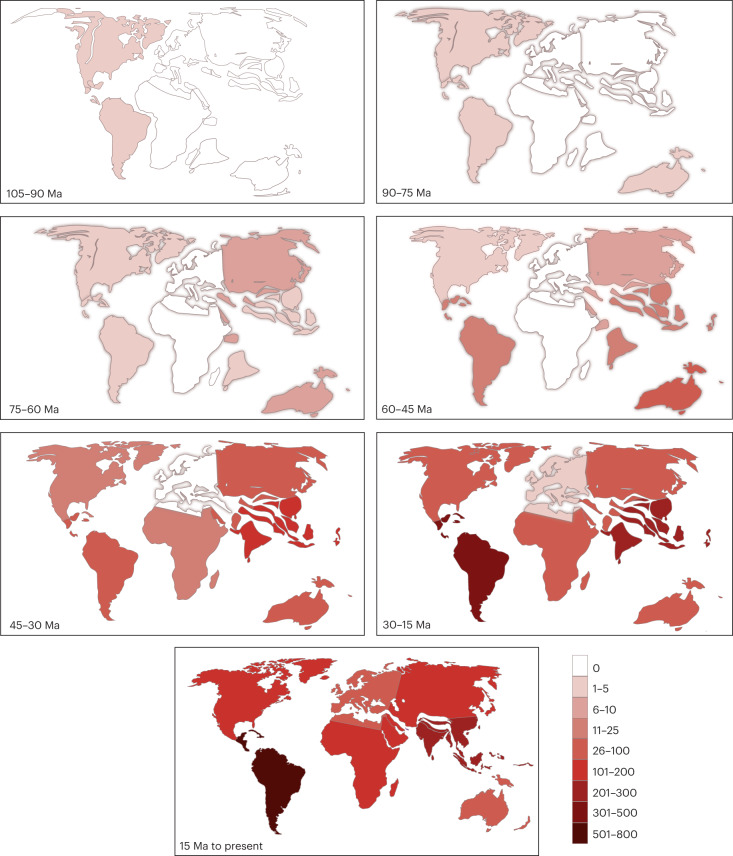
Distribution of butterflies over time. Bioregion shading indicates the number of butterfly lineages that were associated with that bioregion during that time period, as determined by BioGeoBEARS ancestral state reconstruction. Each map corresponds to a 15-Ma interval of butterfly evolution. Results are based on data from this study.

**Fig. 3:**
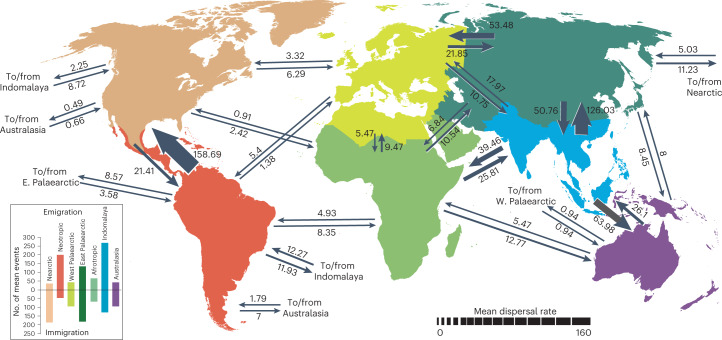
Relative mean dispersal rates of butterflies between bioregions. Numbers beside each arrow are average rates from 1,000 simulations using biogeographic stochastic mapping in BioGeoBEARS. These numbers were divided by 100 for ease of comparison (raw values can be found in Supplementary Data 5). E., Eastern; W., Western.

To determine the geographic origin of butterflies, we used our dated tree (Fig. 1) to conduct a global biogeographic analysis with 15,764 newly aggregated country-level distribution records (Supplementary Table 5). Modelling with three different area categorizations, models of range evolution and parameters (adjacency matrices, time slices, etc.) consistently recovered butterflies as originating in the Americas, in what is present-day western North America or Central America (Fig. 2 and Supplementary Tables 6 and 7). All extant butterfly families excluding the Neotropical Hedylidae diversified ~10–30 Ma after the Cretaceous Thermal Maximum, ~90 Ma, when the global climate cooled by nearly 5 °C (ref. ^10^) (Figs. 1 and 2). During the Cretaceous, butterflies dispersed out of the Neotropics at a much higher rate than that of any other dispersal route (Supplementary Fig. 2). As new butterfly lineages became established in other bioregions, interbioregion dispersals became more frequent, particularly out of the present-day Indo-Australian Archipelago (Supplementary Figs. 3 and 4). Beginning around 60 Ma, the Neotropics served as an important bioregion with high in situ butterfly speciation (Supplementary Fig. 5), and many lineages dispersed out of this region to other areas (Supplementary Fig. 6). The relative rate of dispersal out of the Neotropics remained high during the early Cenozoic, although not as much as it was during the Cretaceous (Supplementary Figs. 2 and 3). Over the course of evolution, butterfly speciation was substantially higher in the tropics than in temperate zones (Supplementary Data 1). More dispersal events originated in the tropics (Supplementary Fig. 6), as evidenced by relative mean out-of-tropics dispersal rates from the temperate Eastern Palaearctic, and from the Neotropics to the Nearctic (Fig. 3). This pattern differs from that seen in mammals, which are thought to have dispersed primarily in the opposite direction during the Pliocene^11–13^. Our estimates of within-area dispersal rates (Supplementary Figs. 7 and 8) indicate that some butterflies, including swallowtails (Papilionidae), contradicted the general trend and dispersed into the Neotropics at high rates, corroborating previous findings^14^. Most dispersal events between the Neotropics and the Nearctic took place after the Eocene/Oligocene boundary, ~33.9 Ma (Supplementary Fig. 4), congruent with a previous biogeographic study^15^. Two lineages dispersed from the Eastern Palaearctic around 17 Ma, and these appear to be the first colonizers of Europe: ancestors of the Nymphalini subclade including *Aglais*, *Nymphalis* and *Polygonia*, and a clade of chequered skippers (Carcharodini; Supplementary Table 7). Butterflies were present on what are now all modern continental landmasses by the late Eocene (Supplementary Table 8).

To understand the evolution of larval host plant use, we compiled 31,456 butterfly host records from 186 books, published papers, and public and private databases (Supplementary Table 9). We found that butterfly origin and diversification lagged far behind the origin of angiosperms^16–18^, corroborating previous studies^8,19^. We used a recently developed network approach to create host plant modules to infer the associations of butterflies and plants^6,20^. Butterfly host plants include more than 80 orders and ~300 families^21^, rendering standard ancestral state reconstruction intractable. Our analyses provide support for Fabaceae as the larval host plant of the most recent common ancestor of butterflies (Supplementary Tables 10 and 11 and Supplementary Fig. 9), a widely accepted hypothesis^5^ that has lacked empirical support. The crown age of the most recent common ancestor of Fabaceae is thought to be ~98 Ma (refs. ^16,18^), largely coincident with the origin of butterflies.

Although most butterflies in our dataset are herbivores as larvae, a small number also feed on detritus, lichens or other insects (Supplementary Table 9). The oldest associations in the entirely entomophagous Miletinae (Lycaenidae) appear to originate by 58.4 Ma (58.9–57.1 Ma), a date that largely corresponds with an earlier estimation of the origin of this subfamily^22^ (Supplementary Tables 4 and 12). Lycaenidae, with caterpillars that are ancestrally symbiotic with ants^8,23^, date back to 64.5 Ma (65.4–63.7 Ma) (Supplementary Fig. 10), long after the origin of ants (139–158 Ma)^24^. Together with plants, ants appear to have provided a template for diversification of Lycaenidae and some members of its sister clade, Riodinidae. Our host database provides an important resource for future studies on butterfly feeding patterns.

We examined host plant specificity on the butterfly phylogeny (Fig. 1) and found that more than two-thirds of extant butterfly species feed on a single plant family (67.7%), whereas less than a third (32.3%) are generalists feeding on two or more (Supplementary Table 13), a pattern largely in agreement with ecological studies^25^. Butterflies feeding on grass and legumes (Poaceae and Fabaceae) are often host specific; the majority do not feed on plants from other families (Supplementary Table 9). These two plant families are geographically widespread and abundant in almost every ecosystem^26,27^, and most grasses and legumes lack potent defensive chemicals that restrict insect feeding^28^. These plant traits may have allowed butterflies to remain associated with these plant families for millions of years. We also found that 94.2% of generalists feed on plant families that are significantly closely related compared with a randomly sampled null distribution, suggesting that ‘generalists’, although capable of feeding on different host families, still consume closely related plants. This finding supports the pattern proposed by Ehrlich and Raven^29^ in which related butterflies feed on related plants.

Our study provides a robust baseline for future studies of this model insect lineage. The consistency of results obtained using different approaches for each of our analyses suggests that our conclusions are robust. Our data support the hypothesis that butterflies originated in the Americas in the late Cretaceous, 100 million years after the origin of angiosperms, and that they first fed on legumes. Butterflies dispersed from the Americas to the Eastern Palaearctic across Beringia ~75 Ma before diversifying in the Palaeotropics. Although our analyses point to a Nearctic origin, evidence for a North American versus a Central American origin is not strong, and we therefore tentatively conclude that a Laurasian origin is likely. Larval host plants played an important part in the evolution of butterflies, and some groups became host specific whereas others retained a wide host breadth. The molecular, host plant and geographic data provided here serve as a baseline for future comparative analyses of butterflies.

## Methods

### Taxon sampling and sequence acquisition

A total of 2,248 butterfly specimens representing 2,244 species in 1,644 genera were included for the molecular component of this study, along with ten outgroups from other lepidopteran superfamilies (Supplementary Table 1). The ingroup included genera from all families, subfamilies and tribes of butterflies according to the current classification. We aimed to include at least one species from every valid genus and sequenced the type species of each genus whenever possible. We obtained 92% of all described valid butterfly genera when the initial dataset was assembled (July 2019).

We obtained marker loci for phylogenetic analysis by (1) anchored hybrid enrichment exon capture of DNA extracts and subsequent Illumina sequencing^30^ or (2) bioinformatically removing these sequences from published genomes and transcriptomes. We used the BUTTERFLY1.0 probe set^8^ and selected a 391-locus subset that was captured reliably in at least 60% of samples. We chose this approach because it has been proven to resolve relationships of many different butterfly groups^31–34^. The BUTTERFLY1.0 probe set includes 13 genes (12 nuclear genes and the COI mitochondrial gene) that have been widely used in butterfly phylogenetics^9,35^, also termed ‘legacy genes’^36^, and additional protein-coding genes that may be used to address broad questions pertaining to butterfly biology, such as vision, host use and olfaction^8^.

Specimens were collected in 90 countries over a 70-year period by over 300 people and deposited in one of the 28 specimen collections from which we obtained tissue samples (Supplementary Table 1). We successfully captured and sequenced DNA from decades-old museum specimens^37^, which enabled us to include taxa that are rare or live in areas where collecting fresh material is difficult. The oldest sample was a pinned specimen collected on 22 April 1946: *Dira clytus* (Nymphalidae) (LEP79391). Images of 460 representative voucher specimens are shown in Supplementary Data 2, and specimen repositories are listed in Supplementary Table 1. All voucher specimens, at minimum, had their wings and genitalia retained for identification and future research.

We obtained sequence data from 343 published genomes and transcriptomes. Ten of these were outgroups representing nine moth families that are closely related to butterflies according to published studies on lepidopteran phylogeny^9,38–42^.

We extracted DNA from 1,915 specimens that were (1) stored in ethanol and frozen; (2) dried and stored in glassine envelopes under ambient conditions (papered); or (3) dried, spread and pinned in a museum collection. Locus assembly and sequence clean-up followed the pipeline of Breinholt et al.^42^. Published sequences comprised (1) genome assemblies, (2) genomic reads, and (3) paired or (4) single-end transcriptomes. Three sequence datasets were created for this study: a nucleotide dataset with all codon positions (nt123); a nucleotide dataset that excludes all synonymous changes (degen), created using the Perl script Degen1 v.1.4 (refs. ^43,44^); and an amino acid (aa) dataset translated from the nt123 dataset (Supplementary Data 3).

### Phylogenetic analysis and dating

Maximum likelihood (ML) tree inference was conducted on all three datasets (nt123, degen and aa) in IQ-TREE 2.0 (ref. ^45^); parameter settings for each analysis can be found in Supplementary Table 14. Branch support was calculated with 1,000 ultrafast bootstrap replicates (UFBS; ‘-B 1000’ command)^46,47^ and Shimodaira–Hasegawa approximate likelihood ratio tests (SH-aLRT; ‘-alrt 1000’ command)^48^. Quartet sampling was performed on the degen359 and aa154 trees with the highest likelihood score. Four-cluster likelihood mapping analyses^49^ were performed on the degen and aa datasets to assess the placement of particular butterfly clades that have been the subject of previous phylogenetic studies. We applied this approach in addition to standard branch support metrics, because the latter can be subject to inflated estimates^49^.

We obtained divergence time estimates using a penalized-likelihood based approach implemented in treePL^50^. We implemented three different methods for calibrating trees and assessed similarities among results. Method 1 involved dating with secondary calibrations only. We used the 95% credibility intervals of Lepidoptera ages from Fig. S12 of Kawahara et al.^38^ to assign minimum and maximum ages to 27 ingroup and six outgroup nodes in our tree. Method 2 involved dating with fossils and one secondary root calibration. In this approach, we followed the guidelines of Parham et al.^51^ by calibrating nodes with 11 butterfly fossils that could be assigned to the geological age of a butterfly lineage with confidence as verified by de Jong^52^. None of the outgroup nodes could be calibrated because reliable fossils associated with our non-butterfly Lepidoptera were too young to influence deeper node ages representing multisuperfamily clades. Consequently, preliminary treePL analyses yielded highly dubious age estimates for deep nodes on the tree, hundreds of millions of years older than expected based on the literature. We therefore added a single secondary calibration to the root of the tree. Although combining secondary and fossil calibrations in a single analysis can create redundancy that negatively affects the resulting age estimates^53^, the limited fossil record of Lepidoptera made it a necessity to obtain comparable results derived primarily from fossils. We ran two versions of this method, each with a different root calibration. Method 2A used a maximum-age estimate of 139.4 Ma, based on the angiosperm age estimate of Smith and Brown^17^. Method 2B used a more conservative maximum-age estimate of 251 Ma, based on the older end of the credibility interval for the age of angiosperms in Foster et al.^54^. Both calibrations were used under the assumption that butterflies diverged from their moth ancestors after their most frequently used host plants, angiosperms, were already present^55,56^. Method 3 involved secondary calibrations and six fossils. In this approach, we combined the 33 secondary calibrations from Method 1 with six fossil calibrations, including some of the fossils used in Method 2. Fossils previously used to calibrate trees of Kawahara et al.^38^ were excluded from this analysis to avoid circularity and redundancy with secondary calibrations. Whenever possible, redundant fossil calibrations from Method 2 were replaced with calibrations from unrelated fossils that could be associated with a different node in the same clade.

### Diversification rate analyses

We performed a Bayesian analysis of macroevolutionary mixtures using the program BAMM v.1.10.4 (ref. ^57^) to detect shifts in diversification rates between clades. Reversible-jump Markov chain Monte Carlo was run for 50 million generations and sampled every 50,000 generations. Priors were estimated with the R package BAMMtools v.2.1.6 (ref. ^58^) using the command ‘setBAMMpriors’. The tree was trimmed in Mesquite v.3.6 (ref. ^59^) to remove all outgroups. Six analyses were performed using different priors for expected numbers of shifts (5, 10, 20, 30, 40 and 50 shifts).

We conducted a series of analyses in HiSSE (Hidden State Speciation and Extinction) and a BiSSE-like (Binary State Speciation and Extinction) implementation of HiSSE^60^ in the R package hisse^61^ to evaluate whether there is a correlation between butterfly and plant diversification. We pruned outgroups from the aa154 dated tree (Strategy A) and compared 20 HiSSE models and BiSSE-like implementations of HiSSE. The BiSSE equivalent of HiSSE tests whether there are different diversification rates associated with the two host plant use states. Other models were built in the HiSSE framework to test alternative combinations of the presence or absence of hidden state and host plant use associations while also considering different transition rate matrices, net turnover rates, τi (speciation plus extinction: λi + μi) and extinction fractions, εi (extinction divided by speciation: μi/λi) (Supplementary Table 15). We tested whether diversification rates were linked to feeding (A) as a larval specialist or generalist (Supplementary Table 16); (B) on Poales (Supplementary Table 17) in Papilionoidea, Hesperiidae and Nymphalidae; (C) on Fabales (Supplementary Table 18) in Papilionoidea and Nymphalidae; (D) on Brassicales (Supplementary Table 19) in Papilionoidea and Pieridae; (E) on Fagales (Supplementary Table 20); (F) on the Poaceae module (Supplementary Table 21); (G) on the Fabaceae module (Supplementary Table 22); and (H) on Fabaceae in Eudaminae (Supplementary Tables 22 and 23). We compared these different models of HiSSE and BiSSE-like implementations to account for hidden states to alleviate concerns that SSE models can lead to a high incidence of false positive results^62^.

Fraction files of clade-based taxonomic diversity estimates were created for all HiSSE runs to account for taxonomic sampling bias (Supplementary Table 24). We set the total number of extant butterfly species as 19,500, which is an ~8% increase compared with the butterfly species richness estimate of van Nieukerken et al.^63^. We added this diversity correction based on many recent new butterfly species descriptions (for example, by Cong et al.^64^) and morphospecies that we are aware of that have not yet been formally described. We estimated the total number of generalist and specialist species by calculating the percentage of generalists and specialists in our dataset at the family level. We standardized the proportion of species richness in that family compared to all butterflies, based on diversity estimates of van Nieukerken et al.^63^. For example, 78.61% of all sampled Hesperiidae that had host data were specialists, and Hesperiidae comprise 21.91% of all butterfly species richness; thus, we estimated Hesperiidae specialists as 19,500 × 0.2191 × 0.7861 = 3,359 species. Applying these calculations for all families yielded totals of 12,969 specialist species and 6,531 generalist species (Supplementary Table 25); these numbers were used to estimate fractions of generalists and specialists in our dataset.

Calculating the fraction of species sampled within each host plant module proved more challenging. To estimate the true butterfly species richness for each module, we used unpublished estimates of species richness for all butterfly genera by G.L. and assumed that if a species was known to belong to a module, so would some of its congeners. These calculations were revised because some genera had large host ranges with species assigned to multiple modules. For example, the three species of *Vanessa* with host records in our dataset were assigned to three different modules. As there is an estimated total of 24 *Vanessa* species, we calculated that approximately 24/3 = 8 *Vanessa* species belonged in each of those modules. Calculations for all genera in all modules, and the resulting estimates of module totals and fractions sampled, are provided in Supplementary Table 26.

### Biogeography

To reconstruct the biogeographic history of butterflies, we aggregated global distribution data from multiple sources to create a butterfly checklist for each country. Data sources included: (1) the Lepidoptera and Other Life Forms Database (http://ftp.funet.fi/index/Tree_of_life/insecta/lepidoptera); (2) WikiSpecies (https://species.wikimedia.org); and (3) the type locality of each species or subspecies in our list of valid butterfly names, which was obtained from 1, above. This initial global checklist was vetted using published country checklists and the ButterflyNet Trait Database^65^. Trait data from ca. 100 comprehensive and country-specific field guides have been entered into this database, allowing us to generate species lists to cross-validate checklists assembled^66^.

We designated 14 biogeographic regions across the globe (Supplementary Fig. 11 and Supplementary Table 27), determined which of these regions were occupied by each species in our tree and developed a 14-state character matrix. Six countries (Canada, China, Indonesia, Mexico, Russia, US) spanned two or three bioregions, which required manual evaluation of whether species in these countries were found in one or more of the adjoining bioregions. US and Canadian species were assigned to East and/or West Nearctic bioregions based on the palaeogeographic history of North America (that is, whether the species were east or west of the continental divide) with reference to locality records from Butterflies and Moths of North America (https://www.butterfliesandmoths.org). Russian species were assigned to Eastern and/or Western Palaearctic bioregions based on locality records assembled by the Lepidoptera and Other Life Forms Database^67^. Some countries did not have complete distribution lists and were thus evaluated manually by coauthors. Chinese species were assigned to Eastern Palaearctic and Oriental bioregions by H.W. Indonesian species were assigned to Oriental, Wallacean and Australian bioregions by D.J.L. and D.P. Mexican species were assigned to East Nearctic, West Nearctic and Central American bioregions by J.I.M.

The majority of butterfly species are distributed in fewer than five bioregions. Some species are more widespread, but we found that this was often due to recent anthropogenic introductions. Consequently, a final round of data cleaning was performed in which records of species found in at least five bioregions were manually verified and edited to accurately reflect true native species’ ranges. Cleaned bioregion and tropicality data were converted to character matrices to be used for subsequent distribution analyses (Supplementary Tables 28 and 29).

We estimated the ancestral area of origin and geographic range evolution for butterflies using two approaches: the ML approach of the DECX model^68^ as implemented in the C++ version^69,70^ (https://github.com/champost/DECX); and the program BioGeoBEARS v.1.1.2 (ref. ^71^). DECX uses a time-calibrated tree, the modern distribution of each species for a set of geographic areas and a time-stratified geographic model that is represented by connectivity matrices for specified time intervals spanning the evolutionary history of clade of interest^72^.

We also ran BioGeoBEARS with seven and eight areas to estimate immigration and emigration rates (Supplementary Figs. 12 and 13 and Supplementary Table 27). BioGeoBEARS could not be run with 14 states owing to the complexity of our dataset (2,248 tree tips). The seven and eight bioregions largely corresponded to the biogeographic realms defined by Udvardy^73^. We implemented both the Dispersal Extinction Cladogenesis (DEC)^68,74^ and the Likelihood equivalent of the Dispersal-Vicariance approach (DIVALIKE)^75^ models and different adjacency matrices (Supplementary Data 4). Both approaches gave largely consistent results, regardless of the model and parameters used (Supplementary Tables 6 and 30).

We performed biogeographic stochastic mapping to examine in situ speciation, immigration and emigration between the seven bioregions in BioGeoBEARS. We followed Li et al.^76^ and ran 1,000 simulations with the DEC model, and calculated relative mean dispersal rates between all permutations of bioregions (Fig. 3 and Supplementary Data 5). These mean dispersal rates represent dispersal of butterfly lineages throughout the entire evolutionary history of Papilionoidea and thus cannot reveal changes in rates over time. To look at historical biogeography of butterflies during different epochs, rates along all possible interbioregion colonization rates were calculated at specific time intervals of 5 million years (Supplementary Table 31). These relative rates were averaged to represent relevant geological time periods (Supplementary Figs. 2–4).

### Larval host plant analyses

Larval host records were compiled from nine sources: (1) the Database of the World’s Lepidopteran Hostplants (HOSTS)^21^, which summarizes data from ~270 other sources; (2) the Lepidoptera and Other Life Forms Database (http://ftp.funet.fi/index/Tree_of_life/insecta/lepidoptera/); (3) 40 years of food plant rearing records from Costa Rica by D.H.J., W.H., and colleagues (http://janzen.sas.upenn.edu/); (4) the ButterflyNet Trait Database^65^, which includes host plant records from 109 butterfly field guides and other resources; (5) a comprehensive database for host records for all butterflies in Japan^77^; (6) a set of papers documenting the hosts of butterflies in India^78–84^; (7) a database of hosts and ant symbionts of larval Lycaenidae and Riodinidae compiled from 85 literature sources by N.E.P. and members of her laboratory; (8) a database of butterfly host records from Ecuador based on field observations and literature records compiled by K.R.W.; and (9) 88 papers from the primary literature or relevant websites (Supplementary Table 9 and Supplementary Data 6). Whenever possible, we retained the following information for each host record, if available: (1) the taxon and taxonomic authority of butterfly to the lowest available taxonomic level (family, subfamily, tribe, genus, species or subspecies); (2) the taxon and taxonomic authority of host to the lowest available taxonomic level (family, genus, species, subspecies or variety); (3) plant part eaten; (4) record certainty (novel plant accepted in captivity, oviposition record with no observation of herbivory, etc.); (5) geographic location of observation; and (6) relevant information on all non-plant hosts. The extensive data recorded in the host (food plant) database of D.H.J., W.H., and colleagues were simplified to retain the fields of butterfly genus and specific epithet, as well as plant family, genus and specific epithet, together with an indication of whether the plant was introduced to Costa Rica. This database contains many records of informal, non-ICZN-compliant names of butterfly cryptic species. Rather than discarding the large number of records that would not be compatible with any other data source, we regarded these as the nominal species (for example, *Battus polydamas* instead of *Battus polydamas*DHJ01). The number of records for each butterfly species × plant species interaction was recorded.

We examined relationships between individual butterfly species and host families that are consumed by their larvae. For these analyses, we chose the rank of plant family because it has been adopted as the standard taxonomic rank for examining host use evolution^6,85^. For each plant-feeding butterfly species in our tree, we quantified host plant richness and phylogenetic distance using six different metrics implemented in the R package *picante* v.1.8.2 (ref. ^86^). To calculate these metrics, we used the calibrated tree of seed plants from Smith and Brown^17^.

As the number of host groups in our dataset was too large for an ancestral state reconstruction (approximately 200 of the 300 known host plant families^21^ plus host insects), we first reduced the number of host groups by using a network analysis. The Beckett algorithm^87^, as implemented in the function ‘computeModules’ from the package bipartite^88^ in R v.3.6.2 (ref. ^89^), assigns plants and butterflies to modules and computes the modularity index, Q. By maximizing Q, the algorithm finds groups of butterflies and hosts that interact more with each other than with other taxa in the network. Thus, hosts that are assigned to the same module tend to be used by the same butterflies. We found 13 modules for butterfly host associations in our module analysis (Supplementary Tables 32 and 33). We then conducted three larval host ancestral state reconstruction analyses using stochastic character mapping with SIMMAP in phytools v.0.7-70 (refs. ^90,91^) using the ‘make.simmap’ command. We reconstructed the ancestral state of (A) generalist versus specialist feeding (two states, Supplementary Data 7); (B) plant, lichen, Hemiptera or Hymenoptera as a food source (four states, Supplementary Data 8); and (C) plant module (13 states, Supplementary Data 9).

### Reporting summary

Further information on research design is available in the Nature Portfolio Reporting Summary linked to this article.

## Supplementary information

Supplementary Information Extended Online Methods, Supplementary Results and Figs. 1–26.

Reporting Summary

Supplementary Tables Supplementary Tables 1–52.

## Data Availability

All supplementary data archives are available on Figshare (10.6084/m9.figshare.21774899). Genomic data for all newly sequenced specimens in this study have been uploaded to GenBank as part of BioProject PRJNA714105. Individual BioSample accession numbers for each specimen are provided in Supplementary Table 1.

## Code availability

All new code developed to aid with the analyses in this study has been uploaded to GitHub and made publicly available. GitHub URLs for specific scripts are provided in the Methods and Extended Online Methods sections.
