# Supplementary material for: A global phylogeny of butterflies reveals their evolutionary history, ancestral hosts and biogeographic origins
Source: Nat Ecol Evol. 2023 May 15;7(6):903–13. doi: 10.1038/s41559-023-02041-9 (PMC10250192; doi:10.1038/s41559-023-02041-9)
Supplement: Supplementary file 1 — Extended Online Methods, Supplementary Results and Figs. 1–26. [file 41559_2023_2041_MOESM1_ESM.pdf]

nature ecology & evolution

Article https://doi.org/10.1038/s41559-023-02041-9

A global phylogeny of butterflies reveals their evolutionary history, ancestral hosts and biogeographic origins

In the format provided by the authors and unedited

Table of Contents

Contents	Page
Extended Online Methods	2
Supplementary Text	37
Supplementary Figs. S1-S26	63

Extended Online Methods

A Global Phylogeny of Butterflies Reveals Their Evolutionary History, Ancestral Hosts, and Biogeographic Origins

Contents:

1. Butterfly nomenclature
2. Taxon sampling, marker selection, and permits
3. DNA extraction
4. DNA quantification and sequencing
5. Sequence assembly and clean-up
6. Extraction of BUTTERFLY1.0 loci from genomes and transcriptomes
7. Dataset construction
8. Sequence quality check
9. Datasets, partitioning and model selection
10. Tree inference and branch support
11. Coalescent-based methods
12. Topology tests
13. Divergence time estimation
14. Diversification analyses
15. Larval host dataset assembly and curation
16. Hosts and phylogenetic distance
17. Ancestral state reconstruction
18. Model-based comparisons of speciation rate in butterflies and angiosperms
19. Coding and curation of distributional data
20. Model-based comparisons of biogeographic patterns and evolutionary rates

Materials and Methods

1. Butterfly nomenclature

We created a list of butterfly valid names derived from local and global catalogs[1,2] because butterfly taxonomy is in constant flux. This list was augmented by aggregating synonyms from multiple sources, including the *Lepidoptera and other life forms* database (http://ftp.funet.fi/index/Tree_of_life/insecta/lepidoptera/) and WikiSpecies (https://species.wikimedia.org/wiki/Main_Page). Our list was updated when taxonomic changes and newly described species were published (e.g., Cong et al.[3]). Taxotools software[4] was developed to standardize all butterfly taxonomy and associated data used throughout this project, including the phylogeny, larval host data, and geographic distributions.

2a. Taxon sampling and marker selection for molecular analysis

A total of 2,248 butterfly specimens representing 2,244 species in 1,644 genera was included for the molecular component of this study, along with 10 outgroups from other lepidopteran superfamilies (Table S1). The ingroup includes genera from all families, subfamilies, and tribes of butterflies according to the current classification. We aimed to include at least one species from every valid genus and sequenced the type species of each genus whenever possible. We had obtained 92% of all described, valid butterfly genera when the initial dataset was assembled (July, 2019).

We obtained marker loci used for phylogenetic analysis by: 1) Anchored Hybrid Enrichment (AHE) exon capture of DNA extracts and subsequent Illumina sequencing[5], or 2) bioinformatically removing these sequences from published genomes and transcriptomes. We used the BUTTERFLY1.0 probe set[6] and selected a 391-locus subset that was captured reliably in at least 60% of samples. These markers include 13 genes (12 nuclear and the COI mitochondrial gene) that have been widely used in butterfly phylogenetics[7], also termed "legacy genes"[8], and additional protein-coding genes that may be used to address broad questions pertaining to butterfly biology, such as vision, host use, and olfaction[6].

Specimens were collected in 90 countries over a 70-year period by over 300 people, preserved and deposited in one of the 28 specimen collections from which we obtained new tissue samples (Table S1). The oldest specimen was a pinned museum specimen collected on 22 April 1946: *Dira clytus* (Nymphalidae) (LEP79391). In some cases, we verified species

determinations using a specimen's COI sequence (DNA barcode) and compared it to the COI database of boldsystems.org[9] using BLAST[10]. Prior to extraction, digital photographs were taken with each specimen's associated collection data whenever possible (Data S2). For EtOH preserved specimens, we created wing vouchers following Cho et al.[11] and an Epson Perfection V600 flatbed photo scanner was used to scan vouchers at 600 dpi. For dry specimens, we used an iPhone 7 camera (12-megapixel rear-facing camera with quad-LED lights) to record a digital image of the specimen. Specimen voucher images of 460 representative specimens are shown in Data S2 and specimen repositories are listed in Table S1. All voucher specimens, at minimum, retained their wings and genitalia for identification and future research.

We obtained sequence data from 343 published genomes and transcriptomes (see Materials and Methods - *Extraction of BUTTERFLY1.0 loci from genomes and transcriptomes*, below). Ten of these were outgroups representing nine moth families that are closely related to butterflies according to published studies on lepidopteran phylogeny[12–16].

2b. Collection permits and documentation

The majority of specimens that were used for the genetic portion of this study were obtained from legs and abdomens of museum specimens (see Section 3: DNA extraction, below). These were collected before enforcement of the *Nagoya Protocol on Access to Genetic Resources and the Fair and Equitable Sharing of Benefits Arising from their Utilization to the Convention on Biological Diversity* (https://www.cbd.int/abs/). However, some specimens were obtained more recently through collecting efforts by our team and collaborators. Some countries, such as the USA, Chile, and many European countries, do not require permits for collecting non-protected species outside of protected areas, and some of our specimens were obtained in this manner. Other specimens were obtained in areas where permits were required. We list permits and their relevant countries and regions in Table S34; a pdf of permits can be found in Data S10.

3. DNA extraction

We extracted DNA from 1,915 specimens that were 1) stored in ethanol and frozen; 2) dried and stored in glassine envelopes under ambient conditions (papered); or 3) dried, spread and pinned in a museum collection. Ethanol-preserved specimens were first air-dried on a Kimwipe® before autoclaved forceps and microdissection scissors were used for specimen

dissection. For ethanol-preserved and enveloped specimens, we prioritized tissue from the thorax, but if thoracic tissue was unavailable, we used legs or tissue from the proximal region of the abdomen. We removed abdomens and/or up to three legs from museum specimens. In all cases, when multiple specimens of the same species were available, the most-recently collected specimen was used for DNA extraction. Tissues were transferred to a 1.5 mL microcentrifuge tube before labeled with a unique ID (Table S1).

All tissues were extracted with the OmniPrep DNA Extraction Kit for High Quality Genomic DNA Extraction (G-Biosciences, Cat. #786-136, St. Louis, MO, USA). We used three volumes of lysis buffer (300 µl, 500 µl, and 1 ml) depending on tissue size to ensure tissue submergence. Tissues of the thorax and/or legs were homogenized in a lysis solution consisting of 1:100 Proteinase K to lysis buffer using sterilized chrome beads and a Mini-Beadbeater-96 (BioSpec.com). For pinned museum specimens, the abdomen was soaked in a solution of 1:50 Proteinase K to lysis buffer. Following Hamilton et al.[17], all tissue digestions were incubated at 56° C for up to 24 hours.

After incubation, samples were vortexed, centrifuged, and heated to 25° C. Chloroform (100 µl) was added to each sample before vortexing until a uniform, milky-white color was achieved. The solution was centrifuged at 14,000 RCF for 10 minutes, the resulting supernatant containing DNA was transferred to a new microcentrifuge tube, and the lower phases including the exoskeleton were discarded. Subsequent reagents were added in proportion to the volume of lysis buffer used per the manufacturer's protocol. The following steps are based on a standard 300 µl lysis buffer volume. First, 25 µl of DNA stripping buffer was added to each sample before being mixed, centrifuged, and incubated for another 10 minutes. Precipitation buffer (50 µl) was added to each sample, with an additional 50 µl added to samples that did not immediately show precipitation. Mussel glycogen (3 µl) was added to each sample to improve yield. Next, samples were vortexed and centrifuged at 14,000 RCF for 20 minutes. After centrifugation, all liquid was pipetted into a sterile microcentrifuge tube and the precipitate was discarded. Cold isopropanol (250 µl, -20° C) was added to each sample before samples were vortexed and centrifuged. Samples were stored at -20° C for at least 30 minutes and up to 24 hrs before being centrifuged at 14,000 RCF for 10 minutes to precipitate DNA. Once centrifugation was complete, the isopropanol solution was discarded, leaving only a solid pellet of DNA at the bottom of the extraction tube. Cold 80% EtOH (300 µl, -20° C) was added to each sample before

centrifugation at 14,000 RCF for 10 minutes. EtOH was removed without disturbing the DNA pellet and pellets were air dried in their tube to evaporate remaining ethanol. Once dry, 50 µl of TE buffer and 0.5 µl 5 mg/ml RNAse (G-Biosciences, Cat. # 786-040) was added to digest RNA. Samples were either incubated overnight at 4° C to allow DNA pellets to resuspend naturally into the TE buffer or directly placed into -20° C storage. All DNA extractions were stored in 1.5 mL microcentrifuge tubes at -20° C prior to sequencing. Most extractions were performed at the McGuire Center for Lepidoptera and Biodiversity, Florida Museum of Natural History, University of Florida; the Museum of Comparative Zoology, Harvard University; or the Biology Department, City College of New York, City University of New York. Chinese samples that could not be exported to the US were sequenced at Annoroad Gene Technology Co., Ltd, Beijing, China.

4. DNA quantification and sequencing

We quantified DNA with a Qubit 2.0 Fluorometer using a Double Stranded DNA Broad Range and High Sensitivity Assay Kit (Thermo Fisher Scientific, Cat. #Q32850 and #Q32854, respectively). When DNA was undetected using the broad range kit, the extract was subsequently quantified using the high sensitivity DNA assay kit. All assays were incubated for 3 minutes with 1µl of template before fluorescence was measured and concentration calculated. The extent of DNA fragmentation was assessed by electrophoresis on a 2% agarose gel with 2.5 µl of 10,000x SYBR Green I Nucleic Acid Gel Stain added to the agarose solution before the gel was poured and set. All samples were loaded into the gel with a mixture of 4 µl DNA extract, 1 µl of 5x DNA Loading Buffer (Bioline, Cat. # BIO-37045), and 1 µl 100x SYBR™ Green I Nucleic Acid Gel Stain in DMSO (Thermo Fisher Scientific, S7567). The molecular weight of DNA fragments was determined with 5 µl Hyperladder II 50 bp Ladder (Bioline, London UK), loaded in a single well on each row. All gels were run at 100 volts for 2 hours. Sample quality was classified as either degraded (all smears < 1000 bp) or high molecular weight gDNA (some DNA > 1000 bp).

Samples prepared with at least 250 ng were loaded onto 96-well plates arranged from highest to lowest DNA concentration and sent to RAPiD Genomics (Gainesville, FL, USA). RAPiD Genomics quantified total gDNA using a Quant-iT™ PicoGreen™ double stranded DNA Assay Kit (Cat. #P7589; Turner Biosystems). Samples containing < 300 ng of total DNA were

flagged by RAPiD for review before proceeding with library preparation. While 300 ng of starting material was optimal for library preparation, in some instances low-quality extracts with < 30 ng of DNA were submitted and sequenced.

Prior to library preparation, DNA with fragments of >1000 bp were sheared to an average size of 300 bp using a Covaris E220 machine. Low-quality, highly fragmented samples did not undergo shearing. All samples underwent end-repair and ligation of an adenine residue to the 3' end of the blunt-end fragments to allow ligation of barcoded adapters and PCR-amplification of the library. Custom BUTTERFLY1.0 SureSelect probes[6] (Agilent Technologies) were used in conjunction with a SureSelectXT Target Enrichment kit for target enrichment of 16 pooled libraries before multiplex sequencing on an Illumina HiSeq 3000 to generate 150 -bp paired-end reads. Samples extracted in China underwent library prep, target capture, and sequencing at Annoroad Gene Technology Co (Beijing, China), following the same protocols.

5. Sequence assembly and clean-up

Locus assembly and sequence clean-up followed the pipeline of Breinholt et al.[18]. Illumina sequences were quality-filtered with Trim Galore! v. 0.4.0[19] allowing a minimum read size of 30 bp and trimming to remove bases with a Phred score < 20. For each sample, only sequences with forward and reverse reads that both passed quality filtering were included. All loci were assembled using iterative baited assembly (IBA)[18]. Sequences from the probe region were mapped to the *D. plexippus* genome v.3[20] to determine single hits and orthology. Orthologs were screened for contamination, and sequences with 99% match to a butterfly family that was not of the sample were removed. Loci were aligned with MAFFT v. 7.0.1[21] and a strict consensus generated using FASconCAT-G v. 1.0.4[22] for species with multiple isoforms. Isoforms were excluded in rare cases where a consensus among isoforms could not be determined due to non-homology.

6. Extraction of BUTTERFLY1.0 loci from genomes and transcriptomes

Published sequences were obtained from 1) genome assemblies; 2) genomic reads; and 3) paired or 4) single-end transcriptomes. For genome assemblies, NCBI was searched for the newest assembly, and newly submitted assemblies were preferred (see Table S1 for accession numbers). Additionally, the NCBI SRA database (https://www.ncbi.nlm.nih.gov/sra) and

published literature[3,12,23–40] were searched for available butterfly genomes and transcriptomes, prioritizing type species. We also included available sequences of ten moths from 9 families: Cimeliidae, Drepanidae, Erebidae, Geometridae, Noctuidae, Plutellidae, Saturniidae, Sphingidae, and Tortricidae. For each of these superfamilies, NCBI was searched for available genomes and transcriptomes, and representative species with the greatest amount of sequence data were chosen. All searches were performed in October 2019. These families were chosen based on availability of data and their phylogenetic placement in relation to butterflies[12].

Genomic and transcriptomic reads were trimmed with TrimGalore!, requiring the same trimming threshold as explained above. The IBA_single.py script from Breinholt et al.[18] was adapted to accommodate single-end reads (Data S11). Genomic reads were assembled using SPAdes v3.13[41]. The script, genome_getprobe.py[6], was used to extract probe regions from all genome assemblies (for both previously assembled and *de-novo* assemblies), using the *Danaus plexippus* genome v.3[20] as reference. MAFFT v7.294b[21] was used to align and reverse-complement those alignments with respect to the *D. plexippus* probes with the command, '-adjustdirectionaccurately'. At this point, the genomic dataset included Anchored Hybrid Enrichment (AHE) probe orthologs and paralogs.

A mapping approach was used to confirm orthology. We used the ortho_filter.py script[18] and BLAST+[10] to determine whether the probe hit and the reference sequence mapped to the same scaffold and location in the reference genome. *De novo* genome assemblies often produced low N50 scores[42], and these assemblies were removed because they increase the likelihood of erroneously assembled genes and may result in the appearance of high levels of gene duplication (e.g., Denton et al.[43]). To determine orthology of these draft assemblies confidently, our dataset was refined using a gene tree approach. We estimated gene trees in FastTree v2.1.7[44] with a GTR model and used PhyloTreePruner[45] to determine the maximally inclusive subtree for each locus. Locus names and the number of loci captured are shown in Table S35.

<u>7. Dataset construction</u>

All loci were first aligned individually in MAFFT v7.294b[21] using the commands '-ginsi' and '--adjustdirectionaccurately' to assure that the sequences were not reverse complemented. Alignments were trimmed to the original, in-frame probe region taken from the *Danaus plexippus* genome v.3[20]. Each locus was realigned using a codon-aware framework, based on a

standard and invertebrate mitochondrial translation made in MACSE v2.03[46]. Trimal v1.2[47] was used to remove non-homologous gaps with a gap threshold of 0.25, followed by manual inspection and trimming of each alignment in AliView v1.26[48]. A decision-making flowchart including how minor manual alignment adjustments were made is provided in Data S12. For each locus, internal stop codons were replaced with "NNN" in the nucleotide alignments. A locus was removed if it had < 60% taxon coverage of the 2,258 terminals. Nuclear and mitochondrial loci were translated to amino acids in MACSE v2.03[46]. Nucleotide and amino acid concatenated alignments were generated with the Perl script, catfasta2phyml.pl[49], which resulted in a final, concatenated alignment of 391 loci.

8. Sequence quality check

Multiple tests were conducted to determine whether sequences were contaminated. All samples that were included in the final dataset were checked first by BLAST, comparing its COI locus via the command-line blastn in Genbank. A minimum e-value of 0.0001 and maximum of five target sequences were specified to filter the search. The length of the sequence matching the query, its bit score, and taxonomy of resulting matched sequences were used to determine contamination. Samples with a low bit score were flagged, and if the top three hits for that sequence was a species in a different butterfly family, the voucher specimen was examined for confirmation. Identification was confirmed by sharing specimen images with taxonomists, and in some cases, genitalia were dissected for verification.

We took three additional steps to identify contaminated or mislabeled sequences. (A) We generated gene trees in FastTree v2.1.7[44] for every locus. Along with visual inspection, branch tip lengths were compared in all gene trees using LongBranchFinder.pl[50]. If a particular gene tree branch was found to be significantly long (i.e., ≥ 8 SD from the mean branch length) the corresponding sequence for that taxon was removed from that locus (Table S36). (B) The R package MonoPhy v1.3[51] was used to perform a thorough search for any non-monophyletic groups present in an initial unpartitioned ML tree generated in IQ-TREE[52], using our subfamily, tribe, and genus-level butterfly taxonomy. Questionable placements of taxa in the tree were verified by expert taxonomists and removed if there was a significant concern for contamination. (C) Quartet sampling of internal node scores[53] was conducted on an initial unpartitioned ML tree

that was generated in IQ-TREE. We calculated Quartet Fidelity (QF) scores[53] (Table S37, Data S13) and specimens that were deemed outliers were removed.

9. Datasets, partitioning and model selection

Three sequence datasets were created for this study: a nucleotide dataset with all codon positions (nt123), a nucleotide dataset that excludes all synonymous changes (degen), created using the Perl script, Degen1 v.1.4[54,55]), and an amino acid dataset translated from the nt123 dataset (aa; Data S3). The nt123 dataset was analyzed three times with different partitioning schemes: 1) unpartitioned, 2) 6 partitions, and 3) 321 partitions determined by PartitionFinder2[56]. The degen dataset was similarly analyzed with three different partitioning schemes: 1) unpartitioned, 2) 6 partitions, and 3) 359 partitions determined by PartitionFinder2. The aa dataset was analyzed with two different partitioning schemes: 1) unpartitioned and 2) 154 partitions, also determined by PartitionFinder2. For the two analyses with 6 partitions, datasets were partitioned by the two sequence types (mitochondrial, nuclear) and three codon positions. For datasets with a greater number of partitions, we selected optimal partitioning schemes using the corrected Akaike Information Criterion in PartitionFinder2 and by pre-defining each gene as an input data block (defined in Table S38). We used the commands: '--raxml'[57], the 'rclusterf' searching algorithm[56], linked branch lengths, and a user defined starting tree ('user_tree_topology' option) estimated in FastTree v.2.1.7[44]. We used the GTR + G and LG + G models for the nuclear and amino acid datasets, respectively. After running PartitionFinder2, we optimized the substitution model selection for each meta-partition with ModelFinder Plus (command '-m MFP') in IQ-TREE v2.0.4[52]. Partition finding and model selection were performed on the UF High Performance Cluster, "HiPerGator2 (www.rc.ufl.edu/services/ hipergator/), the Brigham Young University (BYU) High Performance Cluster (https://rc.byu.edu/) and Smithsonian Institution High Performance Cluster (SI/HPC; https://confluence.si.edu/display/HPC/). Model selection results for all eight dataset-partition combinations are shown in Table S38 (See also Data S14).

10. Tree inference and branch support

Maximum likelihood (ML) tree inference was conducted on all three datasets (nt123, degen, and aa), and parameter settings for each analysis are shown in Table S14. For the nt123

and degen datasets, starting trees were generated in FastTree v2.1.7[44] under the Jukes-Cantor + CAT model. For unpartitioned analyses (analyses 1 and 4, Table S14), ML tree searches were performed in IQ-TREE v2.0.3[52] under a GTR + I + G model, using the '-bnni' option to improve the best tree search and to reduce the risk of overestimating ultrafast bootstrap branch support. For the 6-partition analyses (Table S14), multiple independent ML tree searches were performed: 15 searches for the nt123 dataset, and 16 for the degen dataset (hereafter called "degen6"). These tree searches were performed in IQ-TREE v2.0.3 using the partitioning scheme and models obtained previously (see above), and a starting tree generated by FastTree. The tree with the highest log-likelihood was selected as the best tree and was used as the starting tree for the unpartitioned nt123 analysis and the degen analysis with 359 partitions (hereafter called "degen359"). For the 321-partition, nt123 analysis, four independent ML tree searches in IQ-TREE v2.0.3 were conducted; for degen359 (Table S14), two independent ML tree searches were performed (see *Section 9. Datasets, partitioning and model selection*). Both trees utilized the 6-partitioned analysis ML tree as the start tree.

For the aa unpartitioned analysis (Table S14), 17 ML tree searches were performed in IQ-TREE v2.0.3 with the LG4X model. The tree with the highest log-likelihood was selected as the best tree, and it was used as the starting tree for an additional ML tree search using the same model. We applied nearest neighbor interchange (NNI) branch swapping to each bootstrap tree to limit overestimating ultrafast bootstrap branch supports ('-bnni' command)[58]. For the 154-partition amino acid analysis, hereafter called "aa154" (Table S14), an ML tree search was performed in IQ-TREE v2.0.4[52] using the same partitioning scheme and models that were determined in PartitionFinder2[56].

Branch support was calculated with 1,000 ultrafast bootstrap replicates (UFBS; '-B 1000' command)[59,60], and Shimodaira-Hasegawa approximate likelihood ratio tests (SH-aLRT; '-alrt 1000' command)[61]. Branches were classified as "robust" if they were recovered with support values of UFBS $\geq$ 95 and SH-aLRT $\geq$ 80, for comparative purposes with other phylogenetic studies on butterflies that utilized these branch support metrics (e.g., Espeland et al.[6], Toussaint et al.[62]). These analyses were run on the University of Florida HiPerGator2 Cluster (www.rc.ufl.edu/services/hipergator/), Brigham Young University (BYU) High Performance Cluster (https://rc.byu.edu/) and Smithsonian Institution High Performance Cluster (SI/HPC; https://confluence.si.edu/display/ HPC/).

Quartet sampling (see *Section 8. Sequence quality check*) was performed on degen359 and aa154 trees with the highest likelihood score. We calculated the Quartet Concordance (QC), Quartet Differential (QD), and Quartet Informativeness (QI) values for each internal node[53] (Tables S39-S40, Data S15-S16).

11. Coalescent-based methods

Because concatenation has been shown to fail when incomplete lineage sorting is common[63], we assessed the impact of potential gene tree discordance[64,65] by inferring a tree for each locus (nt123 dataset). We used IQ-TREE 2.0 -rcl[52] to build single locus trees and compute tree estimation in ASTRAL-III 5.7.3[66]. We calculated local posterior probability (LPP[67]) values in ASTRAL to evaluate node support on the species tree. LPPs were determined to be "robust" if branches were recovered with values ≥ 0.95. Because sequences from the same gene were split into multiple loci and placed in different parts of the concatenated datasets (see above), these loci were merged, resulting in a set of 326 loci for gene-tree estimation, reduced from the 391 in concatenated datasets. Model selection and tree searches for each gene were performed using default settings in IQ-TREE (i.e., the '-s' command with no further parameters specified). These analyses were conducted on the University of Florida HiPerGator2 Cluster (www.rc.ufl.edu/services/hipergator/).

12. Topology tests

Four-cluster likelihood mapping (FcLM) analyses[68] were performed on the degen and aa datasets to assess the placement of particular butterfly clades that have been the subject of previous phylogenetic studies. We applied this approach in addition to standard branch support metrics because the latter can be subject to inflated estimates[68].

Four hypotheses were tested with each dataset, for a total of eight FcLM analyses. For these tests, we defined four clades of interest *a priori*. Due to the large number of terminals in the datasets, groups that exceeded 500 species were pruned to assure analysis completion. Lists of species present in each test clade are provided in Table S41, and additional input files are provided in Data S17. The four hypotheses, and summaries of how clades are defined, are as follows:

12. 1. Position of the nymphalid subfamily Libytheinae:

The position of Libytheinae as the sister group to Danainae contradicts the findings of Wahlberg et al.[69] and Espeland et al.[6].

H_0: Libytheinae and Danainae form a monophyletic group that is sister to all other Nymphalidae.

H_A: Libytheinae is not the sister group to Danainae. Instead, Danainae is sister to all other Nymphalidae, and Libytheinae is the sister group to Danainae + remaining Nymphalidae (a relationship supported by Espeland et al.[6]).

Cluster 1: Libytheinae (3 species)
Cluster 2: Danainae (60 species)
Cluster 3: Other Nymphalidae (74 species, reduced from 580 species)
Cluster 4: Outgroup (151 species, reduced from 1605 species)

12. 2. Placement of the hesperiid subfamilies Barcinae and Malazinae:

H_0: Barcinae + Malazinae is monophyletic.

H_A: Barcinae + Malazinae are not sister groups to each other, Barcinae forms a monophyletic group with Trapezitinae.

Cluster 1: Malazinae (1 species)
Cluster 2: Barcinae (2 species)
Cluster 3: Trapezitinae (25 species)
Cluster 4: Outgroup (228 species, reduced from 2220 species)

12. 3. Monophyly of Papilioninae:

H_0: The four tribes of Papilioninae form a monophyletic group.

H_A: Leptocircini is not the sister group to the remaining three papilionine tribes. It is sister to a clade containing both the other Papilioninae and Parnassiinae.

Group 1: Leptocircini (14 species)

Group 2: Other Papilioninae (43 species)

Group 3: Papilionidae: Parnassiinae (14 species)

Group 4: Outgroup (219 species, reduced from 2177 species in original dataset)

12. 4. Tribal relationships of Pierinae:

Tribal relationships within Pierinae conflict with Wahlberg et al.[70]. Placement of three pierine tribes (Anthocharidini, Nepheroniini, Teracolini) also differ between the degen359 and aa154 trees.

H_0: Anthocharidini is sister to all other Pierinae.

H_A: Anthocharidini is not the sister group to all other Pierinae. Nepheroniini is the sister group to the remaining Pierinae, with Anthocharidini as the sister group to the remaining four pierine tribes (Elodinini, Leptosianini, Pierini, Teracolini).

Cluster 1: Anthocharidini (10 species)

Cluster 2: Nepheroniini (3 species)

Cluster 3: Teracolini (9 species)

Cluster 4: Outgroup (i.e., other Pierinae tribes) (58 species)

We assessed whether a non-phylogenetic, possibly confounding signal was present in our dataset that could affect our ML tree inference and FcLM results on the 'original' (i.e., non-permuted) data. Sources of possible confounding signal that might violate globally stationary, reversible, and homogeneous (SRH) conditions[71,72] include heterogeneous composition of amino acid sequences (among-lineage heterogeneity) and non-randomly distributed, missing data[73].

We also generated optimized data subsets for each of the four FcLM analyses by including only those partitions of the degen and aa data matrices that contained sequences of at least one representative from the four groups specified for testing each hypothesis. We applied three different FcLM permutation approaches. In permutation I, all phylogenetic signal was removed. In permutation II, compositional heterogeneity was removed by randomly drawing amino acids using the 12 frequencies of the LG substitution matrix, but the distribution of missing data was left untouched. Permutation III included the features of permutation II, but also

had a random distribution of missing data (i.e., no phylogenetic signal, homogeneous composition, and missing data randomly distributed). For additional information on this strategy and rationale, see Supplementary Information in Misof et al.[73] and Kawahara et al.[12].

We used IQ-TREE v1.6.7[58] to calculate all possible quartets and their support values (option '-lmap ALL'). Quartet log-likelihoods were parsed into separate output files (option '-wql'). For the FcLM analyses with the 'original' non-permuted data, we kept partition boundaries and substitution models as with our previous degen359 and aa154 ML analyses. We chose the partitioned approach and allowed partitions to have different evolutionary speeds (option '-spp'). For all permutation approaches, we kept partition boundaries and attempted to perform revised model selections, though this was not done with two of the degen analyses (monophyly of Papilioninae, tribal relationships of Pierinae) because attempts to do so with a partition file in IQ-TREE failed. We used the LG model for all analyses and '-q' (edge-equal partition model) to avoid program crashes. Quartets were mapped in a 2D simplex graph in IQ-TREE. Quartets mapped onto T1 (area 1), T2 (area 2) and T3 (area 3) show unambiguous support for the respective topology; quartets mapped onto T12 (area 4), T13 (area 6), T23 (area 5) are partially resolved; quartets mapped onto T* (area 7) have star-like topologies that remain unresolved (see Fig. S14 and Fig. S15). Topology tests were conducted on the University of Florida HiPerGator2 Cluster (www.rc.ufl.edu/services/hipergator).

13. Divergence time estimation

We obtained divergence time estimates using a penalized-likelihood based approach implemented in TreePL[74]. We used this approach because (A) it has been shown to effectively estimate a range of divergence times (e.g., Sun et al.[75,] Zhang et al.[76]), (B) because our dataset was too large to use with most other programs (e.g., BEAST), and (C) because birth-death models of evolution can potentially lead to misleading results[77] (but see Helmstetter et al.[78,] Morlon et al.[79]). Because butterfly fossils are not abundant, and many have questionable paleontological age estimates[80], we implemented three different methods for calibrating trees as a sensitivity test (see Phylogenetic Analysis and Dating section in Methods). Nodes that were calibrated for all three methods are listed in Table S42.

Since a single TreePL analysis yields only one age estimate for each node, we performed multiple analyses derived from the same dataset to obtain a range of age estimates. We

implemented two strategies for doing so: (A) Run TreePL multiple times using the same starting tree. TreePL is run in three steps: Step One generates a series of parameters that are input into the configuration file used for Step Two, which in turn produces a smoothing parameter that is input into the configuration file used for Step Three. We ran Step One 100 times, found the combination of parameters that appeared in a plurality of output files, and used those for Step Two. We ran Step Two 100 times, found the smoothing parameter that appeared in a plurality of output files, and ran Step Three 100 times with that parameter. This overall analysis was done only on the tree from the amino acid 154-partition dataset, and only with secondary calibrations (Method 1). (B) Resample the original dataset 100 times to generate 100 bootstrap trees in IQ-TREE, then run TreePL on each tree one time, following the protocol of Zhang et al.[76]. IQ-TREE runs had their topologies (but not branch lengths) constrained to ensure that the resulting TreePL runs could be compatible. This analysis was performed 12 times: three topologies (degen6, degen359, and aa154) each calibrated the four different ways described above. Custom Python scripts were generated to partially automate the process of setting up and running TreePL iterations (C. Earl; https://github.com/sunray1/treepl). For both strategies, each set of trees was combined into a single tree file with TreeAnnotator[81], with ranges of age estimates on nodes (Table S4). The tree dated with Strategy B (aa154 dataset, secondary calibrations) was used for analyses in all subsequent sections that required a dated tree as input.

14. Diversification analyses

We performed a Bayesian analysis of macroevolutionary mixtures using the program BAMM v.1.10.4[82] to detect shifts in diversification rates between clades. The reversible-jump Markov chain Monte Carlo (rjMCMC) was run for 50 million generations and sampled every 50,000 generations. Priors were estimated with the R package BAMMtools v.2.1.6[83] using the command ‘setBAMMpriors’. The tree was trimmed in Mesquite v.3.6[84] to remove all outgroups. Six analyses were performed using different priors for expected numbers of shifts (5, 10, 20, 30, 40, and 50 shifts). We analyzed the BAMM output files using BAMMtools with a 10% burn-in. In order to account for differences in sampling among the butterfly genera, we provided a sampling fraction file to BAMM with percentages of species in the tree for each genus as well as an overall genus-level backbone sampling fraction (Data S18, Table S43). Our analyses suggest that a prior of 30 expected shifts was the most probable (see Results), therefore we carried out

the following analyses on the expectedNumberOfShifts = 30 analysis. First, we identified the 95% credible set of rate shifts using the command, 'credibleShiftSet' (Fig. S16) to identify the most probable shift configurations, setting the threshold to 1000 and 'set.limit' to 0.95, which returned only two configurations. The configuration with the highest posterior probability (0.69) had 1 core shift, and the configuration with the next-highest posterior probability (0.31) had no core shifts (Fig. S16). We plotted distinct shifts on the highest probability configuration using 'distinctShiftConfiguration' with 30 expected shifts and a threshold of 1000 (see Fig. S17). Mean speciation rates for clades of interest were retrieved using the 'getCladeRates' command and are reported in Table S44. We also performed the same analysis with a threshold of 500 to confirm consistency in the resulting shift configurations (Fig. S18).

We also conducted a lineage-specific birth-death shift analysis[85] in RevBayes[86]. We chose to run RevBayes as an additional measure to BAMM because the latter approach has been criticized for incorrectly modeling rate shifts on extinct lineages (i.e., extinct or non-sampled lineages inherit the ancestral diversification process and cannot experience subsequent diversification-rate shifts[87,88]. For RevBayes, we removed outgroups but also trimmed ingroup tips to balance species sampling across the tree. We assigned a lognormally distributed variable for the mean speciation and extinction rates, an exponentially distributed variable for the rate of rate-shift events, and six rate categories, largely following Höhna et al.[85]. An MCMC chain of 5,000 steps was performed (Data S19, script modified from the original in GitHub, https://revbayes.github.io/tutorials/divrate/branch_specific.html). To verify that there were no other factors affecting our results, we ran an additional BAMM analysis as described above but with the same tree used for RevBayes and without a fraction file.

15. Larval host dataset assembly and curation

Larval host records were compiled from nine different sources, including: 1) the Database of the World's Lepidopteran Hostplants (HOSTS)[89], which summarizes data from ~270 other sources; 2) the *Lepidoptera and other life forms* database, (http://ftp.funet.fi/index/Tree_of_life/insecta/lepidoptera/); 3) 40 years of food plant rearing records from Costa Rica by Daniel Janzen, Winnie Hallwachs and colleagues (http://janzen.sas.upenn.edu/); 4) the ButterflyNet Trait Database (https://butterflytraits.org/) which included host records from 109 butterfly field guides and other resources; 5) a

comprehensive database for host records for all butterflies in Japan[90]; 6) a set of papers documenting the hosts of butterflies in India[91–97]; 7) a database of hosts and ant symbionts of larval Lycaenidae and Riodinidae compiled from 85 literature sources by Naomi E. Pierce and her lab members; 8) a database of butterfly host records from Ecuador based on field observations and literature records compiled by Keith R. Willmott; and 9) 88 papers from the primary literature or relevant websites (Table S9, Data S6). Whenever possible (e.g., in the ButterflyNet trait database that was initiated for this project), we retained the following information for each host record, if available: 1) the taxon and taxonomic authority of butterfly to the lowest available taxonomic level (family, subfamily, tribe, genus, species, or subspecies); 2) the taxon and taxonomic authority of host to the lowest available taxonomic level (family, genus, species, subspecies or variety); 3) plant part eaten; 4) record certainty (e.g., novel plant accepted in captivity, oviposition record with no observation of herbivory, etc.); 5) geographic location of observation; 6) relevant information on all non-plant hosts. The extensive data recorded in the host (food plant) database of Janzen, Hallwachs, and colleagues was simplified to retain the fields of butterfly genus and specific epithet; plant family, genus, and specific epithet; together with indication of whether the plant was introduced to Costa Rica. This database contains many records of informal, non-code-compliant names of butterfly cryptic species. Rather than discarding the large number of records that would not be compatible with any other data source, we regarded these as the nominal species (e.g., *Battus polydamas* instead of *Battus polydamas*DHJ01). The number of records for each butterfly species x plant species interaction was recorded.

Data from these disparate sources were harmonized into a single flatfile database of records. Next, butterfly species names were validated using Taxotools[4] to conform with a uniform, standard taxonomy based on our list of valid butterfly names. This R library was also used in conjunction with a master list of plant taxonomy and synonomies derived from World Flora Online[98] to standardize plant taxonomy and lookup valid families and orders of plant genera. When hosts were recorded as common names (e.g., grass, Job's Tears, ant larvae), we used a database of plant common names by Robert P. Guralnick to convert these records to the lowest possible taxonomic resolution (e.g., Poaceae, *Coix lacryma-jobi*, Formicidae). Few Hemiptera eaten by butterfly caterpillars were identified to the species level, but these names were validated to the best of our abilities. The Aphid Species File (http://aphid.speciesfile.org),

AntCat (https://www.antcat.org), and ScaleNet (https://scalenet.info) were used to validate names of aphids, ants, and scales respectively. After name validation, our database had 66,397 unique butterfly species x plant species x data source records. The same interaction was often recorded in multiple data sources. For subsequent analyses on the evolution of host use, we only retained data for the butterfly species that were included in our final tree. For species that lacked host records, we did not attempt to summarize data on hosts used by other species in its genus or impute interactions based on records of closely related butterfly species. Host records for the butterfly species in our tree were extracted from the full database for further scrutiny and addition of host records from other sources. In an attempt to make our dataset as complete as possible, we determined which species in our tree lacked host records and conducted Google Scholar searches for each species using search phrases including the butterfly's species name: *Genus species* hostplant; *Genus species* hostplant; *Genus species* larval food plant; and *Genus species* life history. Host records for more than 100 species that lacked data were added from 88 web pages and peer-reviewed journal articles. Host data were extracted from these sources, names were validated, and added to the database.

Documenting larval host records is challenging because it requires taxonomic knowledge of at least two kingdoms, time (since most larvae cannot be identified and the insect must be reared through eclosion), and luck—larvae and pupae can be killed by parasitoids or viruses, eat all of the available host and starve, etc. Many aggregations of host records, notably the HOSTS database by Robinson et al.[89] and the published books derived from it[99] do not attempt to scrutinize the veracity of host records and thereby propagate erroneous records that are difficult or impossible to verify. We noticed a large number of improbable records derived from the HOSTS database. For example, it is widely accepted that species in the pierid genus *Delias* are all specialized on mistletoes (Santalaceae and Loranthaceae), which are specialized epiphytic parasites of various trees. Similarly, all known Miletinae (Lycaenidae) feed on living insects or products derived from them, and Hemiptera are frequently eaten. The HOSTS database included multiple erroneous records that were almost certainly the hosts of these mistletoes and Hemiptera, respectively. We therefore flagged these records as dubious together with records of angiosperm feeding in Liptenini (Lycaenidae: Poritiinae), where all species with known life histories are otherwise known to feed solely on lichens or cyanobacteria[100].

Given the size of our host datasets and the scale of our analyses, we chose to examine relationships between individual butterfly species and host families that are consumed by their larvae. Plant families are commonly adopted as the taxonomic rank used for examining the evolution of host use[101,102]. By reducing the complexity of the dataset through collapsing host genus, species, and infraspecific taxa into families, we were able to aggregate and summarize records in a way that allowed us to highlight potentially erroneous records. To flag records of "taxonomic outliers" (a record for a host taxon distantly related to other recorded hosts), we calculated three values associated with each butterfly species x host family record: 1) the number of literature records for this interaction; 2) the proportion of host records for that butterfly species that are from plants in the same host order as the current record ("ordinal proportion"); 3) the proportion of host records for that butterfly species that are from plants in the same host family as the current record ("familial proportion"). Carefully curated records of Janzen, Hallwachs, and colleagues from Costa Rica were regarded as error-free regardless of their ordinal and familial proportions because of the high degree of taxonomic scrutiny given the specimens in this ongoing study and because the large number of rearing events (>45,000 records of <600 butterfly species) meant that even rare butterfly-host associations had usually been recorded multiple times. We scrutinized individual records in which the ordinal proportion was <5%, indicating that 95% of host records are from plants in a different order. Suspicious records were examined and removed on a case-by case basis. The number of records for a particular butterfly-plant interaction was used as a heuristic for assessing the number of times a particular interaction had been recorded in the literature.

16. Hosts and phylogenetic distance

For each plant-feeding butterfly species in our tree, we quantified host richness and phylogenetic distance using six different metrics implemented in the package picante v. 1.8.2[103]. We quantified host richness using the number of host families and Faith's phylogenetic diversity (PD), the latter which is the sum of phylogenetic branch lengths[104]. We quantified host divergence using three values: 1) the mean pairwise distance (MPD), or the average distance separating all pairs of species on the phylogenetic tree[105]; 2) the mean nearest taxon distance (MNTD) which is the mean distance between each species and its closest

relative[105]; and 3) the distance-based speciation index (DSI), which is a *z*-score of MPD or MNTD which measures specialization as a deviation from a random expectation[106].

To calculate these metrics, we used the calibrated tree of seed plants from Smith and Brown[107]. We pruned the tree to include two species per plant family whose divergence time represents the crown group age for each family. This exercise enabled the calculation of pairwise distance-based measures such as MPD, MNTD, and DSI for monophagous butterflies that feed on only one plant family. The functions 'pd', 'ses.mpd', and 'ses.mntd' were used to calculate these phylogenetic metrics and their statistical significance using 1000 replicates in picante. The R script is available on GitHub (https://github.com/lmcai/BNet-PD-analysis).

17. Ancestral state reconstruction

Because the number of host groups in our dataset was too large for an ancestral state reconstruction (nearly 50 host orders, ~200 plant families plus insect associations), we first reduced the number of host groups by using a network analysis. The Beckett algorithm[108], as implemented in the function 'computeModules' from the package bipartite[109] in R v. 3.6.2[110], assigns plants and butterflies to modules and computes the modularity index, Q. By maximizing Q, the algorithm finds groups of butterflies and hosts that interact more with each other than with other taxa in the network. Thus, hosts that are assigned to the same module tend to be used by the same butterflies. We found 13 modules for butterfly host associations in our module analysis (Tables S32-S33).

We conducted three larval host ancestral state reconstruction analyses using stochastic character mapping with SIMMAP in Phytools v.0.7-70[111] using the 'make.simmap' command. We reconstructed the ancestral state of (A) generalist versus specialist feeding (2 states, Data S7), (B) plant, lichen, Hemiptera, or Hymenoptera as a food source (4 states, Data S8), and (C) plant module (13 states as described above, Data S9). For C, we assigned probabilities to modules based on the proportion of interactions of a given butterfly with different plant modules. All reconstructions were carried out using a symmetrical model ("SYM") and 1000 simulations (nsim = 1000). The presence of a state was coded as 1, and the absence as 0. When there were missing data, all tested states were coded with equal probability.

The plant module ASR involved 13 possible character states, one for each host module. For each tip on the tree (i.e., each butterfly species in the analysis), all 13-character states were

assigned probabilities based on the modules that the butterfly's hosts belonged to (Tables S9, S33). If a species only fed on host species in Module 1, a probability of 1 was assigned to the M1 state, and probabilities of 0 were assigned to all other states. If a species fed on one host species in Module 1, and one host species in Module 2, then those states were assigned probabilities of 0.5, and all others 0 (regardless of how many individual records/observations were in the literature for each of those species). If a species fed on two host species in Module 1, one species in Module 2, and one species in Module 3, the assigned probabilities were 0.5 for M1, 0.25 for M2, 0.25 for M3, 0 for all others. If no host data were available for a species, all states were assigned equal probabilities (~.08).

18. Model-based comparisons of speciation rate in butterflies and angiosperms

We conducted a series of diversification analyses to evaluate whether there is a correlation between butterflies and plants. For this, we used HiSSE (Hidden State Speciation and Extinction) and a BiSSE-like (Binary State Speciation and Extinction) implementation of HiSSE[112] in the R package hisse[113]. We pruned outgroups from the tree (aa154 dated tree, Strategy A) and compared 20 HiSSE models and BiSSE-like implementations of HiSSE. The BiSSE equivalent of HiSSE tests whether there are different diversification rates associated with the two host use states. Other models were built in the HiSSE framework to test alternative combinations of presence or absence of hidden state and host use associations while also considering different transition rate matrices, net turnover rates τi (speciation plus extinction: $\lambda i + \mu i$), and extinction fractions εi (extinction divided by speciation: $\mu i/\lambda i$) (Table S15). We disallowed dual transitions that involve both observed (host) and hidden states.

We tested whether diversification rates were linked to feeding (A) as a larval specialist or generalist (Table S16), (B) on Poales (Table S17) in Papilionoidea, Hesperiidae, and Nymphalidae, (C) on Fabales (Table S18) in Papilionoidea and Nymphalidae, (D) on Brassicales (Table S19) in all butterflies and Pieridae, (E) on Fagales (Table S20), (F) on the Poaceae module (Table S21), (G) on the Fabaceae module (Table S22), and (H) on Fabaceae in Eudamini (Table S23). E-G were tested for all butterflies (Table S23). For Analysis A (specialists versus generalists), we defined a specialist as feeding on one plant family and generalist as feeding on more than one larval host family following Futuyma[114] (see section above for methods on aggregating larval host data). All analyses were coded as binary: (1): feeding on the host

order/family, (0) feeding on other plants. For the generalist-specialist analyses, generalist feeders were coded as 1 and specialists as 0. In all cases, missing data were coded as a third state, which HiSSE treats as uncertain.

Fraction files of clade-based taxonomic diversity estimates were created for all HiSSE runs to account for taxonomic sampling bias (Table S24). We set the total number of extant butterfly species as 19,500, which is an ~8% increase from the butterfly species richness estimate of van Nieukerken et al.[2]. We added this diversity correction based on the many recent new butterfly species descriptions (e.g., Cong et al.[3]) and morphospecies that we are aware of that have not yet been formally described. We estimated the total number of generalist and specialist species by calculating the percentage of generalists and specialists in our dataset at the family level, standardizing for the proportion of species richness comprised by that family compared to all butterflies, based on diversity estimates of van Nieukerken et al.[2]. For example, 78.61% of all sampled Hesperiidae that had host data were specialists, and Hesperiidae comprise 21.91% of all butterfly species richness, thus we estimated Hesperiidae specialists as 19500*0.2191*0.7861 = 3359 species. Applying these calculations for all families yields totals of 12,969 specialist species and 6,531 generalist species (Table S25); these numbers were used to estimate the fraction of generalists and specialists in our dataset.

Calculating the fraction of species sampled within each host module proved more challenging. To estimate the true butterfly species richness for each module, we used unpublished estimates of species richness for all butterfly genera by co-author Lamas and assumed that if a species was known to belong to a module, so would some of its congeners. These calculations were revised because some genera have large host ranges with species assigned to multiple modules. For example, the 21 species of *Papilio* with host records in our dataset were assigned to three different modules. Since there is an estimated total of 153 *Papilio* species, we calculated that approximately 153/3 = 51 *Papilio* species belong in each of those modules. Calculations for all genera in all modules, and the resulting estimates of module totals and fractions sampled, are provided in Table S26.

19. Coding and curation of distribution data

To reconstruct the biogeographic history of butterflies, we aggregated global distribution data from multiple sources to create a global butterfly checklist for each country. Data sources

included: 1) the *Lepidoptera and other life forms* database (http://ftp.funet.fi/index/Tree_of_life/insecta/ lepidoptera); 2) WikiSpecies (https://species.wikimedia.org); and 3) the type locality of each species or subspecies in our list of valid butterfly names, which was obtained from 1, above. This initial global checklist was vetted using published country checklists and the ButterflyNet Trait Database (https://butterflytraits.org). Trait data from *ca*. 100 comprehensive and country-specific field guides have been entered into this database, allowing us to generate species lists to cross-validate checklists assembled.

We designated 14 biogeographic regions across the globe (Fig. S11, Table S27), determined which of these regions were occupied by each species in our tree, and developed a 14-state character matrix. We created supplementary matrices with broader bioregion characterization: one matrix with seven bioregions, and another with eight bioregions (Figs. S12, S13, Table S27). These correspond closely to the biogeographic realms defined by Udvardy[115], and enabled us to conduct analyses that would have otherwise required too many computational resources with a 14-state matrix (e.g., BioGeoBEARS analyses).

To investigate the biogeographic history of butterflies, we used butterfly country records assembled previously to create preliminary bioregion and tropicality assignments for each butterfly species. Assembled country and bioregion distribution data were screened for errors by manually examining species with unexpectedly disjunct distributions (i.e., species recorded as occupying only two non-adjacent bioregions). The most common errors were species incorrectly recorded as being in Europe (e.g., Belgium, France, Germany, Ireland, Italy, Netherlands, Portugal, Spain, UK) when they were found in a former colony of a European country, or species found in French overseas departments and collectivities (e.g., French Guiana, New Caledonia) being recorded in France. Other errors were caused by similarity of locality names in different bioregions (e.g., the US state Georgia, which is also the name of a country, and Indiana, which contains the name “India”).

Six countries (Canada, China, Indonesia, Mexico, Russia, United States) spanned two or three bioregions, which required manual evaluation of whether species in these countries were found in one or more of the adjoining bioregions. US and Canadian species were assigned to East and/or West Nearctic bioregions based on the paleogeographic history of North America (i.e., whether the species were east or west of the continental divide), with reference to locality

records from Butterflies and Moths of North America (https://www.butterfliesandmoths.org). Russian species were assigned to East and/or West Palearctic bioregions based on locality records assembled by the *Lepidoptera and other life forms* database[116]. Some countries did not have complete distribution lists and were thus evaluated manually by co-authors. Chinese species were assigned to East Palearctic and Oriental bioregions by Houshuai Wang. Indonesian species were assigned to Oriental, Wallacean, and Australian bioregions by David J. Lohman. Mexican species were assigned to East Nearctic, West Nearctic, and Central American bioregions by José Martínez.

The majority of butterfly species are distributed in fewer than five bioregions. Some species are more widespread, but this is often due to recent anthropogenic introductions. Consequently, a final round of data cleaning was performed in which all records of species found in at least five bioregions were manually verified and edited to accurately reflect the species' true native ranges. Cleaned bioregion and tropicality data were converted to character matrices to be used for subsequent distribution analyses (Tables S28-S29).

<u>20. Model-based comparisons of biogeographic patterns and evolutionary rates</u>

To estimate ancestral ranges, we ran the DEC eXtended (DECX) model[117]. DECX is derived from the Dispersal, local Extinction and Cladogenesis (DEC) model[118] which uses a flexible Continuous Time Markov Chain framework to understand the links between dispersal, local extinction and cladogenesis. DECX is superior to DEC because it accounts for rapid expansion and local extinction as possible anagenetic events on the tree and assumes that vicariance is a cladogenetic event without increasing model complexity[117]. To run DECX, we built adjacency matrices with connectivity between any two bioregions defined in the analysis. Each analysis had seven adjacency matrices representing bioregion connectivity at seven different intervals of evolutionary time (aka 'time slices'). These time slices were defined by the following intervals, in Ma: 0-5.33, 5.33-23.03, 23.03-33.9, 33.9-56.0, 56.0-66.0, 66.0-100.0, 100.0-145.0. We ran DECX analyses with 14-area adjacency matrices and with 8-area matrices, using the 14- and 8-bioregion schemes in Table S28. We also ran a second 14-area DECX analysis that excluded the genus *Baronia*, in order to evaluate potential impacts of this relict, Mexican endemic taxon on the results. Files are provided in Data S20.

A geographic and hidden-state speciation extinction analysis (GeoHiSSE) was performed with the Hisse R package[112] to examine the relationship between butterfly diversity and geographic distribution. Tropicality character matrices were used for this analysis instead of the 14-bioregion matrix because, unlike DECX, GeoHiSSE can only be used with a binary character. Three different matrices were generated, each with a different threshold for the % tropicality of a species' estimated range deemed to be sufficiently tropical: a 50/50 matrix where species with at least 50% tropicality were considered tropical, a 30/70 matrix where the cutoff for tropicality was at least 30% tropical, and a "pure temperate" matrix where any species with at least some tropical habitat was coded as tropical, and only species fully endemic to temperate regions were coded as temperate (Table S29). Four models were tested for each analysis, which are outlined in Table S45. In order to calculate sampling fractions for these analyses, we determined the geographic ranges of unsampled species (those that were not in our tree), and assumed that the ratio of temperate to tropical species would be comparable to the ratio of Holarctic (i.e., West/East Nearctic and West/East Palearctic) to non-Holarctic species, which had been calculated previously (see above). This led to an estimate of 2,555 temperate and 16,945 tropical butterfly species worldwide. Ancestral state reconstructions (which do not require sampling fractions) were conducted using each of the three matrices following the protocol described previously (SIMMAP, symmetrical model, 1,000 simulations), and the SIMMAP results were compared to GeoHiSSE results. Finally, to determine whether treating % tropicality as a continuous character would yield results that support the DECX and GeoHiSSE results described previously, an ancestral state reconstruction of tropicality was independently conducted with 1,000 simulations using the contMap command in Phytools[111].

An ancestral range reconstruction for butterflies was conducted using the R package BioGeoBEARS v.1.1.2[119]. Analyses were performed under the Dispersal Extinction Cladogenesis (DEC) model[118,120] and the Likelihood equivalent of the Dispersal-Vicariance approach (DIVALIKE)[121]. A third analysis was conducted under DEC excluding *Baronia*, to assess the impact of this taxon on ancestral ranges. Due to the complexity of our dataset (2,248 tree tips), we restricted the distribution scoring to 7 bioregions: (A) Afrotropics, (E) East Palearctic, (I) Indomalaya, (N) Neotropics, (R) Nearctic, (U) Australasia, and (W) West Palearctic (Fig. S12, Table S28). We also tested an alternative scoring approach for Central America and Caribbean (Fig. S12A, B). The maximum number of areas per ancestral state was

fixed to five. An adjacency matrix was used to specify the allowable areas during each timescale. For that, we used the 7 time slices of the DECX analysis above, except that we added an additional time slice between 0-0.1 Ma so that the adjacency matrix was less strict (Data S3).

To further understand the dispersal process of butterflies over time, we used simulated trees and carried out 100 biogeographical stochastic mappings (BSMs[122]) for each new tree. In all, 100,000 pseudoreplicated biogeographical histories were simulated to estimate the number of dispersal events and *in situ* speciation events. We followed Li et al.[123] to calculate rates of *in situ* speciation, colonization, emigration, and immigration. Speciation rates (λ) for a given location X are represented as $\lambda_X(t1) = s_X(t1)/L_X(t0)$, where s_X denotes the number of speciation events inferred in X in each 0.5-Mya time interval (t1) and L_X denotes the number of inferred lineages in X in the preceding 0.5-Mya time interval (t0), calculated as the cumulative sum of *in situ* speciation and colonization minus local extinction. Colonization rates from location X to location Y during a given time interval t1 are represented as $c_{XtoY}(t1) = d_{XtoY}(t1)/Br(t1)$, where $d_{XtoY}(t1)$ is the number of inferred dispersal events from X to Y and $Br(t1)$ is the total length of all branches in interval t1. Rates of emigration from a given location X and rates of immigration to a given location X, during a time interval t1, are represented as $E_X(t1) = df_X(t1)/Br(t1)$ and $I_X(t1) = dt_X(t1)/Br(t1)$, respectively, where $df_X(t1)$ is the number of inferred emigration events, $dt_X(t1)$ is the number of inferred immigration events, and $Br(t1)$ is the total length of all branches during t1. The output files from these analyses (Data S5) were used to generate the dispersal-rate figures discussed in the main text (Figs. S2-S5, S7, S8)

We performed trait (beta) rate analyses in BAMM to evaluate the number of shifts to tropicality, by running 4 chains simultaneously for a total of 500 million generations, sampling every 50,000 generations, with a 10% burn-in and a Poisson rate distribution of 0.02. We set six different priors for expected rate shifts (1, 25, 50, 100, 150, 200) and compared them using Effective Sample Size (ESS) values. Results were analyzed using BAMMtools. The rate of phenotypic evolution for clades of interest were extracted using the 'getCladeRates' command in BAMMtools.

References

1. Lamas, G. *Atlas of Neotropical Lepidoptera. Checklist: Part 4A. Hesperioidea – Papilionoidea*. 439 (Association for Tropical Lepidoptera, 2004).

2. van Nieukerken, E. J. et al. Order Lepidoptera Linnaeus, 1758. In: Zhang, Z.-Q. (Ed.) Animal biodiversity: An outline of higher-level classification and survey of taxonomic richness. *Zootaxa* **3148**, 212 (2011).
3. Cong, Q., Zhang, J., Shen, J. & Grishin, N. V. Fifty new genera of Hesperiidae (Lepidoptera). *Insecta Mundi* **0731**, 1–56 (2019).
4. Barve, V. *Taxotools: Tools to handle taxonomic lists. R package*. (Zenodo, 2020). doi:10.5281/ZENODO.3934939.
5. Lemmon, A. R., Emme, S. A. & Lemmon, E. M. Anchored hybrid enrichment for massively high-throughput phylogenomics. *Systematic Biology* **61**, 727–744 (2012).
6. Espeland, M. et al. A comprehensive and dated phylogenomic analysis of butterflies. *Current Biology* **28**, 770-778.e5 (2018).
7. Wahlberg, N. & Wheat, C. W. Genomic outposts serve the phylogenomic pioneers: designing novel nuclear markers for genomic DNA extractions of Lepidoptera. *Systematic Biology* **57**, 231–242 (2008).
8. Toussaint, E. F. A. et al. Afrotropics on the wing: phylogenomics and historical biogeography of awl and policeman skippers. *Systematic Entomology* **46**, 172–185 (2021).
9. Ratnasingham, S. & Hebert, P. D. BOLD: The Barcode of Life Data System (http://www. barcodinglife. org). *Molecular Ecology Notes* **7**, 355–364 (2007).
10. Camacho, C. et al. BLAST+: architecture and applications. *BMC Bioinformatics* **10**, 1–9 (2009).
11. Cho, S. et al. Preserving and vouchering butterflies and moths for large-scale museum-based molecular research. *PeerJ* **4**, e2160 (2016).
12. Kawahara, A. Y. et al. Phylogenomics reveals the evolutionary timing and pattern of butterflies and moths. *Proceedings of the National Academy of Sciences* **116**, 22657–22663 (2019).
13. Regier, J. C. et al. A large-scale, higher-Level, molecular phylogenetic study of the insect order Lepidoptera (moths and butterflies). *PLOS ONE* **8**, 1–23 (2013).
14. Wahlberg, N., Wheat, C. W. & Peña, C. Timing and patterns in the taxonomic diversification of Lepidoptera (butterflies and moths). *PLOS ONE* **8**, e80875 (2013).
15. Mayer, C. et al. Adding leaves to the Lepidoptera tree: capturing hundreds of nuclear genes from old museum specimens. *Systematic Entomology* **46**, 649–671 (2021).

16. Rota, J. et al. The unresolved phylogenomic tree of butterflies and moths (Lepidoptera): Assessing the potential causes and consequences. *Systematic Entomology* **47**, 531–550 (2022).
17. Hamilton, C. A. et al. Phylogenomics resolves major relationships and reveals significant diversification rate shifts in the evolution of silk moths and relatives. *BMC Evolutionary Biology* **19**, 182–182 (2019).
18. Breinholt, J. W. et al. Resolving relationships among the megadiverse butterflies and moths with a novel pipeline for Anchored Phylogenomics. *Systematic Biology* **67**, 78–93 (2018).
19. Krueger, F. *Trim Galore Version 0.4.0*. (2015).
20. Zhan, S., Merlin, C., Boore, J. L. & Reppert, S. M. The monarch butterfly genome yields insights into long-distance migration. *Cell* **147**, 1171–1185 (2011).
21. Katoh, K. & Standley, D. M. MAFFT Multiple sequence alignment software version 7: improvements in performance and usability. *Molecular Biology and Evolution* **30**, 772–780 (2013).
22. Kück, P. & Meusemann, K. FASconCAT: Convenient handling of data matrices. *Molecular Phylogenetics and Evolution* **56**, 1115–1118 (2010).
23. Bazinet, A. L., Cummings, M. P., Mitter, K. T. & Mitter, C. W. Can RNA-Seq resolve the rapid radiation of advanced moths and butterflies (Hexapoda: Lepidoptera: Apoditrysia)? An exploratory study. *PLOS ONE* **8**, e82615 (2013).
24. Kawahara, A. Y. & Breinholt, J. W. Phylogenomics provides strong evidence for relationships of butterflies and moths. *Proceedings of the Royal Society B: Biological Sciences* **281**, 20140970–20140970 (2014).
25. Romiguier, J. et al. Comparative population genomics in animals uncovers the determinants of genetic diversity. *Nature* **515**, 261–263 (2014).
26. Zhan, S. et al. The genetics of monarch butterfly migration and warning colouration. *Nature* **514**, 317–321 (2014).
27. Cong, Q., Borek, D., Otwinowski, Z. & Grishin, N. V. Skipper genome sheds light on unique phenotypic traits and phylogeny. *BMC Genomics* **16**, 1–13 (2015).
28. Cong, Q. et al. Speciation in cloudless sulphurs gleaned from complete genomes. *Genome Biology and Evolution* **8**, 915–931 (2016).
29. Cong, Q. et al. Complete genomes of Hairstreak butterflies, their speciation and nucleo-

mitochondrial incongruence. *Scientific Reports* **6**, 1–15 (2016).
30. Pauli, T. et al. Transcriptomic data from panarthropods shed new light on the evolution of insulator binding proteins in insects. *BMC Genomics* **17**, 861 (2016).
31. Shen, J. et al. Complete genome of *Pieris rapae*, a resilient alien, a cabbage pest, and a source of anti-cancer proteins. *F1000Research* **5**, (2016).
32. Shen, J., Cong, Q., Borek, D., Otwinowski, Z. & V Grishin, N. Complete genome of *Achalarus lyciades*, the first representative of the Eudaminae subfamily of skippers. *Current Genomics* **18**, 366–374 (2017).
33. Allio, R. et al. Whole genome shotgun phylogenomics resolves the pattern and timing of swallowtail butterfly evolution. *Systematic Biology* **69**, 38–60 (2020).
34. Chaturvedi, S. et al. Recent hybrids recapitulate ancient hybrid outcomes. *bioRxiv* 769901 (2019) doi:10.1101/769901.
35. Edelman, N. B. et al. Genomic architecture and introgression shape a butterfly radiation. *Science* **366**, 594–599 (2019).
36. Li, W. et al. Genomes of skipper butterflies reveal extensive convergence of wing patterns. *Proceedings of the National Academy of Sciences* **116**, 6232–6237 (2019).
37. VanKuren, N. W., Massardo, D., Nallu, S. & Kronforst, M. R. Butterfly mimicry polymorphisms highlight phylogenetic limits of gene reuse in the evolution of diverse adaptations. *Molecular Biology and Evolution* **36**, 2842–2853 (2019).
38. Zhang, J., Shen, J., Cong, Q. & Grishin, N. V. Genomic analysis of the tribe Emesidini (Lepidoptera: Riodinidae). *Zootaxa* **4668**, 475–488 (2019).
39. Zhang, J., Cong, Q., Shen, J., Brockmann, E. & Grishin, N. V. Three new subfamilies of skipper butterflies (Lepidoptera, Hesperiidae). *ZooKeys* **861**, 91 (2019).
40. Zhang, J., Cong, Q., Shen, J., Brockmann, E. & Grishin, N. V. Genomes reveal drastic and recurrent phenotypic divergence in firetip skipper butterflies (Hesperiidae: Pyrrhopyginae). *Proceedings of the Royal Society B: Biological Sciences* **286**, 20190609 (2019).
41. Bankevich, A. et al. SPAdes: a new genome assembly algorithm and its applications to single-cell sequencing. *Journal of Computational Biology* **19**, 455–477 (2012).
42. Ellis, E. A., Storer, C. G. & Kawahara, A. Y. De novo genome assemblies of butterflies. *GigaScience* **10**, giab041 (2021).
43. Denton, J. F. et al. Extensive error in the number of genes inferred from draft genome

assemblies. *PLOS Computational Biology* **10**, e1003998 (2014).

44. Price, M. N., Dehal, P. S. & Arkin, A. P. FastTree 2 - Approximately maximum-likelihood trees for large alignments. *PLOS ONE* **5**, e9490–e9490 (2010).
45. Kocot, K. M., Citarella, M. R., Moroz, L. L. & Halanych, K. M. PhyloTreePruner: a phylogenetic tree-based approach for selection of orthologous sequences for phylogenomics. *Evolutionary Bioinformatics* **9**, 429–435 (2013).
46. Ranwez, V., Douzery, E. J., Cambon, C., Chantret, N. & Delsuc, F. MACSE v2: toolkit for the alignment of coding sequences accounting for frameshifts and stop codons. *Molecular Biology and Evolution* **35**, 2582–2584 (2018).
47. Capella-Gutiérrez, S., Silla-Martínez, J. M. & Gabaldón, T. trimAl: a tool for automated alignment trimming in large-scale phylogenetic analyses. *Bioinformatics* **25**, 1972–1973 (2009).
48. Larsson, A. AliView: a fast and lightweight alignment viewer and editor for large datasets. *Bioinformatics* **30**, 3276–3278 (2014).
49. Nylander, J. *catfasta2phyml*. (2018). Available at: https://github.com/nylander/catfasta2phyml
50. Oakley, T. H. et al. Osiris: accessible and reproducible phylogenetic and phylogenomic analyses within the Galaxy workflow management system. *BMC Bioinformatics* **15**, 1–9 (2014).
51. Schwery, O. & O'Meara, B. C. MonoPhy: a simple R package to find and visualize monophyly issues. *PeerJ Computer Science* **2**, e56 (2016).
52. Minh, B. Q. et al. IQ-TREE 2: New models and efficient methods for phylogenetic inference in the genomic era. *Molecular Biology and Evolution* **37**, 1530–1534 (2020).
53. Pease, J. B., Brown, J. W., Walker, J. F., Hinchliff, C. E. & Smith, S. A. Quartet Sampling distinguishes lack of support from conflicting support in the green plant tree of life. *American Journal of Botany* **105**, 385–403 (2018).
54. Regier, J. C. et al. Arthropod relationships revealed by phylogenomic analysis of nuclear protein-coding sequences. *Nature* **463**, 1079–1083 (2010).
55. Zwick, A. *Degeneracy Coding Web Service*. (2010). Available at: https://github.com/carlosp420/degenerate-dna
56. Lanfear, R., Frandsen, P. B., Wright, A. M., Senfeld, T. & Calcott, B. PartitionFinder 2:

new methods for selecting partitioned models of evolution for molecular and morphological phylogenetic analyses. *Molecular Biology and Evolution* **34**, 772–773 (2017).
57. Stamatakis, A. RAxML-VI-HPC: maximum likelihood-based phylogenetic analyses with thousands of taxa and mixed models. *Bioinformatics* **22**, 2688–2690 (2006).
58. Nguyen, L.-T., Schmidt, H. A., Von Haeseler, A. & Minh, B. Q. IQ-TREE: a fast and effective stochastic algorithm for estimating maximum-likelihood phylogenies. *Molecular Biology and Evolution* **32**, 268–274 (2015).
59. Minh, B. Q., Nguyen, M. A. T. & von Haeseler, A. Ultrafast approximation for phylogenetic Bootstrap. *Molecular Biology and Evolution* **30**, 1188–1195 (2013).
60. Hoang, D. T., Chernomor, O., von Haeseler, A., Minh, B. Q. & Vinh, L. S. UFBoot2: Improving the Ultrafast Bootstrap approximation. *Molecular Biology and Evolution* **35**, 518–522 (2018).
61. Guindon, S. et al. New algorithms and methods to estimate maximum-likelihood phylogenies: assessing the performance of PhyML 3.0. *Systematic Biology* **59**, 307–321 (2010).
62. Toussaint, E. F. A. et al. Anchored phylogenomics illuminates the skipper butterfly tree of life. *BMC Evolutionary Biology* **18**, 101 (2018).
63. Mendes, F. K. & Hahn, M. W. Why concatenation fails near the anomaly zone. *Systematic Biology* **67**, 158–169 (2018).
64. Maddison, W. P. Gene trees in species trees. *Systematic Biology* **46**, 523–536 (1997).
65. Edwards, S. V. Is a new and general theory of molecular systematics emerging? *Evolution: International Journal of Organic Evolution* **63**, 1–19 (2009).
66. Zhang, C., Rabiee, M., Sayyari, E. & Mirarab, S. ASTRAL-III: polynomial time species tree reconstruction from partially resolved gene trees. *BMC Bioinformatics* **19**, 15–30 (2018).
67. Sayyari, E. & Mirarab, S. Fast coalescent-based computation of local branch support from quartet frequencies. *Molecular Biology and Evolution* **33**, 1654–1668 (2016).
68. Strimmer, K. & von Haeseler, A. Likelihood-mapping: A simple method to visualize phylogenetic content of a sequence alignment. *Proceedings of the National Academy of Sciences* **94**, 6815 (1997).
69. Wahlberg, N. et al. Nymphalid butterflies diversify following near demise at the

Cretaceous/Tertiary boundary. *Proceedings of the Royal Society B: Biological Sciences* **276**, 4295–4302 (2009).

70. Wahlberg, N., Rota, J., Braby, M. F., Pierce, N. E. & Wheat, C. W. Revised systematics and higher classification of pierid butterflies (Lepidoptera: Pieridae) based on molecular data. *Zoologica Scripta* **43**, 641–650 (2014).
71. Ho, S. Y. & Jermiin, L. S. Tracing the decay of the historical signal in biological sequence data. *Systematic Biology* **53**, 623–637 (2004).
72. Jermiin, L. S., Ho, S. Y., Ababneh, F., Robinson, J. & Larkum, A. W. The biasing effect of compositional heterogeneity on phylogenetic estimates may be underestimated. *Systematic Biology* **53**, 638–643 (2004).
73. Misof, B. et al. Phylogenomics resolves the timing and pattern of insect evolution. *Science* **346**, 763–767 (2014).
74. Smith, S. A. & O'Meara, B. C. TreePL: divergence time estimation using penalized likelihood for large phylogenies. *Bioinformatics* **28**, 2689–2690 (2012).
75. Sun, M. et al. Recent accelerated diversification in rosids occurred outside the tropics. *Nature Communications* **11**, 1–12 (2020).
76. Zhang, L. et al. The water lily genome and the early evolution of flowering plants. *Nature* **577**, 79–84 (2020).
77. Louca, S. & Pennell, M. W. Extant timetrees are consistent with a myriad of diversification histories. *Nature* **580**, 502–505 (2020).
78. Helmstetter, A. J. et al. Pulled Diversification Rates, Lineages-Through-Time Plots and Modern Macroevolutionary Modelling. *bioRxiv* 2021.01.04.424672 (2021) doi:10.1101/2021.01.04.424672.
79. Morlon, H., Robin, S. & Hartig, F. Studying speciation and extinction dynamics from phylogenies: addressing identifiability issues. *Trends in Ecology & Evolution* **37**, 497-506 (2022).
80. de Jong, R. Fossil butterflies, calibration points and the molecular clock (Lepidoptera: Papilionoidea). *Zootaxa* **4270**, 1–63 (2017).
81. Helfrich, P., Rieb, E., Abrami, G., Lücking, A. & Mehler, A. TreeAnnotator: versatile visual annotation of hierarchical text relations. in *Proceedings of the Eleventh International Conference on Language Resources and Evaluation (LREC 2018)* (2018).

82. Rabosky, D. L. Automatic detection of key innovations, rate shifts, and diversity-dependence on phylogenetic trees. *PLOS ONE* **9**, e89543–e89543 (2014).
83. Rabosky, D. L. et al. BAMMtools: An R package for the analysis of evolutionary dynamics on phylogenetic trees. *Methods in Ecology and Evolution* **5**, 701–707 (2014).
84. Maddison, W. P. & Maddison, D. R. Mesquite: a modular system for evolutionary analysis. (2018).
85. Höhna, S. et al. A Bayesian approach for estimating branch-specific speciation and extinction rates. *bioRxiv* 555805 (2019) doi:10.1101/555805.
86. Höhna, S. et al. RevBayes: Bayesian phylogenetic inference using graphical models and an interactive model-specification language. *Systematic Biology* **65**, 726–736 (2016).
87. Moore, B. R., Höhna, S., May, M. R., Rannala, B. & Huelsenbeck, J. P. Critically evaluating the theory and performance of Bayesian analysis of macroevolutionary mixtures. *Proceedings of the National Academy of Sciences* **113**, 9569–9574 (2016).
88. Rabosky, D. L., Mitchell, J. S. & Chang, J. Is BAMM flawed? Theoretical and practical concerns in the analysis of multi-rate diversification models. *Systematic Biology* **66**, 477–498 (2017).
89. Robinson, G. S., Ackery, P. R., Kitching, I. J. & Beccaloni, G. W. HOSTS - a database of the World's lepidopteran hostplants. http://www.nhm.ac.uk/research-curation/projects/hostplants/ (2020).
90. Saito, M. U., Jinbo, U., Yago, M., Kurashima, O. & Ito, M. Larval host records of butterflies in Japan. *Ecological Research* **31**, 491–491 (2016).
91. Veenakumari, K., Mohanraj, P. & Sreekumar, P. Host plant utilization by butterfly larvae in the Andaman and Nicobar Islands (Indian Ocean). *Journal of Insect Conservation* **1**, 235–246 (1997).
92. Kunte, K. Additions to known larval host plants of Indian butterflies. *Journal of the Bombay Natural History Society* **103**, 119–122 (2006).
93. Kalesh, S. & Prakash, S. K. Additions to larval host plants of butterflies of the Western Ghats, Kerala, Southern India (Rhopalocera, Lepidoptera). Part 1. *Journal of the Bombay Natural History Society* **104**, 235–238 (2007).
94. Kalesh, S. & Prakash, S. K. Additions to larval host plants of butterflies of the Western Ghats, Kerala, Southern India (Rhopalocera, Lepidoptera). Part 2. *Journal of the Bombay*

Natural History Society **112**, 111–113 (2015).

95. Naik, D. & Mustak, M. S. Additions to larval host plants of Indian butterflies (Lepidoptera). *Journal of the Bombay Natural History Society* **112**, 181–183 (2015).

96. Karmakar, T. et al. Early stages and larval host plants of some northeastern Indian butterflies. *Journal of Threatened Taxa* **10**, 11780–11799 (2018).

97. Nitin, R. et al. Larval host plants of the butterflies of the Western Ghats, India. *Journal of Threatened Taxa* **10**, 11495–11550 (2018).

98. WFO. World Flora Online. http://www.worldfloraonline.org (2020).

99. Robinson, G. S. et al. *Hostplants of the moth and butterfly caterpillars of the Oriental Region.* (Southdene Sdn Bhd, 2001).

100. Williams, M. C. What do the larvae of *Alaena amazoula* (Boisduval, 1847) (Lepidoptera: Lycaenidae: Poritiinae) feed on? *The Lepidopterists' Society of Africa* **17**, 140–150 (2006).

101. Edger, P. P. et al. The butterfly plant arms-race escalated by gene and genome duplications. *Proceedings of the National Academy of Sciences* **112**, 8362 (2015).

102. Braga, M. P., Landis, M. J., Nylin, S., Janz, N. & Ronquist, F. Bayesian inference of ancestral host–parasite interactions under a phylogenetic model of host repertoire evolution. *Systematic Biology* **69**, 1149–1162 (2020).

103. Kembel, S. W. et al. Picante: R tools for integrating phylogenies and ecology. *Bioinformatics* **26**, 1463–1464 (2010).

104. Faith, D. P. Conservation evaluation and phylogenetic diversity. *Biological Conservation* **61**, 1–10 (1992).

105. Webb, C. O., Ackerly, D. D., McPeek, M. A. & Donoghue, M. J. Phylogenies and community ecology. *Annual Review of Ecology and Systematics* **33**, 475–505 (2002).

106. Jorge, L. R., Prado, P. I., Almeida-Neto, M. & Lewinsohn, T. M. An integrated framework to improve the concept of resource specialisation. *Ecology Letters* **17**, 1341–1350 (2014).

107. Smith, S. A. & Brown, J. W. Constructing a broadly inclusive seed plant phylogeny. *American Journal of Botany* **105**, 302–314 (2018).

108. Beckett, S. J. Improved community detection in weighted bipartite networks. *Royal Society Open Science* **3**, 140536 (2016).

109. Dormann, C. F., Gruber, B. & Fründ, J. Introducing the bipartite package: analysing ecological networks. *R News* **8**, 8–11 (2008).

110. R Core Team. R: A language and environment for statistical computing. (2009).

111. Revell, L. J. phytools: an R package for phylogenetic comparative biology (and other things). *Methods in Ecology and Evolution* **3**, 217–223 (2012).

112. Beaulieu, J. M. & O'Meara, B. C. Detecting hidden diversification shifts in models of trait-dependent speciation and extinction. *Systematic Biology* **65**, 583–601 (2016).

113. Beaulieu, J., O'Meara, B., Caetano, D., Boyko, J. & Vasconcelos, T. *Package 'hisse'*. (2021). Available at: https://cran.r-project.org/web/packages/hisse/index.html.

114. Futuyma, D. J. Food plant specialization and environmental predictability in Lepidoptera. *The American Naturalist* **110**, 285–292 (1976).

115. Udvardy, M. D. F. *A classification of the biogeographical provinces of the world. Morges (Switzerland): International Union of Conservation of Nature and Natural Resources. IUCN Occasional Paper no. 18*. (1975).

116. Savela, M. Lepidoptera and some other life forms. https://www.funet.fi/pub/sci/bio/life/intro.html (2021).

117. Beeravolu, C. R. & Condamine, F. L. An extended Maximum Likelihood inference of geographic range evolution by dispersal, local extinction and cladogenesis. *bioRxiv* 038695 (2016) doi:10.1101/038695.

118. Ree, R. H. & Smith, S. A. Maximum likelihood inference of geographic range evolution by dispersal, local extinction, and cladogenesis. *Systematic Biology* **57**, 4–14 (2008).

119. Matzke, N. J. BioGeoBEARS: biogeography with Bayesian (and likelihood) evolutionary analysis in R scripts. (2013).

120. Ree, R. H. Detecting the historical signature of key innovations using stochastic models of character evolution and cladogenesis. *Evolution* **59**, 257–265 (2005).

121. Ronquist, F. Dispersal-vicariance analysis: a new approach to the quantification of historical biogeography. *Systematic Biology* **46**, 195–203 (1997).

122. Burnham, K. P. & Anderson, D. R. Practical use of the information-theoretic approach. in *Model selection and inference* (eds. Burnham, K. P. & Anderson, D. R.) 75–117 (Springer, 1998).

123. Li, X. et al. A diversification relay race from Caribbean-Mesoamerica to the Andes: historical biogeography of *Xylophanes* hawkmoths. *Proceedings of the Royal Society B: Biological Sciences* **289**, 20212435 (2022).

Supplementary Text

A Global Phylogeny of Butterflies Reveals Their Evolutionary History, Ancestral Hosts, and Biogeographic Origins

Nature Ecology and Evolution

Additional Information on Results

Contents:

1. Datasets, partitioning and model selection
2. Tree inference and branch support
3. Coalescent-based methods
4. Topology tests
5. Divergence time estimation
6. Diversification analyses
7. Larval hosts
8. Coding and curation of distributional data
9. Biogeography
10. Ancestral state estimation: Plants
11. Butterfly speciation rate in relation to angiosperms

Supplementary Text:

1. Datasets, partitioning and model selection

In total, 391 loci with more than 60% coverage among the 2,258 samples were selected for inclusion in subsequent analyses. Of the 391 loci, nucleotide lengths varied from 138 bp to 1,854 bp, with an average of 412 bp; taxon coverage by locus varied from 1,382 taxa to 2,141 taxa, with an average of 1,993 taxa. Three concatenated datasets were generated for phylogenetic analyses: 1) nt123, nucleotide, all codon positions: 161,166 nucleotides; 2) degen, nucleotide, degenerated to exclude all synonymous signal: 161,166 nucleotides; and 3) aa, amino acids: 53,722 amino acid residues. For analyses initially partitioned by locus, 321, 359, and 154 meta-partitions were generated for nt123, degen, and aa datasets, respectively. Detailed partitioning schemes and the best models for each partition are shown in Table S33.

2. Tree inference and branch support

ML trees from nine phylogenetic analyses with different datasets and partitioning schemes indicate that all butterfly families, 38 out of 41 (92.7%) subfamilies, and 96 out of 132 (72.7%) tribes are monophyletic (see Table S2 for support values). Since some family-group taxa are represented in our trees by only one tip, these taxa could not be assessed for monophyly, but are herein assumed to be monophyletic as per the existing taxonomic hypotheses in previous published studies. The full tree with estimated divergences for all nodes for the tree shown in Fig. 1 can be found in Fig. S1.

Family-level relationships were consistent among the nine analyses presented in Table S2. Papilionidae was recovered as sister to the other butterfly families, which were divided into two clades consistent with previous studies (e.g., Espeland et al.[1]): Hedylidae + Hesperiidae, and Pieridae + (Nymphalidae + (Lycaenidae + Riodinidae)). Subfamily-level relationships were consistent among the degen and aa analyses, except for relationships between the four hesperiid subfamilies comprising the sister clade of Heteropterinae. The degen359 analysis recovered Malazinae + (Hesperiinae + (Barcinae + Trapezitinae)), whereas the other degen and aa analyses supported Hesperiinae + (Trapezitinae + (Malazinae + Barcinae)). Furthermore, in the nt123 ML analyses a third topological relationship was recovered: (Hesperiinae + (Malazinae + (Barcinae + Trapezitinae))). Some of the relevant hesperiid nodes in these trees have only moderate support (e.g., with high SH-aLRT values but low UFBS values; Data S21), thus precise relationships between these subfamilies remain uncertain.

The nt123 ML analyses recovered additional subfamily-level relationships not found in other ML analyses: 1) The placement of lycaenid subfamily Miletinae as sister to Aphnaeinae + Poritiinae in nt123 trees, but the sister-group to a five-subfamily clade in degen and aa trees; 2) The nymphalid subfamily Libytheinae as sister to all other Nymphalidae in nt123, but instead forming a sister-group relationship with Danainae in degen and aa trees (Data S21).

It is well-known that inclusion of synonymous changes can result in compositional bias, leading to incorrect phylogenetic relationships, which is especially true for deep divergences[2,3]. Therefore, our discussion below focuses on the degen359 and aa154 analyses.

Within Papilionidae, there was no evidence for non-monophyly in any of the eight tribes, and all degen and aa analyses resulted in trees supporting the monophyly of Papilioninae with strong support (Table S2). Baroniinae (Baroniini) is the earliest branching lineage and is the sister-group to the Papilioninae + Parnassiinae in all analyses. Within Hesperiidae, there was no evidence for non-monophyly in any of the 13 subfamilies. Some hesperiine tribal clades were found to contain genera not yet formally assigned to a tribe (e.g., Aeromachini, which contains the *incertae sedis* genera *Creteus*, *Lepella* and *Prosopalpus*). We consider these tribes monophyletic until future taxonomic work can be conducted to confirm whether these genera should be formally transferred to their corresponding tribal clades. However, 8 out of 29 tribes were found to be non-monophyletic, often due to the placement of a single genus or species: 1) Oileidini, due to *Marela tamyroides* being nested in Phocidini instead of being sister to its congener, *M. tamyris*; 2) Phocidini, which is consequently paraphyletic with respect to *M. tamyroides*; 3) Celaenorrhinini and 4) Tagiadini, which are both polyphyletic due to the presence of the tagiadine genus *Kobelana* in Celaenorrhinini and the celaenorrhinine genus *Triskelionia* in Tagiadini; 5) Achlyodini, due to one of the five sampled species of *Eantis* (*E. platypterus*) recovered in Carcharodini rather than Achlyodini; 6) Carcharodini, which is consequently paraphyletic with respect to *E. platypterus*; 7) Hesperiini, which is polyphyletic due to *Carystoides* being sister to Megathymini instead of in the clade with all other Hesperiini; and 8) Erionotini, which is polyphyletic with strong support in the degen359 tree (UFBS/SH-aLRT support values = 98/100) and with moderate support in the aa154 tree (75/100). Erionotini is split into two clades, neither of which have strong support (Data S21), thus exact relationships within this tribe are still unclear. Inter-tribal relationships in Hesperiidae are mostly consistent between the aa and degen topologies, except in Pyrginae: Erynnini is moderately supported as sister to all

other Pyrginae in the aa154 tree (UFBS = 97, but SH-aLRT = 45.0), whereas in the degen359 tree, Achlyodini is moderately supported as sister to all other Pyrginae (99/74.6). Within Pieridae, monophyly of all four subfamilies was recovered. Pierinae is the only pierid subfamily divided into tribes, and there was no evidence of non-monophyly in any of those six tribes. However, some inter-tribal relationships differ between the degen and aa analyses: Nepheroniini was sister to all other Pierinae in the aa154 tree, but with moderate/weak support (95/42.8), whereas Anthocharidini was moderately supported as sister to the other Pierinae tribes in the degen359 tree (100/71.4). Within Riodinidae, monophyly of both subfamilies was recovered in all analyses, and only two out of 12 tribes were found to have evidence for non-monophyly: Calydnini and Sertaniini. A monophyletic Sertaniini was recovered in all analyses except the aa154 analysis, in which *Sertania* was sister to Riodinini and *Xanthosa* was sister to genus *Echydna*, resulting in non-monophyly of tribe Calydnini. Additionally, bootstrap support for Sertaniini was < 95% in all three degen trees (Table S2).

Within Lycaenidae, monophyly of five out of seven described subfamilies was recovered, and evidence for non-monophyly of 14 out of 33 tribes was found. Polyommatinae and Theclinae form a strongly supported clade (100/100 in the aa154 analysis), but both subfamilies are polyphyletic with respect to each other. The thecline tribe Hypotheclini is sister to the polyommatine genus *Cupidopsis*, and the Polyommatinae + Hypotheclini clade is in turn nested within the rest of Theclinae, sister to a multi-tribe clade that includes Hypolycaenini. Non-monophyletic tribes include: 1) Liptenini (Poritiinae), which comprises three separate clades, one of which also contained the type genus of Epitolini; 2) Epitolini (Poritiinae), in which *Epitolina* is in a subclade of Liptenini, separate from the clade containing all other Epitolini as also indicated by Libert[4]; 3) Lycaenini and 4) Heliophorini, the two tribes of Lycaeninae, which form a polyphyletic assemblage due to the lycaenine genus *Hyrcana* being more closely related to *Heliophorus* than to *Lycaena*; 5) Zesiusini, with none of the three sampled genera recovered as sister to each other (supporting the results of Espeland et al.[1]); 6) Luciini, which forms two clades, one of which (the one containing type genus *Lucia*) is more closely related to tribe Ogyrini; 7) Arhopalini, which contains four sampled genera that form a separate clade with the Theclini genus *Amblopala*; 8) Theclini, in which the aforementioned *Amblopala* is not part of the clade containing all other Theclini; 9) Deudorigini, which is polyphyletic in some analyses, but paraphyletic in others with moderate support (Table S2) with respect to the loxurine *Drina*

maneia; 10) Loxurini, which has three genera (*Drina, Neomyrina,* and *Thamala*) recovered as more closely related to other tribes than to the type genus *Loxura*; 11) Amblypodiini, in which genera *Iraota* and *Myrina* are sister to Iolaini instead of type genus *Amblypodia*; 12) Cheritrini, in which *Dapidodigma* is sister to the type genus of Zesiusini instead of being in the clade containing all other Cheritrini; 13) Hypolycaenini, in which *Hemiolaus* is more closely related to Oxylidini than to the other Hypolycaenini; and 14) Polyommatini, in which genus *Cupidopsis* is sister to the thecline tribe Hypotheclini. The clade containing all other Polyommatini is sister to Niphandini, in accordance with the topology found in Espeland et al.[1].

Within Nymphalidae, monophyly of 11 out of 12 subfamilies was confirmed; the twelfth subfamily, Calinaginae, was only represented by a single species. Evidence of non-monophyly in 12 out of 44 tribes was recovered: 1) Neptini, due to *Lebadea* supported as appearing in a separate clade, sister to Pseudacraeini (aa154; 89/99.8); 2) Limenitidini, due to *Kumothales* appearing in a separate clade, sister to Cymothoini; 3) Vagrantini, which is paraphyletic with respect to Argynnini; 4) Epicaliini, which has species in three separate clades within Biblidinae; 5) Epiphilini, due to *Lucinia* appearing in a separate clade, sister to Callicorini; 7) Coeini, a polyphyletic tribe of Nymphalinae, with *Historis* and *Pycina* being successive sister groups to the other Coeini and the other nymphaline tribes; 8) Kallimini which has species in five separate clades within Nymphalinae (e.g., *Hypolimnas* not sister to Kallimini *sensu stricto*); 9) Junoniini, which is paraphyletic with respect to the kallimine genus *Hypolimnas*; 10) Charaxini, which is paraphyletic with respect to Euxanthini; 11) Prothoini, which is represented in by the genera *Agatasa* and *Prothoe*; the former is sister to Pallaini and the latter is sister to Charaxini + Euxanthini; and 12) Amathusiini, due to *Hyantis* + *Morphopsis* forming a separate clade that is sister to Melanitini + Dirini in the aa154 tree (97/99.9), although *Hyantis* + *Morphopsis* is weakly supported as sister to Zetherini in the degen359 tree (44/78.7). There is another inter-tribal relationship that differs between the aa154 and degen359 trees, with moderate or weak support: Anaeomorphini is sister to all other Charaxinae in aa154 (69/99), whereas it is sister to just Anaeini in degen359 (93/75.1) with Preponini instead being sister to all other Charaxinae (90/95.6). Many of these findings are in agreement with results of previous nymphalid phylogenetic work (e.g., Wahlberg et al.[5], Dhungel and Wahlberg[6], Chazot et al.[7]), and were independently corroborated by a recent study published after our analyses had been completed[8,9].

We compared branch support values from the main analysis in our study (aa154) to those of published butterfly phylogenetic studies. Table S46 shows support values for family- and subfamily-level nodes of the aa154 tree, compared with other studies. In general, our tree recovered relationships with higher support values. Clades with low support or considered non-monophyletic previously, like Coliadinae and Pseudopontiinae[7], were recovered with strong support (100/100).

Quartet sampling strongly supported the superfamily and family-level clades recovered in the ML analyses of the degen359 and aa154 analyses, with almost all corresponding nodes having high quartet concordance scores (QC > 0.2; degen: Data S15 and Table S39; aa: Data S16 and Table S40). One exception was the sister-group relationship of Hedylidae + Hesperiidae, which received a moderate QC score (0.2 ≥ QC > 0).

Four areas of potential topological conflict at the subfamilial and tribal levels (see hypotheses in methods above) were also assessed with quartet sampling. 1) *Position of Libytheinae*. Both trees support a sister relationship between Libytheinae and Danainae, instead of an alternate relationship where Libytheinae is sister to all other Nymphalidae. The quartet sampling scores indicate only moderate preference for this relationship, though they are not skewed to the alternate topology (degen359, node 1575: QC = 0.16, QD = 0.89, QI = 0.95; aa154, node 1002: QC = 0.15, QD = 0.97, QI = 0.91). 2) *Placement of the hesperiid subfamilies Barcinae and Malazinae*. The placement of these subfamilies, relative to each other and to subfamilies Hesperiinae and Trapezitinae, is not fully resolved. The monophyly of Barcinae + Malazinae + Hesperiinae + Trapezitinae is strongly supported by the degen359 tree but only moderately supported by the aa154 tree, and the quartet sampling scores indicate persistence of one discordant topology (degen359, node 20: QC = 0.31, QD = 0.83, QI = 0.90; aa154, node 468: QC = 0.18, QD = 0.28, QI = 0.95). Within this clade, the degen359 supports a sister relationship between Barcinae and Trapezitinae (degen359, node 374: QC = 0.61, QD = 0.55, QI = 0.93), but for the other two subfamilies there is a moderate preference for discordant quartets (degen359, node 21: QC = -0.03, QD = 0.98, QI = 0.75) instead of the topology actually recovered in the degen359 tree (i.e., Malazinae sister to all three subfamilies). Quartet sampling scores for the aa154 tree indicate moderate support for a different topology in which Malazinae is sister to Barcinae (aa154, node494: QC = 0.05, QD = 0.67, QI = 0.81) and strong support for Trapezitinae as the sister group to Barcinae + Malazinae (aa154, node 469: QC = 0.43, QD =

0.71, QI = 0.87) instead of being sister to just Barcinae. 3) *Monophyly of Papilioninae*. Quartet sampling scores demonstrated strong preference for the monophyly of Papilioninae, and no skew towards a discordant topology wherein tribe Leptocircini is polyphyletic with respect to the other Papilioninae tribes (degen359, node 801: QC = 0.1, QD = 0.62, QI = 0.94; aa154, node 35: QC = 0.25, QD = 0.86, QI = 0.92). 4) *Tribal relationships in Pierinae*. The degen359 tree recovered Anthocharidini as sister to all other Pierinae, while the aa154 recovered Nepheroniini as sister to all other Pierinae. In both trees, quartet sampling scores indicated strong preference for discordant quartets but were not skewed towards either of the discordant topologies (degen359, node 883: QC = -0.20, QD = 0.89, QI = 0.94; aa154, node 889: QC = -0.19, QD = 0.23, QI = 0.88).

3. Coalescent-based methods

Single gene ML trees from the nt123 dataset were imported into ASTRAL to generate a species tree. The ASTRAL species tree shows strong support for 134 of the 152 named family-group clades (LPP > 0.95; Table S2), which is similar to the aa154 ML tree in the number of nodes that were well-supported (137/152 clades with strong support; UFBS > 95%, SH-aLRT > 80%). However, the topology has one significant difference: Hesperiidae + Hedylidae is sister to the remaining Papilionoidea. We do not consider this a valid result, as many unrelated studies have shown that Papilionidae is the sister-group to all remaining butterflies[1,7,10–12]. The topology with Hesperiidae + Hedylidae as the sister-group to the remainder of Papilionoidea only had moderate support in the ASTRAL species tree (LPP = 0.93). The alternative topology (Papilionidae as the sister-group to the remainder of butterfly families) had 100% UFBS support and >98% SH-aLRT support in the nt123, amino acid, and degen trees.

4. Topology tests

Results of the FcLM analyses for all four hypotheses in both degen and aa datasets are presented as triangle plots (Figs. S14-S15) and relevant quartet percentages are displayed in Table S3. Analyses used to test Hypothesis 1 (placement of Libytheinae) favor Libytheinae as sister to Danainae, with a plurality of quartets corresponding to topology T1 in both non-permuted datasets. This is compatible with ML tree reconstructions and shows that this result is robust against taxon sampling. Permutation analyses showed that the presence of heterogeneous

site composition, non-stationary substitution processes, or non-random distribution of missing data do not confound phylogenetic signal.

Analyses used to test Hypothesis 2 (placement of Barcinae and Malazinae) yielded contradictory results. The non-permuted aa analysis moderately supported a monophyletic Barcinae + Malazinae, with a slight plurality of quartets corresponding to topology T1 (Fig. S14), but the non-permuted degen analysis strongly supported a monophyletic Malazinae + Trapezitinae, with a majority of quartets corresponding to topology T2 (Fig. S15). Barcinae + Malazinae was recovered in the aa154 tree, but neither of these results corroborate the degen359 tree, which supported a monophyletic Barcinae + Trapezitinae (topology T3); this shows that the ML tree reconstructions are sensitive to taxon sampling. The permutation analyses show that confounding signal derived from heterogeneous site composition and non-random distribution of missing data is present in the degen dataset, and confounding signal derived from non-stationary substitution processes is present in the aa dataset.

Analyses testing Hypothesis 3 (Papilioninae) favor monophyly of Papilioninae, with a plurality of quartets corresponding to topology T1 in both non-permuted datasets (Figs. S14, S15). This is compatible with the ML tree supporting Leptocircini sister to other Papilioninae tribes and shows that the ML tree is robust against taxon sampling. Results of the permutation analyses show that the presence of heterogeneous site composition, non-stationary substitution processes or non-random distribution of missing data do not confound phylogenetic signal.

Analyses testing Hypothesis 4 (tribes of Pierinae) yielded contradictory results. The non-permuted degen analysis moderately supports a monophyletic Teracolini + outgroup (i.e., Anthocharidini is sister to Teracolini and all other Pierinae tribes except Nepheroniini), with a plurality of quartets corresponding to topology T1 (Fig. S15). This is compatible with the results of the degen359 tree and shows that the tree is robust against taxon sampling. However, the non-permuted aa dataset supports a monophyletic Nepheroniini + Teracolini, with a majority of quartets corresponding to topology T3 (Fig. S14). This is incompatible with the degen and aa154 ML trees, in which Nepheroniini is sister to remaining Pierinae. This demonstrates that the aa154 tree is sensitive to taxon sampling. Permutation analyses show that the degen dataset has confounding signal derived from heterogeneous site composition and non-random distribution of missing data and the aa dataset has confounding signal derived from non-stationary substitution processes.

5. Divergence time estimation

As expected, divergence time analyses that utilized secondary calibrations of butterfly clades from Kawahara et al. [10] yielded age estimates younger than the other dating analyses, which used only fossil calibrations and a single secondary calibration based on the age of angiosperms (Data S22, Table S4). The secondary calibration analyses yielded Cretaceous-era crown-group median age estimates for most butterfly families (except Hedylidae), which is in line with other independent butterfly studies (e.g., Chazot et al.[7]).

The divergence time estimation analysis based on the aa tree and only secondary calibrations was conducted twice with two different strategies of running TreePL. Both strategies yielded similar median age estimates; however, Strategy B (based on the Zhang et al.[13] protocol) often yielded significantly larger age ranges. For example, Papilionoidea had an age range of 12.2 Ma with Strategy B (108.2-96.0 Ma) compared to the 2.5 Ma age range (102.5-100.0 Ma) of Strategy A (Table S4). Both age ranges are summaries of 100+ iterations of TreePL, as opposed to true credibility intervals derived from a Bayesian approach. The dated tree presented in Fig. 1 uses age ranges calculated with Strategy A, but we include corresponding age ranges from Strategy B in Table S4 in order to provide more conservative alternatives.

6. Diversification analyses

Posterior probabilities for BAMM analyses converged at *ca*. 30 shifts (Fig. S21) (expectedNumberOfShifts = 30; lambdaInitPrior = ~2.89; lambdaShiftPrior = ~0.01; muInitPrior = ~2.89). Diversification patterns (speciation and extinction) and speciation rate over time were similar regardless of expected shift priors (see analyses with 20, 30 and 40 shifts, Fig. S17). Speciation rates increased steadily (Fig. S17) with a small shift to higher rates around 35 Ma. Tip net diversification rates can be found in Table S47 (see also Data S23).

There were several major diversification shifts across the evolution of butterflies, and some of the most diverse clades with shifts were found in Eudaminae, Hesperiinae, Poritiinae, Satyrinae, and Theclinae (Figs. 1, S19). The highest rate shift probability and lambda mean was detected in the clade of the MRCA of *Austrozephyrus* and *Chrysozephyrus* (Fig. 1, Table S44).

The RevBayes analysis was less sensitive but its results (Data S24) were largely congruent with that of BAMM, especially as both showed increased diversification rates in

clades within Eudaminae, Hesperiinae, and Satyrinae (Fig. S20). Differences between these two analyses may be because these approaches model rate shifts differently[14].

7. Larval hosts

While the vast majority of butterfly larvae feed on living tissues of angiosperms, there have been frequent deviations from this norm. For example, some larvae in the families Riodinidae and Lycaenidae (e.g., all species in the subfamily Miletinae) consume ant larvae, ant regurgitations, or Hemiptera[15,16]. Species in the wholly African tribes Liptenini and Pentilini appear to feed entirely on lichens or cyanobacteria. Feeding on spike mosses (Selaginellaceae) is found in all Oriental Ragadiini and in the Neotropical genus *Euptychia* (Euptychiina) (both Nymphalidae: Satyrinae)[17,18]. Some butterfly lineages also feed on gymnosperms. Palearctic *Chalinga* spp. (Nymphalidae: Limenitidinae) and Nearctic *Neophasia* spp. (Pieridae: Pierinae) are both specialized on coniferous trees, while Neotropical *Eumaeus* (Lycaenidae: Theclinae) and Oriental-Australian *Taenaris* spp. (Nymphalidae: Satyrinae) and *Luthrodes* spp. (Lycaenidae: Polyommatinae) consume Cycadaceae. The final dataset included data on 1,354 butterfly species that were sampled in our phylogeny and recorded 2,384 butterfly species-host family interactions summarized from over 31,000 literature records and > 10,000 additional rearing records from Costa Rica (Table S9).

The 1,336 herbivorous butterfly species sampled here feed on an average of 1.7 plant families per species and 68.1% of them are monophagous (feeding in one host family). Among polyphagous species, *Emesis mandana* (Riodinidae) can feed on 22 different host families, making its diet the most diverse one reported in this study. At the family level, Lycaenidae and Riodinidae have the highest host richness based on the number and PD of host families, respectively (Fig. S21, Table S13). The average number of host families is 2.11 in Lycaenidae and 2.06 in Riodinidae; and the average PD is 419.3 in Lycaenidae and 443.4 in Riodinidae. On the other hand, Hesperiidae have the narrowest diet compared to other families with a mean host family number of 1.35 and mean PD of 362.6. To quantify host divergence, we used four measurements, including MPD, MNTD, and two DSI metrics. Compared to PD, these divergence metrics measure the average pairwise distance between hosts (MPD and MNTD) and compare it to a randomly generated null distribution (DSI metrics). Results are highly consistent when using different measurements and we found Papilionidae to have the highest average host

divergence (mean MPD = 187.5, mean MNTD = 185.0, mean DSI_{MPD} = -0.96, mean DSI_{MNTD} = -1.03). Within each butterfly family, host divergence is often bimodally distributed, which is caused by the difference between monophagous and polyphagous species. When excluding monophagous species, Papilionidae still have the most divergent host range with a mean DSI_{MPD} of -0.15 and mean DSI_{MNTD} of -0.18. Here, the negative values of DSI_{MPD} and DSI_{MNTD} suggest that hosts are phylogenetically clustered (DSI < 0) rather than randomly distributed (DSI 0). However, exceptions, though uncommon, are found in several species. There are 14 species demonstrating both high host richness (> 5 families) and high divergence (MPD *p*–value > 0.05 and MNTD *p*–value > 0.05). Lycaenidae are overrepresented in these generalists with eight species included in the list. The wide breadth of host range is well known in some species such as *Hypolycaena phorbas* (Lycaenidae), *Zesius chrysomallus* (Lycaenidae), and *Pterourus glaucus* (Papilionidae), but reported for the first time in others. For example, *Shirozua jonasi* (Lycaenidae) can feed on 20 diverse plants including conifers (Pinaceae, Cupressaceae), grasses (Poaceae), legumes (Fabaceae), and plants with toxic secondary compounds (Anacardiaceae, Caprifoliaceae).

8. Coding and curation of distributional data

A total of 15,764 country records from the Pinkert et al.[19] dataset were curated, checked for accuracy, and assembled for the 2,244 butterfly species in our dataset (Table S5). These records represent 216 countries and major country subdivisions (i.e., 216 areas with their own ISO 3166-1 alpha-3 country code). These data were used to generate country-level species richness estimates (Table S27) which were used for tropicality analyses to obtain mean net diversification rates for each country (by summing individual net diversification rates for each species found in a country (Table S47) and dividing by that country's estimated species richness).

9. Biogeography

The 14-bioregion DECX analysis with *Baronia* (Analysis I, Table S6) estimated that the spatial late-Cretaceous origin of butterflies is in the Northern Hemisphere, specifically in both the Western Nearctic and Central American bioregions, indicating a possible Laurasian origin as opposed to a Gondwanan origin. The BioGeoBEARS DEC reconstructions using a 7-area coding

scheme show the ancestral range of butterflies as a combination of Nearctic + Neotropics using datasets with *Baronia* (pp = 0.69; Analysis IV, Table S6, see Table S48 and Fig. S9 for results for all nodes) and without *Baronia* (pp = 0.72; Analysis VI, Table S6, see Table S49 and Fig. S22 for results for all nodes). We found a similar result in our BioGeoBEARS DivaLIKE reconstruction (pp = 0.73; Analysis V, Table S6, see Table S50 and Fig. S9 for results for all nodes). Results from the DEC reconstruction were used to generate maps summarizing ancestral ranges at different points in the evolutionary history of butterflies (Fig. 3, Table S7). It thus appears that the Americas were very important in the early diversification of butterflies. There were subsequently massive dispersal waves into the Eastern Hemisphere via the Bering Land Bridge in multiple butterfly families. Overall, it seems that there were more dispersal events than vicariance events, which is not surprising considering the known dispersal abilities of extant butterflies. In the 14-bioregion DECX analysis, the ancestral distribution of family Hedylidae indicated a wider distribution than in the present day. Extant Hedylidae are known only from Central and South America[20], but results indicate that the MRCA of Hedylidae was also found in the West Indies.

Of the four models tested on the GeoHiSSE analyses of diversification associated with tropicality, Model #4 (“standard” GeoHiSSE model with one hidden state and no range-dependent diversification) was found to have the best fit (Table S45). Using this model, the net diversification of butterflies in tropical regions was found to be significantly greater than that of temperate butterflies (Data S1). There appeared to be many independent increases in diversification within most butterfly families; many of these increases coincided with transitions between the temperate and tropical states, represented in GeoHiSSE by an intermediate ‘widespread’ state (Data S1, S25). Locations and the quantity of these shifts noticeably differ across the three GeoHiSSE analyses (50/50, 30/70, pure temperate; see Extended Methods section 20), suggesting that the relatively broad, binary categorizations used to classify butterfly tropicality have significant impacts on the results, depending on the % tropicality value used as the ‘threshold’ between temperate and tropical.

Phenotypic rate analyses were conducted in BAMM to infer the number of shifts from temperate to tropical distribution within butterflies. The BAMM analysis with a prior of 25 expected trait rate shifts had a logL ESS value of 7.76; this was the highest value recovered across all six analyses (see Extended Methods section 20) though it is quite low for an ESS,

likely due to the large number of tips in the tree. Extending the search or conducting additional similar analyses in parallel did not improve ESS values. Phenotype (beta) rate analysis results for tropicality are shown in Table S51 and summarized in Table S44.

10. Ancestral state estimation: Plants

The butterfly-plant network was composed of 2,384 interactions between 1,354 butterfly species and 203 host taxa. Each butterfly and host taxon was assigned to one of 13 modules identified by the modularity analysis (Fig. S23). Modules with the largest number of butterfly species were modules 7 (190 species) and 8 (295 butterfly species) (Table S32). These are the modules containing the Fabaceae and Poaceae, respectively (Table S33). Butterfly richness of the remaining modules varied between 51 and 116 species. Using the network modules to group host taxa, the original network was reduced to 1,959 interactions between the 1,354 butterfly species and the 13 host modules. To account for the butterfly species in the tree (Fig. 1) that had been pruned prior to running the modularity analysis (due to absence of host record data), a SIMMAP ancestral state reconstruction was used to estimate host module assignments for these taxa with missing data. The ASR of our 13 plant modules suggests that feeding on the Fabaceae module is the ancestral condition of butterflies (pp = 0.7) (Fig. S24, Table S11; see Fig. S9 for node labels). While the ancestral feeding module of the family Papilionidae remains uncertain, the ancestral condition was more discernible for the other butterfly families: Module 13 (Malvaceae) for Hedylidae (pp=0.9) and module 7 (Fabaceae) for Hesperiidae (pp=0.8), Pieridae (pp=0.6), Nymphalidae (pp=0.6), Riodinidae (pp=0.8), and Lycaenidae (pp=0.9). We detected two major shifts in feeding to the module 8 (Poaceae) within Hesperiidae (pp=0.5) and Satyrinae (pp=0.9).

Because the Fabaceae module contained multiple families of plants, we tested whether Fabaceae was the most likely host family of the butterfly ancestor. To do so, we measured how strongly each host family contributes to each module by calculating a z-score. We compared the number of within-module interactions of each host to the mean of all hosts within its module. The Fabaceae module (Module 7) had Fabaceae as the host family that contributed most significantly, with a z-score of 2.426. The second highest z-score for the Fabaceae module was Fagaceae, with a z-score of 0.03410045 (Table S10). Fabaceae's $z = 2.426$ means that the number of interactions (= the number of butterflies) that Fabaceae has within module 7 is

2.42637848 standard deviations higher than the average number of interactions that the 8 hosts in module 7 have. All the other hosts have a *z*-score close to zero (about the same number of interactions as the average) or negative (less interactions than the average). The highest *z*-scores for the 12 other modules were: Module 1 (Annonaceae): 2.433; Module 2 (Rutaceae): 3.656; Module 3 (Arecaceae): 3.397; Module 4 (Lamiaceae): 3.024; Module 5 (Capparaceae): 2.991; Module 6 (Euphorbiaceae and Solanaceae): 1.581; Module 8 (Poaceae): 2.943; Module 9 (Combretaceae): 2.944; Module 10 (Passifloraceae): 3.213; Module 11 (Cannabaceae): 2.637; Module 12 (Salicaceae): 3.011; Module 13 (Malvaceae): 2.35 (Table S10).

SIMMAP ancestral state reconstruction for feeding strategy (specialist versus generalist) shows multiple shifts between traits (Fig. S25), with the ancestral condition for butterflies being the specialist state (pp = 0.708). The ancestral condition of each of the 7 butterfly families was also specialist (for complete probabilities see Table S52 and refer to Fig. S9 for node labels). The common ancestors of all butterfly families except Hedylidae were likely specialists on plants in the Fabaceae module. It is unclear what plants the hedylid common ancestor specialized on, with no module having a significant plurality in the ASR (Fig. S24)

Finally, through a comparison of alternative feeding behaviors (plant, lichen, Hemiptera, Hymenoptera), it was found that non-plant-based feeding behavior evolved independently within two subfamilies of Lycaenidae: Miletinae and a subclade of Poritiinae (Fig. S10, Table S12). Members of the Miletinae feed on ants or hemipterans, whereas the Poritiinae tribes Liptenini and Pentilini feed on lichens. There were additionally more recent shifts, mainly to ant-feeding, at tips in other Lycaenidae subfamilies.

11. Butterfly speciation rate in relation to angiosperms

Eight of the 18 HiSSE analyses examining whether diversification rates vary between different plant categories were inconclusive (ΔAIC < 2) (Data S26, Table S23). Further, none of the remaining 10 analyses (ΔAIC $\geq$ 2) indicate a link between host use and diversification rates (Fig. S26), providing no indication that use of a specific plant group, module, or generalist/specialist feeding behavior are linked to butterflies as a whole or within the specific families tested (i.e., best fit models were HiSSE models in all cases but one). The one BiSSE-like result was that of specialist vs. generalist in Pieridae, in which we found the best model to be a BiSSE-like model that does not allow for different turnover or epsilon rates for generalist or

specialist, with identical transition rates between the two states. While none of the best models suggest a link between feeding behavior and diversity without the influence of a hidden state, we found that in both Papilionoidea and Hesperiidae, speciation rates were lower in specialists (Data S27-S28).

References

1. Espeland, M. et al. A comprehensive and dated phylogenomic analysis of butterflies. *Current Biology* **28**, 770-778.e5 (2018).
2. Regier, J. C. et al. Arthropod relationships revealed by phylogenomic analysis of nuclear protein-coding sequences. *Nature* **463**, 1079–1083 (2010).
3. Zwick, A., Regier, J. C. & Zwickl, D. J. Resolving discrepancy between nucleotides and amino acids in deep-level arthropod phylogenomics: differentiating serine codons in 21-amino-acid models. *PLOS ONE* **7**, 1–12 (2012).
4. Libert, M. *Epitola l.s. Mise à jour de la révision (Lepidoptera, Lycaenidae)*. (Publié par l’auteur, 2020).
5. Wahlberg, N., Maresova, J., Murillo-Ramos, L., Collins, S. & Wu, L.-W. The phylogenetic positions of *Bhagadatta* Moore, 1898, *Kumothales* Overlaet, 1940 and *Harmilla* Aurivillius, 1892 (Lepidoptera, Nymphalidae, Limenitidinae) based on molecular data. *Nota Lepidopterologica* **43**, 167–171 (2020).
6. Dhungel, B. & Wahlberg, N. Molecular systematics of the subfamily Limenitidinae (Lepidoptera: Nymphalidae). *PeerJ* **6**, e4311 (2018).
7. Chazot, N. et al. Priors and posteriors in Bayesian timing of divergence analyses: the age of butterflies revisited. *Systematic Biology* **68**, 797–813 (2019).
8. Chazot, N. et al. Conserved ancestral tropical niche but different continental histories explain the latitudinal diversity gradient in brush-footed butterflies. *Nature Communications* **12**, 5717 (2021).
9. Zhang, J., Cong, Q., Shen, J., Opler, P. A. & Grishin, N. V. Genomics-guided refinement of butterfly taxonomy. *The Taxonomic Report of the International Lepidoptera Survey* **9**, 1–54 (2021).
10. Kawahara, A. Y. et al. Phylogenomics reveals the evolutionary timing and pattern of butterflies and moths. *Proceedings of the National Academy of Sciences* **116**, 22657–22663

(2019).
11. Wahlberg, N., Wheat, C. W. & Peña, C. Timing and patterns in the taxonomic diversification of Lepidoptera (butterflies and moths). *PLOS ONE* **8**, e80875 (2013).
12. Heikkilä, M., Kaila, L., Mutanen, M., Peña, C. & Wahlberg, N. Cretaceous origin and repeated tertiary diversification of the redefined butterflies. *Proceedings of the Royal Society B: Biological Sciences* **279**, 1093–1099 (2011).
13. Zhang, L. et al. The water lily genome and the early evolution of flowering plants. *Nature* **577**, 79–84 (2020).
14. Laudanno, G., Haegeman, B., Rabosky, D. L. & Etienne, R. S. Detecting lineage-specific shifts in diversification: A proper likelihood approach. *Systematic Biology* **70**, 389–407 (2021).
15. Kaliszewska, Z. A. et al. When caterpillars attack: Biogeography and life history evolution of the Miletinae (Lepidoptera: Lycaenidae). *Evolution* **69**, 571–588 (2015).
16. Pierce, N. E. & Dankowicz, E. The natural history of caterpillar-ant associations. in *Caterpillars in the Middle* (eds. Marquis, R. & Koptur, S.) (Springer, 2021).
17. Singer, M. C., Ehrlich, P. R. & Gilbert, L. E. Butterfly feeding on Lycopsid. *Science* **172**, 1341 (1971).
18. Hamm, C. A. & Fordyce, J. A. *Selaginella* and the Satyr: *Euptychia westwoodi* (Lepidoptera: Nymphalidae) oviposition preference and larval performance. *Journal of Insect Science* **16**, (2016).
19. Pinkert, S., Barve, V., Guralnick, R. P. & Jetz, W. Global geographical and latitudinal variation in butterfly species richness captured through a comprehensive country-level occurrence database. *Global Ecology and Biogeography* **31**, 830–39. (2022).
20. Scoble, M. J. A catalogue of the Hedylidae (Lepidoptera: Hedyloidea), with descriptions of two new species. *Entomologica Scandinavica* **21**, 113–119 (1990).

Supplementary Table Legends:

Table S1.
Specimen information for samples included in this study. Family-group taxonomy is accurate as of March 2021. I/O = Ingroup (I) or Outgroup (O); DNA = concentration in ng/µl; ABRI = African Butterfly Research Institute, Kenya; ACG = Area de Conservación Guanacaste, Costa Rica; ANIC = Australian National Insect Collection; AWC = Andrew D. Warren Collection, Gainesville, FL, USA; CCNY = City College of New York, USA; CJMC = Chris J. Müller Collection, Australia; CLDZ = Coleção de Lepidoptera, Departamento de Zoologia, Universidade Federal do Rio Grande do Sul, Porto Alegre, Brazil; CMRC = C. Malaver-Rios Collection, Colombia; CSIC-UPF = Consejo Superior de Investigaciones Científicas, Universidad Pompeu Fabra, Barcelona, Spain; DZUP = Departamento de Zoologia, Universidade Federal do Paraná, Curitiba, Brazil; EBC = Ernst Brockmann Collection; FLMNH = Florida Museum of Natural History, USA; GNC = Gregory Nielsen Collection, Colombia; IECA = Biology Centre, Czech Academy of Sciences; KSUC = Kansas State University Museum of Entomological and Prairie Arthropod Research; MCZ = Museum of Comparative Zoology, Harvard University, USA; MGCL = McGuire Center for Lepidoptera & Biodiversity, University of Florida, USA; NHMUK = Natural History Museum, London, UK; NP = National Park; NTUM = National Taiwan University Museum; SCAUB = South China Agricultural University, China; SSC = Szabolcs Sáfián Collection, Hungary; UCDBA = University of Chicago; UMD = University of Maryland, USA; UMUT = University Museum, University of Tokyo; UTSW = University of Texas Southwestern Medical Center, USA; YIC = Yutaka Inayoshi Collection, Chiang Mai, Thailand; YPM = Yale Peabody Museum; ZFMK = Zoological Research Museum Alexander Koenig, Germany; ZFMK = König Museum, Bonn, Germany; ZIN = Zoological Institute of the Russian Academy of Sciences; ZUEC = Museu de Zoologia da Universidade Estadual de Campinas 'Adão José Cardoso', Campinas, Brazil.

Table S2.
Support values for the nine phylogenetic analyses conducted. Family-group taxonomy is accurate as of March 2021. A dash indicates that the corresponding clade was not recovered, "N/A" indicates that the taxon was represented by a single branch and consequently does not have corresponding support values. UFBS = Ultrafast bootstrap 2 support values; SH-aLRT = Shimodaira-Hasegawa approximate likelihood ratio test support values; LPP = Local posterior probabilities.

Table S3.
Percentages of quartets supporting all possible topologies for the phylogenetic hypotheses tested using Four-cluster Likelihood Mapping (FcLM) analyses. Bolded percentages represent the plurality of quartets in a particular analysis. Italicized rows represent topologies present in the ML amino acid tree from Figure 1. "Not fully resolved" rows contain sums of percentages of quartets that are either partially resolved (T12, T13, T23) or unresolved (T*).

Table S4.
Summary of divergence time estimates in treePL, with median ages and age ranges (95% CIs) provided for select clades (in Ma). Ages from columns AL-AN were used in the analysis presented in Fig. 1.

Table S5.
Individual country records for butterflies in the present study. Abbreviations in column C are current ISO 3166-1 alpha-3 country codes.

Table S6. Biogeographic analyses of butterflies showing ancestral areas, tested with different models, areas, and parameters. Column D refers to bioregion character scoring schemes delineated in Table S27 and Figures S11-S13. AM, adjacency matrix; DL, DivaLike; TS, time slices. *Analyses excluding *Baronia brevicornis*.

Table S7.
Summary data for ranges of ancestral butterflies over time. Columns A-H list the number of nodes and long branches spanning at least three bins (i.e., branch length >30 mya) associated with each bioregion at different time periods, binned into 15 mya intervals. Data for individual nodes are provided in columns J-T. Node ages (Column L) were extracted from the dated phylogeny (AA154_secondary_only_strategyB.tre; Data S22). Node labels (Column K) and character state information (Columns M-T) are taken from the BioGeoBears range data (Table S48). Data for long branches are provided in columns V-AF. A bioregion is only recorded for a long branch if it is part of the character state associated with both the beginning and ending of the branch; long branches lacking this criterion are indicated with an 'N/A' in column V.

Table S8.
Temporal and spatial distribution of butterfly families, based on the DECX analysis. An 'x' indicates at least one species in the family (i.e., at least one node in the clade) was estimated to be present in the bioregion in the corresponding column, at the time in the corresponding row. 'Ancestor' indicates the presence of an ancestral node predating the existence of the family.

Table S9.
Larval host records of butterfly species included in our study. “Number_records” refers to the number of records in our database of host interactions. “Ordinal_proportion” is the proportion of host records for the butterfly species that are from hosts in the same host order. “Familial_proportion” is the proportion of host records for butterfly species that are from hosts in the same host family.

Table S10.
Z-scores for each butterfly host plant family. The host family with the largest z-score in its corresponding module had the greatest influence over which butterfly species were assigned to that module.

Table S11.
Posterior probabilities of each state (plant modules) for all nodes estimated in SIMMAP. Refer to Fig. S9 for node labels.

Table S12.
Posterior probabilities of each state (Hemiptera, Hymenoptera (ants), lichens, plants) for all nodes estimated in SIMMAP. Refer to Fig. S9 for node labels.

Table S13.
Host plant richness and divergence across butterfly families. For each species, the number of host plant families (Num.families) and their phylogenetic distance (PD) were calculated using the function 'pd' in the R package picante. This table also shows the mean pairwise distance (mpd.obs), the mean nearest taxon distance (mntd.obs), and their normalized values (mpd.obs.z and mntd.obs.z) using the function 'ses.mpd' and 'ses.mntd' in picante. Normalized values of MPD and MNTD are referred to as the distance-based speciation index (DSI) in the main text.

Table S14.
Summary of parameters and other details of the ML phylogenetic analyses conducted in IQ-TREE. All runs were conducted on the UF HiPerGator2 cluster, except for analyses 5, 6, and 8 which were run on the BYU High Performance Cluster. "bnni" refers to whether the bnni option was applied in tree searches.

Table S15.
Models tested in HiSSE, their parameters and number of transition rates between states. Free parameters for Turnover rate (τ) and Extinction fraction (ε) are given for observed state (0 = absent, 1 = present) and hidden state (A = hidden state absent or B = hidden state present; additional letters C and D are used for four hidden state models). Parameter designations are always equal for both τ and ε for any single model. "Transition rate matrix" specifies the number of different transition rates between states.

Table S16.
Trait state for feeding habit (specialist = 0, generalist = 1). Missing data were coded in HiSSE as state "2", which is considered uncertain.

Table S17.
Trait state for feeding habit (non-Poales feeders = 0, Poales feeders = 1). Missing data were coded in HiSSE as state "2", which is considered uncertain.

Table S18.
Trait state for feeding habit (non-Fabales feeders = 0, Fabales feeders = 1). Missing data were coded in HiSSE as state"2", which is considered uncertain.

Table S19.
Trait state for feeding habit (non-Brassicales feeders = 0, Brassicales feeders = 1). Missing data were coded in HiSSE as state"2", which is considered uncertain.

Table S20.
Trait state for feeding habit (non-Fagales feeders = 0, Fagales feeders = 1). Missing data were coded in HiSSE as state "2", which is considered uncertain.

Table S21.
Trait state for feeding habit for the module that includes Poaceae (non-module 8 feeders = 0, module 8 feeders = 1). Missing data were coded in HiSSE as state "2", which is considered uncertain.

Table S22.
Trait state for feeding habit for the module that includes Fabaceae (non-module 7 feeders = 0, module 7 feeders = 1). Missing data were coded in HiSSE as state "2", which is considered uncertain.

Table S23.
Results examining whether diversification rates vary between different plant categories in HiSSE. Models reported are those with the highest fit; a graphical representation of each model is shown in Fig. S26 (except those with inconclusive results).

Table S24.
Fractions used in HiSSE analyses to correct for sampling bias of different states. Values indicate percentages of described species in each state that are represented in this study.

Table S25.
Estimates of fractions of generalist and specialist butterfly species sampled, assuming a global butterfly species richness of 19,500 species. Values in columns B-E are from host record data in Table S9. Values in column F are from species richness estimates of van Nieukerken et al. (2011), which are thought to be ~3.9% less than current estimates.

Table S26.
Estimates of total butterfly species worldwide that belong to each of the 13 host modules outlined in Fig. S23 and Table S33. Worldwide species counts in column B were derived from an unpublished checklist of global butterfly fauna (G. Lamas, pers. comm.). Fraction values in column I indicate the estimated % of butterfly species in the host module that are present in our dataset.

Table S27.
Summary statistics for countries with butterfly species represented in the biogeographic analyses. Values in columns G-I were used to generate tropicality character matrices used for GeoHiSSE analyses. "ISO" refers to the United Nations ISO-3166 official country code.

Table S28.
Character state assignments for distribution of butterflies.

Table S29.
Character matrices used to assess ancestral states of tropicality in contMap (column B), and to assess diversification rates associated with tropicality in GeoHiSSE (columns C-E).

Table S30.
Comparison of ancestral bioregion estimates for nodes associated with select butterfly clades. Numbers in columns E and H are probabilities that the corresponding bioregion is part of the ancestral range, not the probability that the corresponding bioregion is the entirety of the ancestral range. Discordant results are indicated by red text. Estimates in columns D-E are derived from Analysis I (14-bioregions, DECX) estimates in columns G-H are derived from Analysis IV (7 bioregions, DEC in BioGeoBears). See Table S6 for additional analysis details.

Only probabilities > 0.5 are presented here; full results for all nodes are in Data S20 and Table S48.

Table S31.
Relative colonization rates of butterflies between all combinations of bioregions at 5 Ma intervals. Values in rows 3-24 were multiplied by 10^5 for ease of presentation; original colonization rates are presented in rows 41-62. Rows 27-29 are mean colonization rates for larger time intervals, presented in Figures S2-S4. A = West Palearctic, B = East Palearctic, C = Nearctic, D = Neotropics, E = Afrotropics, F = Indomalaya, G = Australasia.

Table S32.
Host module assignments for butterfly species with known host records in the present study.

Table S33.
List of host taxa (i.e., plant families, insect groups) in each of the 13 network modules.

Table S34.
Summary of permits obtained before collecting and/or exporting specimens used in this study.

Table S35.
List of BUTTERFLY1.0 loci, indicating the presence (1) and absence (0) of that locus for every sample included. Presence means that that locus was captured during sequencing.

Table S36.
Sequences determined to be eight standard deviations from the mean branch length, which were removed from inclusion in the phylogenetic data matrices.

Table S37.
Quartet Fidelity (QF) scores calculated to determine taxa that might have been mislabeled or contaminated.

Table S38.
Datasets, partitioning schemes, and model selection results for all ML phylogenetic analyses conducted in the present study.

Table S39.
Quartet sampling results for the degen359 tree (degenerated nucleotide dataset with 359 partitions). Node labels correspond to those in Data S15.

Table S40.
Quartet sampling results for the aa154 tree (amino acid dataset with 154 partitions). Node labels correspond to those in Data S16.

Table S41.
Grouping schemes for Four-cluster Likelihood Mapping (FcLM) analyses that examined phylogenetic placement of select butterfly clades.

Table S42.
Calibrations used in treePL dating analyses. All secondary calibrations are derived from the dated tree presented in figures 1 and S12 of Kawahara et al. (2019). All fossil calibrations are based on identifications of fossilized Lepidoptera specimens in de Jong (2017). Fossil calibrations marked with an * in column A were excluded from the secondary + fossil treePL analyses because they had already been used to generate the dated tree in Kawahara et al. (2019).

Table S43.
Genus-level sampling fractions used in the BAMM diversification analyses. For each butterfly genus sampled in the tree, Column E shows the proportion of its species that were included, using species richness estimates from Gerardo Lamas (pers. comm.).

Table S44.
Diversification and biogeographic patterns of clades of interest as referred to in Figure 1. These clades of interest are taxonomically significant groups or clades where there was a rate shift with a probability > 0.5. Rate shift probabilities are branch values, whereas lambda rates, beta rates, and the values calculated in GeoHiSSE (evolutionary rates, discrete tropicality character states, and their corresponding probabilities) refer to the nearest node (or tip in the case of I: *Baronia brevicornis*). “Most Probable Bioregions” is a list of all bioregions with > 0.5 probability of being part of the clade's ancestral range, as determined by the BioGeoBears and DECX analyses. Tropicality values represented as a continuous character are derived from the contMap analysis. See extended methods and supplementary text for detailed explanations of the different schemes for scoring tropicality in contMap and GeoHiSSE.

Table S45.
Summary information of models tested for the GeoHiSSE diversification analyses of temperate and tropical butterfly fauna. Model 4 was found to be the best-fitting model in all analyses.

Table S46.
Branch support values from recent phylogenetic analyses of butterflies. Values from the present study are compared to those of Chazot et al. (2019), Heikkilä et al. (2011), and Wahlberg et al. (2005). Results from Espeland et al. (2018) are excluded because most of the data in that study were included in the present study. “N/A” means monophyly cannot be assessed for that study due to insufficient taxon sampling.

Table S47.
Tip net diversification rates obtained using the command ‘getTipRates’ in BAMM.

Table S48.
Posterior probabilities for possible ranges for all nodes estimated in BioGeoBEARS using DEC. W = West Palearctic, E = East Palearctic, R = Nearctic, N = Neotropics, A = Afrotropics, I = Indomalaya, U = Australasia. Refer to Fig. S9 for node labels.

Table S49.
Posterior probabilities for possible ranges for all nodes estimated in BioGeoBEARS using DEC (excluding *Baronia*). W = West Palearctic, E = East Palearctic, R = Nearctic, N = Neotropics, A = Afrotropics, I = Indomalaya, U = Australasia. Refer to Fig. S22 for node labels.

Table S50.
Posterior probabilities for possible ranges for all nodes estimated in BioGeoBEARS using DIVALike. W = West Palearctic, E = East Palearctic, R = Nearctic, N = Neotropics, A = Afrotropics, I = Indomalaya, U = Australasia. Refer to Fig. S9 for node labels.

Table S51.
Marginal clade-specific rates of phenotypic evolution (beta) of tropicality from BAMM for every node as in Fig. S9, rates extracted with getCladeRates command.

Table S52.
Posterior probabilities of each state (specialist or generalist) for all nodes estimated in SIMMAP. Refer to Fig. S9 for node labels.

Data File Legends

Data S1.
Boxplot of butterfly net diversification rates and net diversification rate estimations, relative to tropicality, from GeoHiSSE. Branch outlines indicate relative diversification rates, with the lowest rates colored blue and the highest rates colored red. Internal branch colors indicate tropicality character states (white = temperate; black = tropical). Yellow branches recovered by GeoHiSSE indicate a hypothetical intermediate 'widespread' state, indicating a transition between temperate and tropical.

Data S2.
Digitized images of butterflies included in the present study for DNA extraction.

Data S3.
Concatenated supermatrices of the amino acid, nucleotide, and degenerated nucleotide datasets used for the maximum likelihood tree searches.

Data S4.
Adjacency matrices utilized in BioGeoBEARS analyses.

Data S5.
Results from the biogeographic stochastic mapping analysis in BioGeoBEARS, used to estimate relative colonization, emigration, immigration, and speciation rates. Data from this file were used to generate the emigration and immigration bar graphs in Fig. S6.

Data S6.
List of data sources for host records in Table S9.

Data S7.
Input file for ancestral state reconstruction of generalist vs. specialist feeding behavior. Species with missing data are coded as having equal probabilities associated with all character states.

Data S8.
Input file for ancestral state reconstruction of plant-feeding, lichen-feeding, and insect-feeding. Species with missing data are coded as having equal probabilities associated with all character states.

Data S9.
Input file for ancestral state reconstruction of feeding behavior by host module. Species with missing data are coded as having equal probabilities associated with all character states.

Data S10.
Permits obtained before collecting and/or exporting specimens used in this study.

Data S11.
Iterative Baited Assembly (IBA) Python script (Breinholt et al. 2018) modified for use on single-end transcriptome data.

Data S12.
Flow chart for manual alignment decision making.

Data S13.
Tree file associated with Table S37. Node labels are QuartetSampling node numbers.

Data S14.
Partitions and model selection results.

Data S15.
Quartet sampling results mapped onto the topology from the degen359 analysis (degenerated dataset, 359 partitions).

Data S16.
Quartet sampling results mapped onto the topology from the aa154 analysis (amino acid dataset, 154 partitions).

Data S17.
Input files for four-cluster likelihood mapping (FcLM) analyses testing four taxonomic hypotheses, as described in section 12 (Topology tests) of Extended Online Methods. Data are primarily organized into two subdirectories based on the initial dataset (degen or amino acid) and secondarily organized into subdirectories based on the taxon associated with the hypothesis being tested: Libytheinae, Malazinae, Papilioninae, and Pierinae. Each subdirectory includes data subsets for its respective hypothesis (fasta files of original data and permuted data (permutations I-III)), partition files (Nexus format), and a nexus file with four defined taxonomic groups.

Data S18.
Input file with sampling fractions utilized in the BAMM analysis.

Data S19.
Script used for the lineage-specific birth-death shift analysis in RevBayes (modified from the file available at https://revbayes.github.io/tutorials/divrate/branch_specific.html)

Data S20.
Input and result files associated with DECX analyses.

Data S21.
Maximum likelihood tree files.

Data S22.
Time-calibrated trees (.tre format) obtained from treePL analyses.

Data S23.
Output files on branch and trait rates as estimated by BAMM.

Data S24.
Diversification rates estimated by the lineage-specific birth-death shift analysis in RevBayes.

Data S25.
ContMap ancestral state reconstruction of tropicality as a continuous character, from 0 (red; temperate) to 1 (green; tropical). See methods text for explanation of how tropicality was quantified for this analysis. Numbers on the node labels also correspond to node numbers referenced in trees from the GeoHiSSE analyses.

Data S26.
Transition, speciation, and extinction rates for eight analyses conducted with HiSSE.

Data S27.
HiSSE output of lamba, mu, and transition rates for the best fit model (Model 16) as selected by AIC for specialist versus generalist feeding behavior in Papilionoidea.

Data S28.
HiSSE output of lamba, mu, and transition rates for the best fit model (Model 16) as selected by AIC for specialist versus generalist feeding behavior in Hesperiidae.

A Time-Calibrated Global Phylogeny of Butterflies

Kawahara et al. (2023): Nature Ecology and Evolution

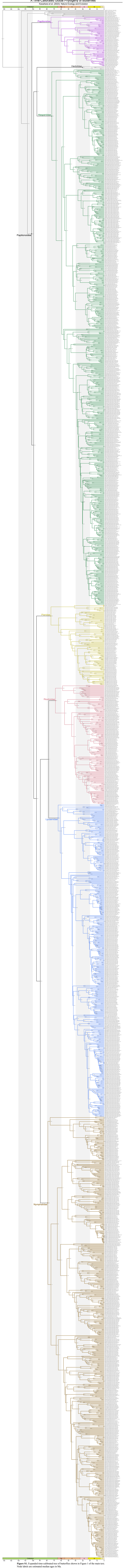

Figure S1. Expanded time-calibrated tree of butterflies shown in Figure 1 of the main text. Node labels are estimated median ages in Ma.

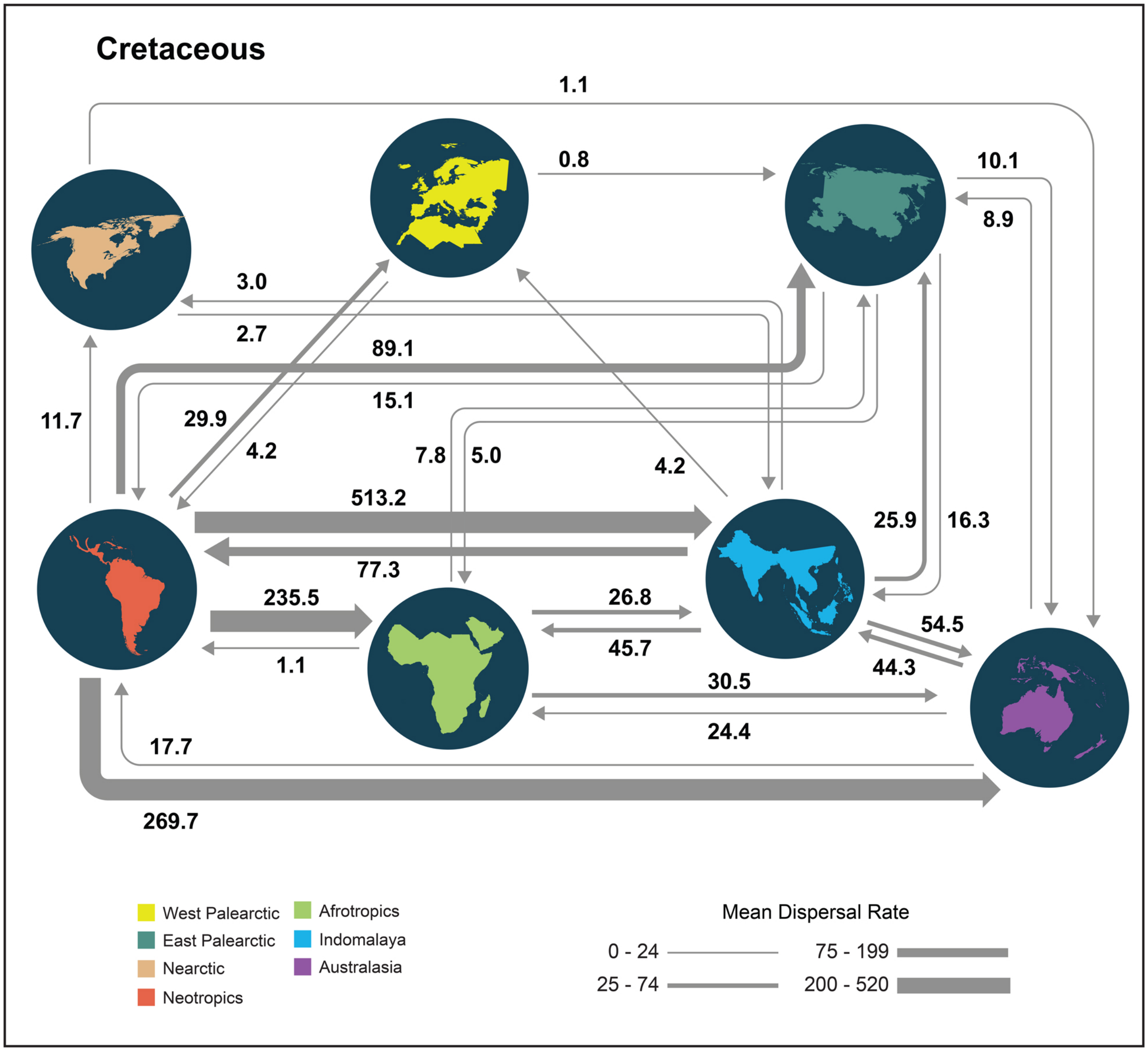

Figure S2. Relative dispersal rates of butterflies between bioregions during the Cretaceous, based on the seven-bioregion BioGeoBears analysis (Analysis IV, Table S6).

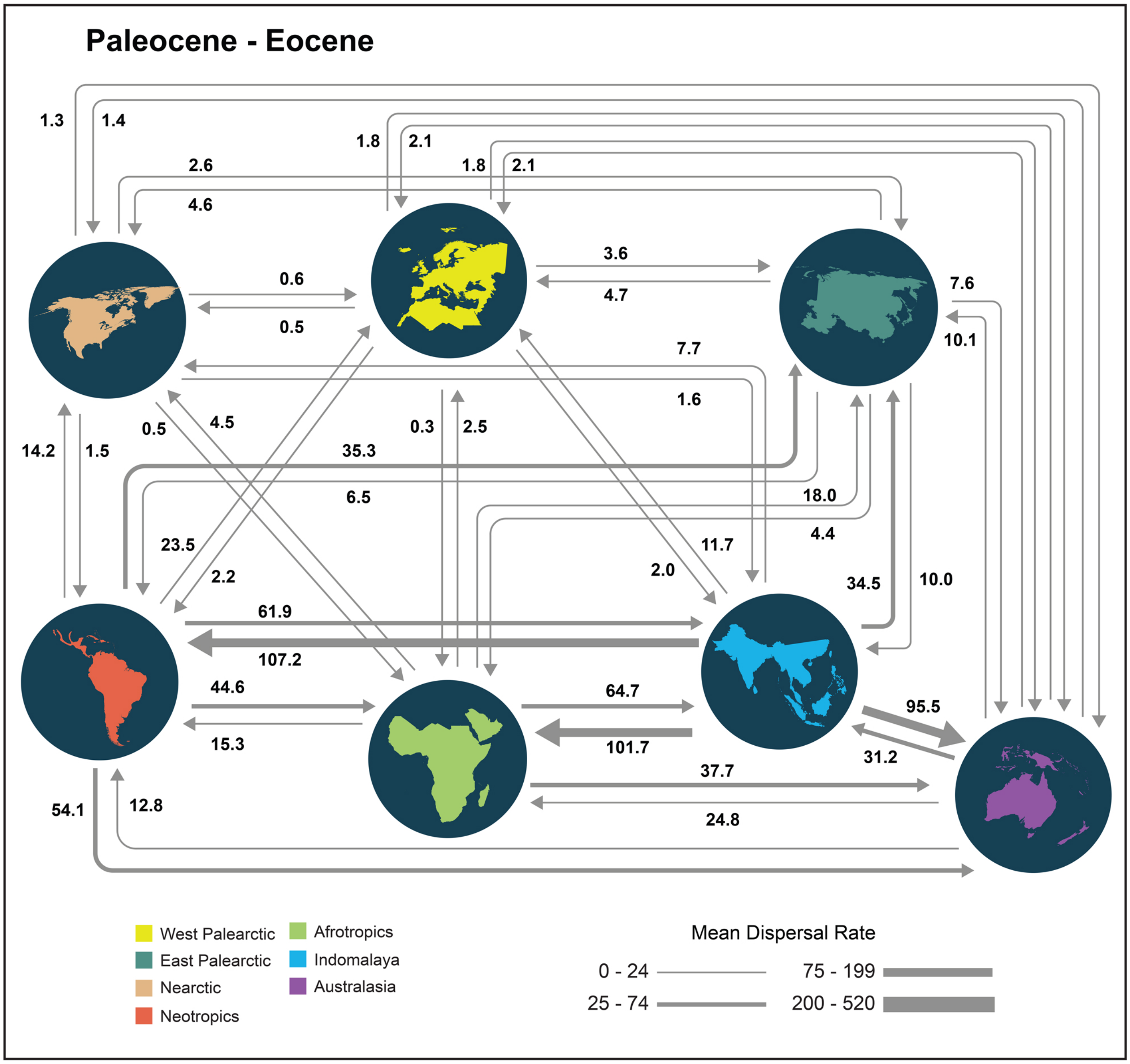

Figure S3. Relative dispersal rates of butterflies between bioregions during the Paleocene and Eocene, based on the seven-bioregion BioGeoBears analysis (Analysis IV, Table S6).

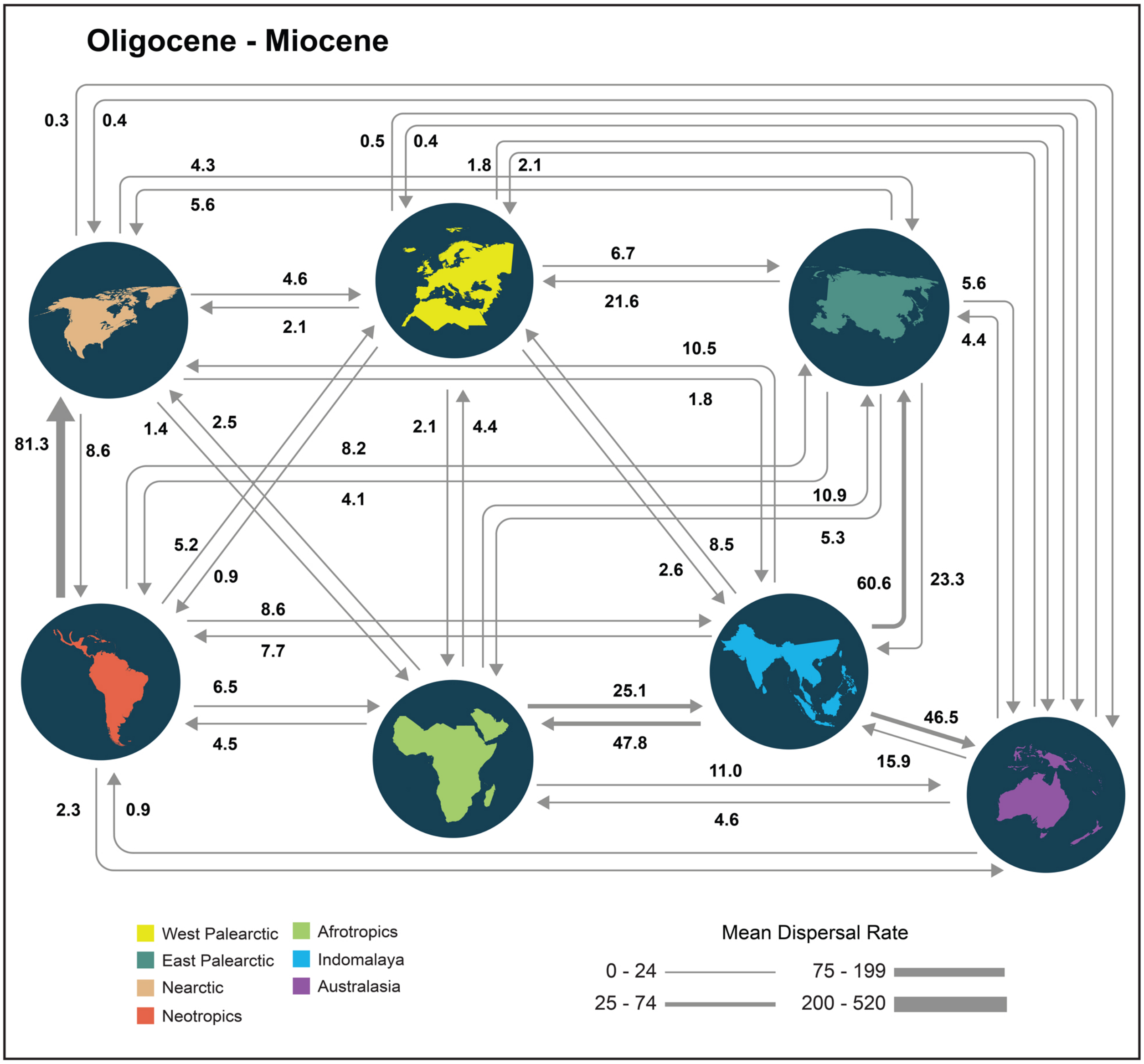

Figure S4. Relative dispersal rates of butterflies between bioregions during the Oligocene and Miocene, based on the seven-bioregion BioGeoBears analysis (Analysis IV, Table S6).

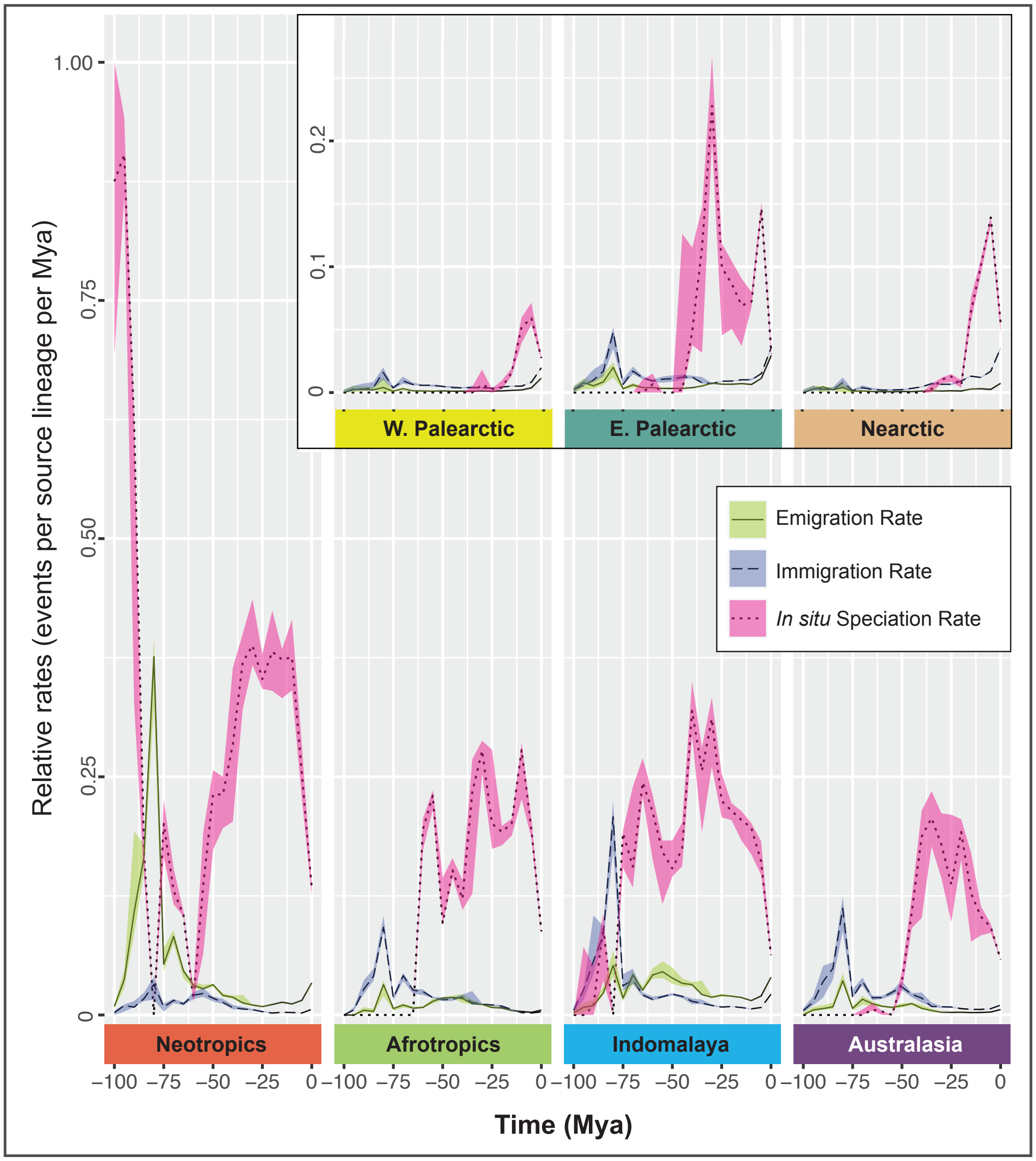

Figure S5. Within-area dispersal and speciation rates of butterflies through time in seven biogeographic regions, based on 100,000 biogeographic stochastic mappings under the DEC model in BioGeoBEARS. Lines are median values; colored ribbons are the lower and upper quartiles (0.25 and 0.75 quantiles).

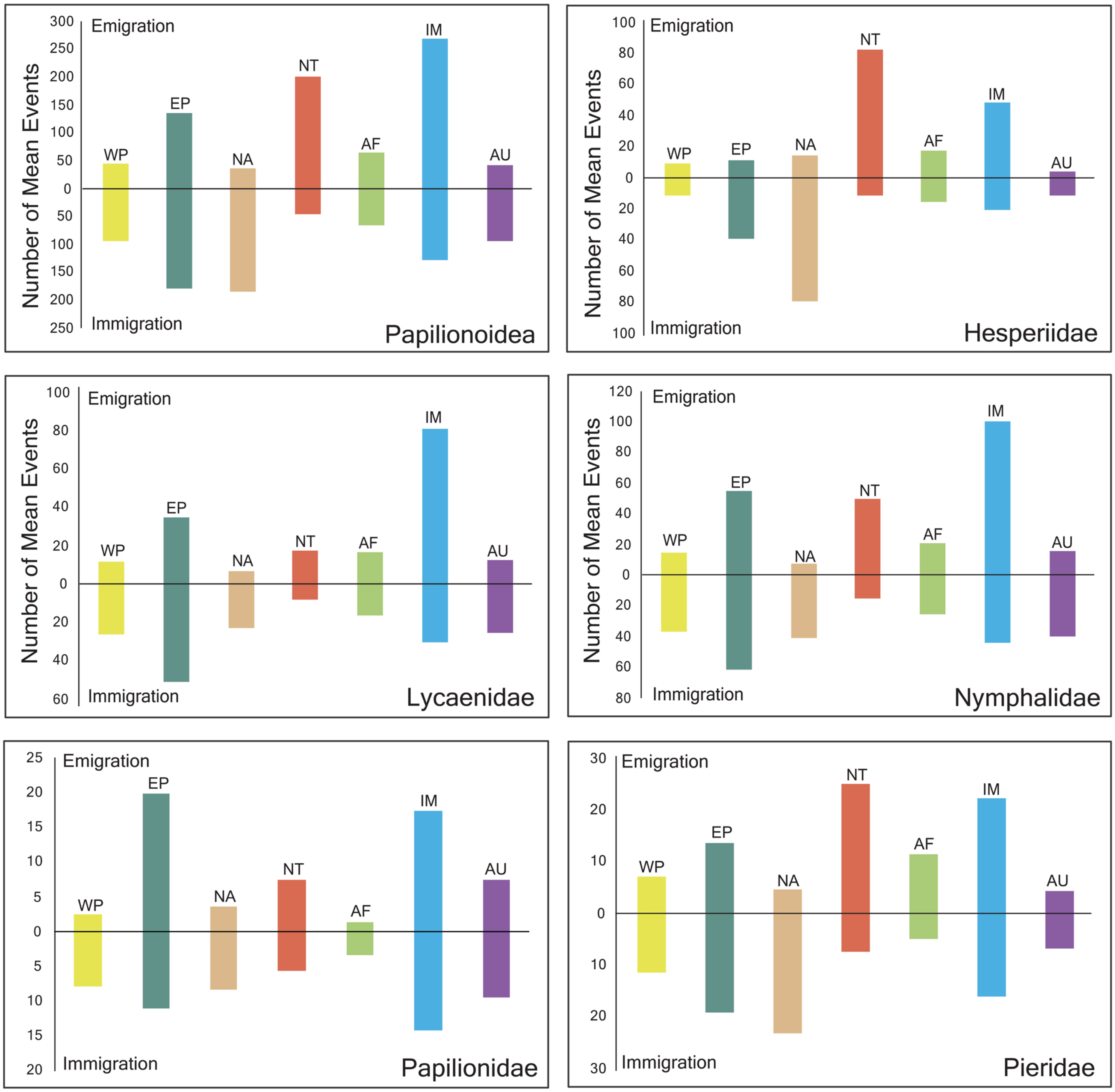

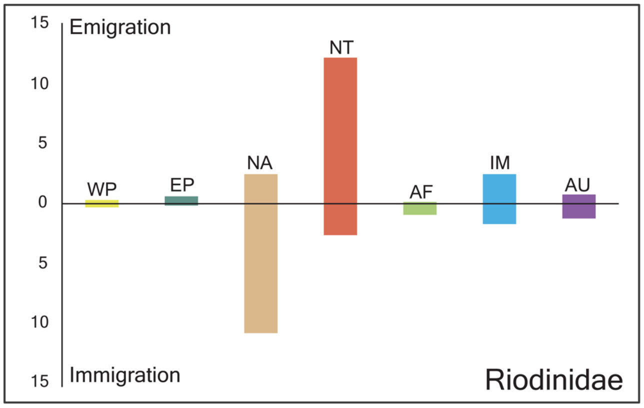

Figure S6. Bar graphs showing the average number of emigration (above 0) and immigration (below 0) events of each area (WP = West Palearctic, EP = East Palearctic, NA = Nearctic, NT = Neotropics, AF = Afrotropics, IM = Indomalaya, AU = Australasia) for all butterflies (Papilionoidea) and each butterfly family excluding Hedylidae. Complete average dispersal events between each type are shown in Table S31.

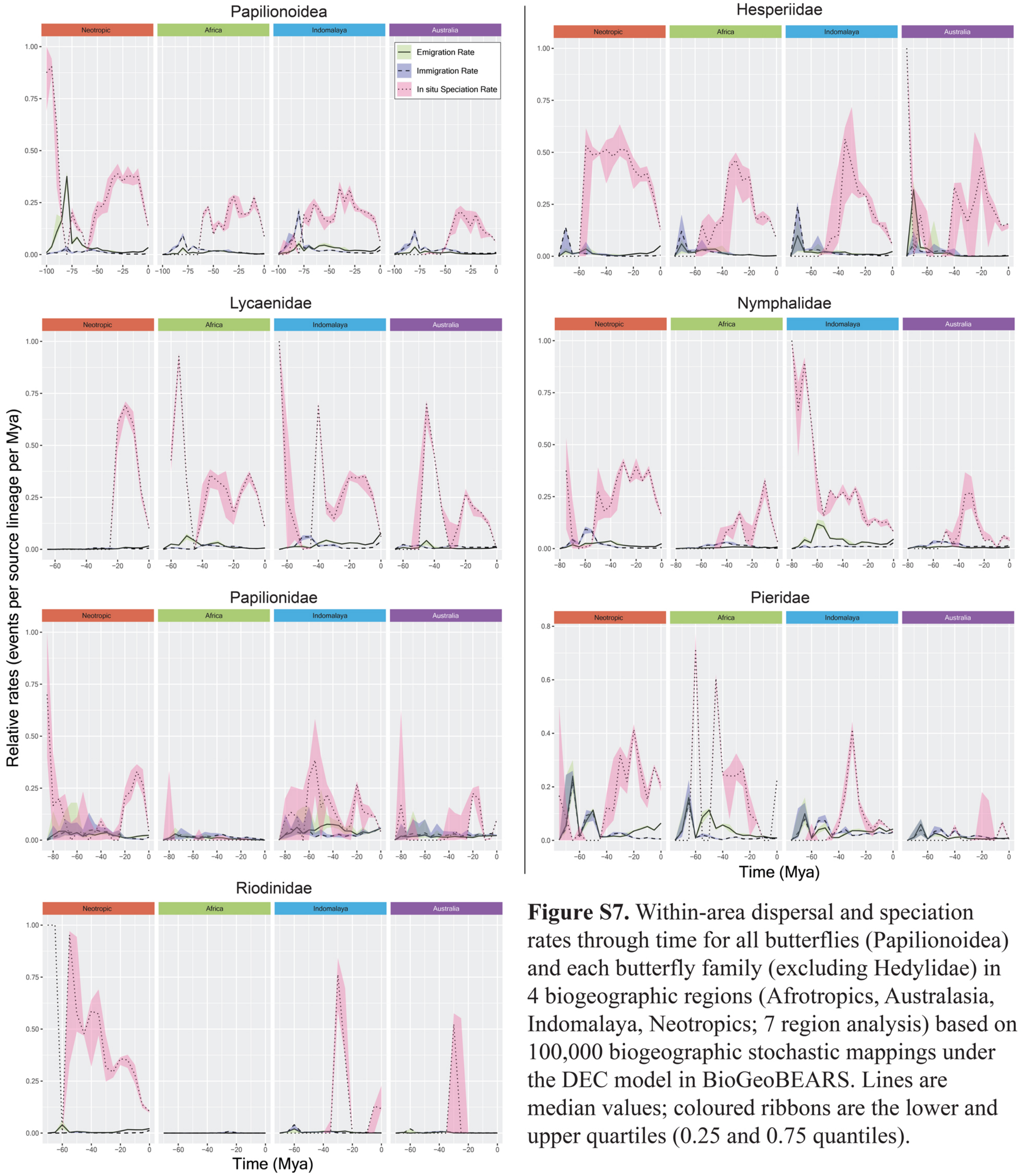

Figure S7. Within-area dispersal and speciation rates through time for all butterflies (Papilionoidea) and each butterfly family (excluding Hedylidae) in 4 biogeographic regions (Afrotropics, Australasia, Indomalaya, Neotropics; 7 region analysis) based on 100,000 biogeographic stochastic mappings under the DEC model in BioGeoBEARS. Lines are median values; coloured ribbons are the lower and upper quartiles (0.25 and 0.75 quantiles).

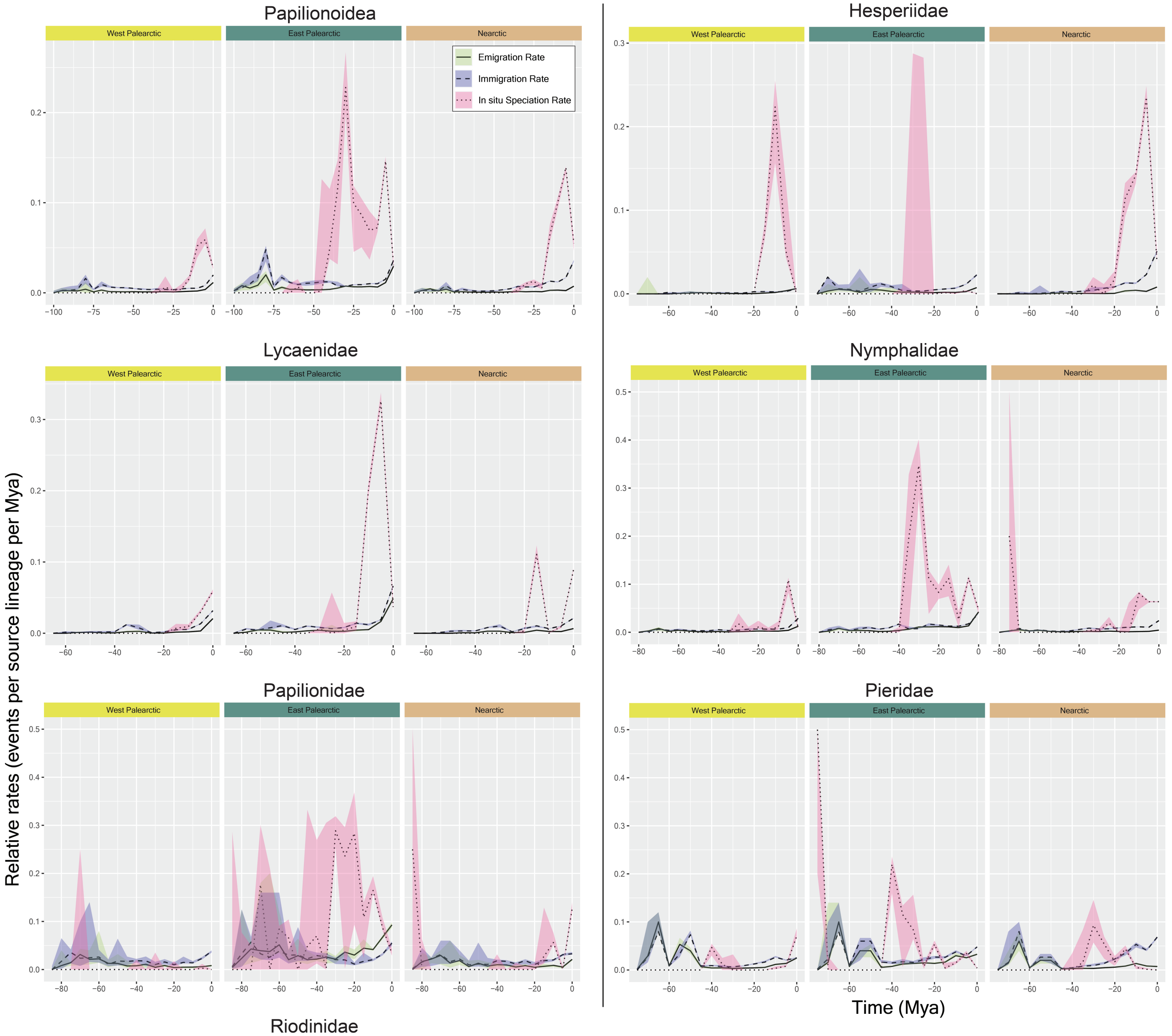

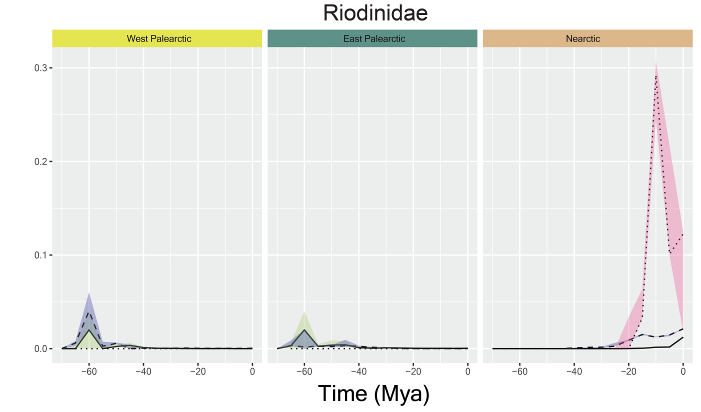

Figure S8. Within-area dispersal and speciation rates through time for all butterflies (Papilionoidea) and each butterfly family (excluding Hedylidae) in 3 biogeographic regions (Nearctic, East Palearctic, West Palearctic; 7 region analysis) based on 100,000 biogeographic stochastic mappings under the DEC model in BioGeoBEARS. Lines are median values; coloured ribbons are the lower and upper quartiles (0.25 and 0.75 quantiles).

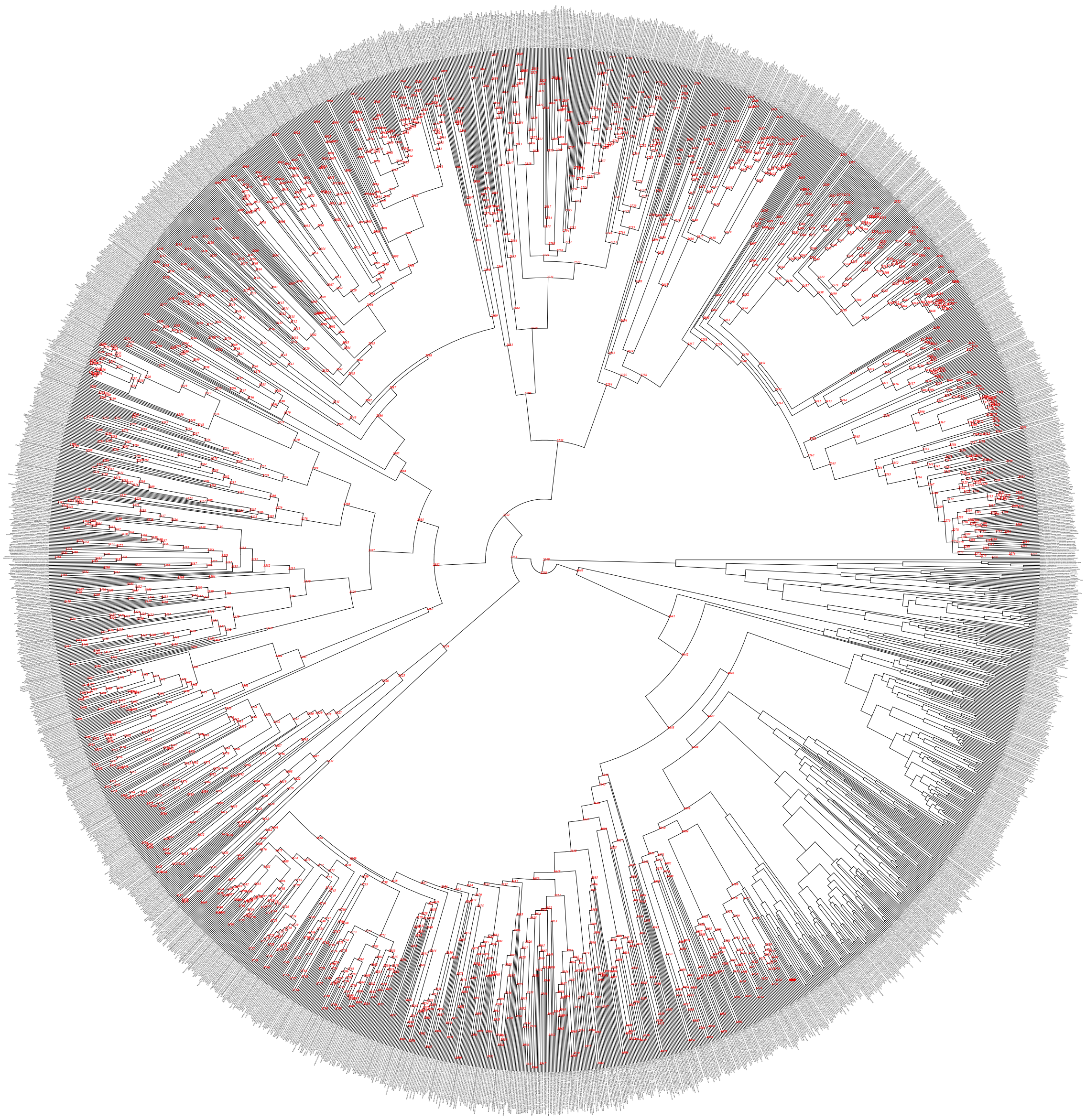

Figure S9. Dated amino acid (aa154) tree showing node labels at all splits.

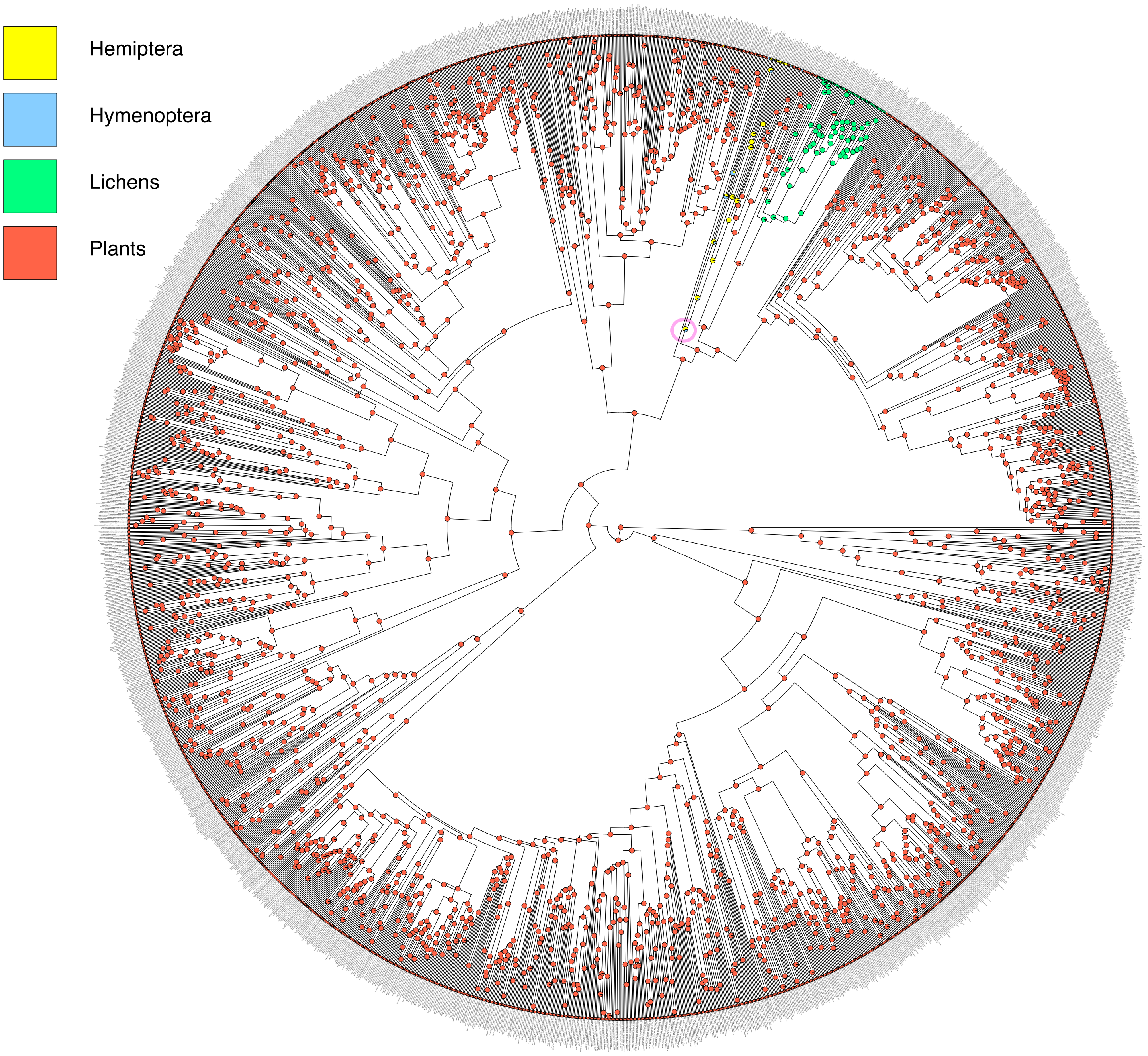

Figure S10. Ancestral state reconstruction of four butterfly feeding strategies (plant, lichen, Hemiptera, and Hymenoptera) conducted in SIMMAP. The pink circle indicates the evolutionary shift from feeding on plants to feeding on other insects.

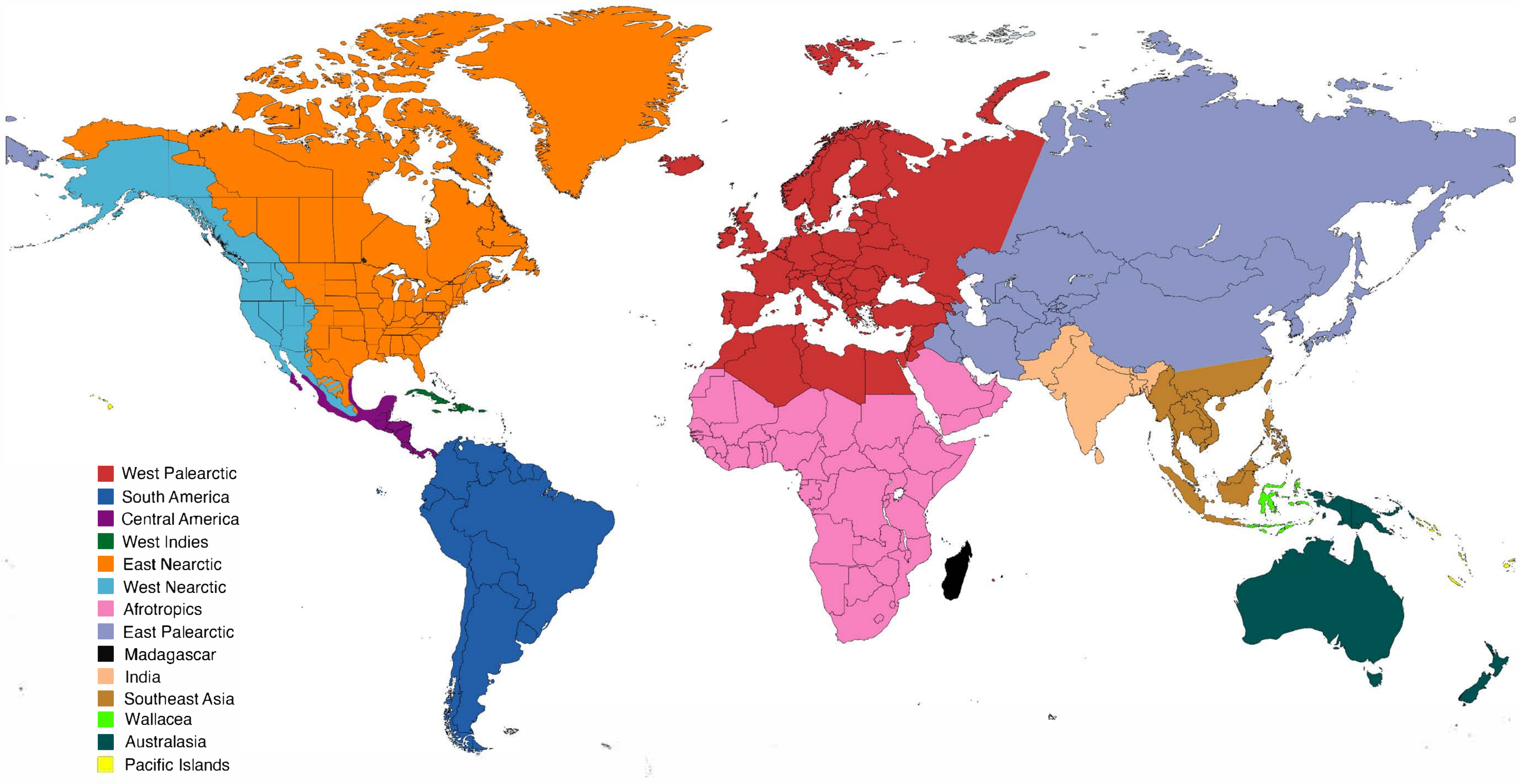

Figure S11. Delineations of areas in the 14-bioregion scheme for DECX.

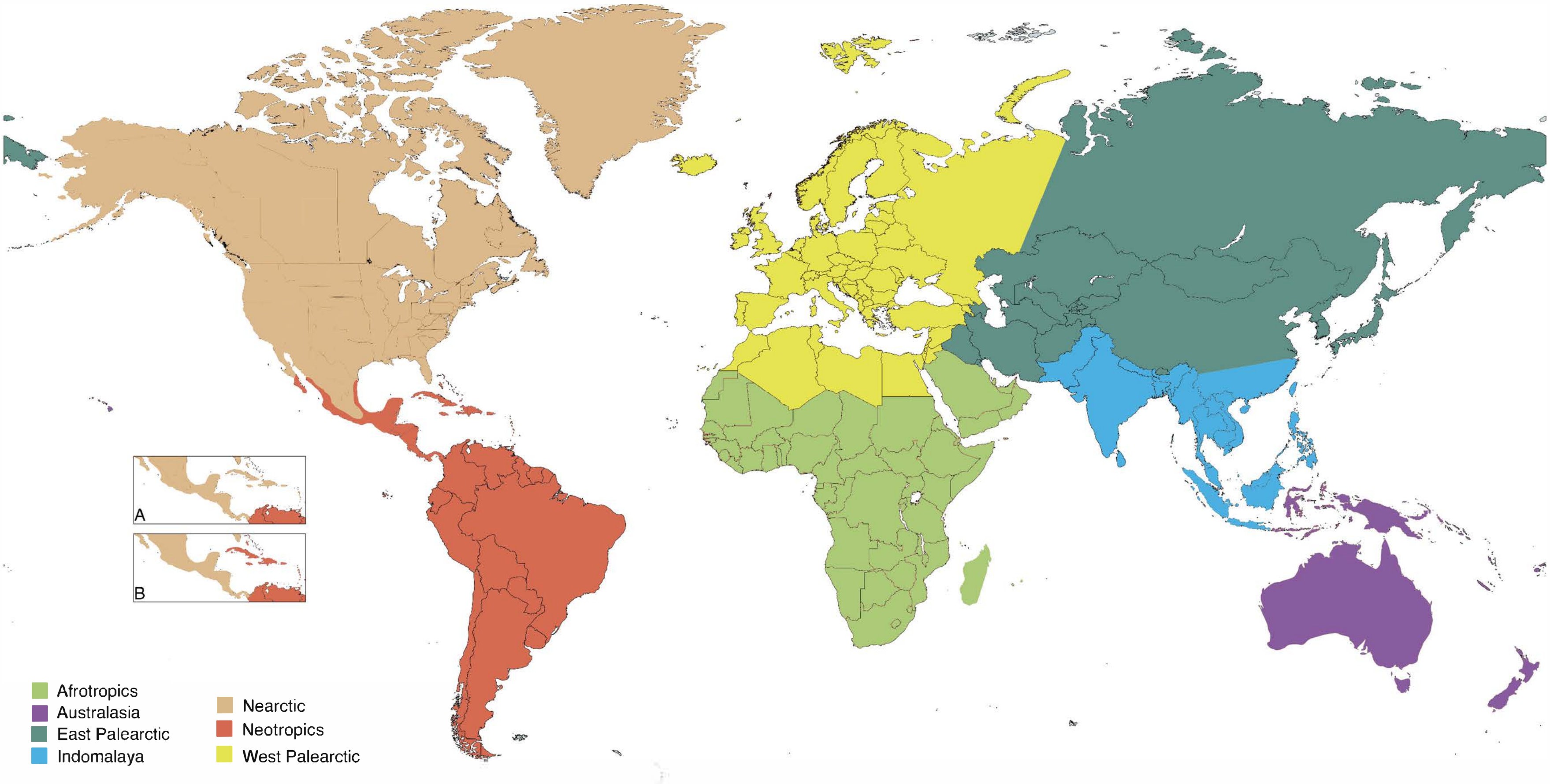

Figure S12. Delineations of areas in the 7-bioregion scheme for BioGeoBears. **A)** Alternate scheme where Central America and the West Indies are coded as Nearctic instead of Neotropical. **B)** Alternate scheme where Central America is coded as Nearctic, but the West Indies remain Neotropical.

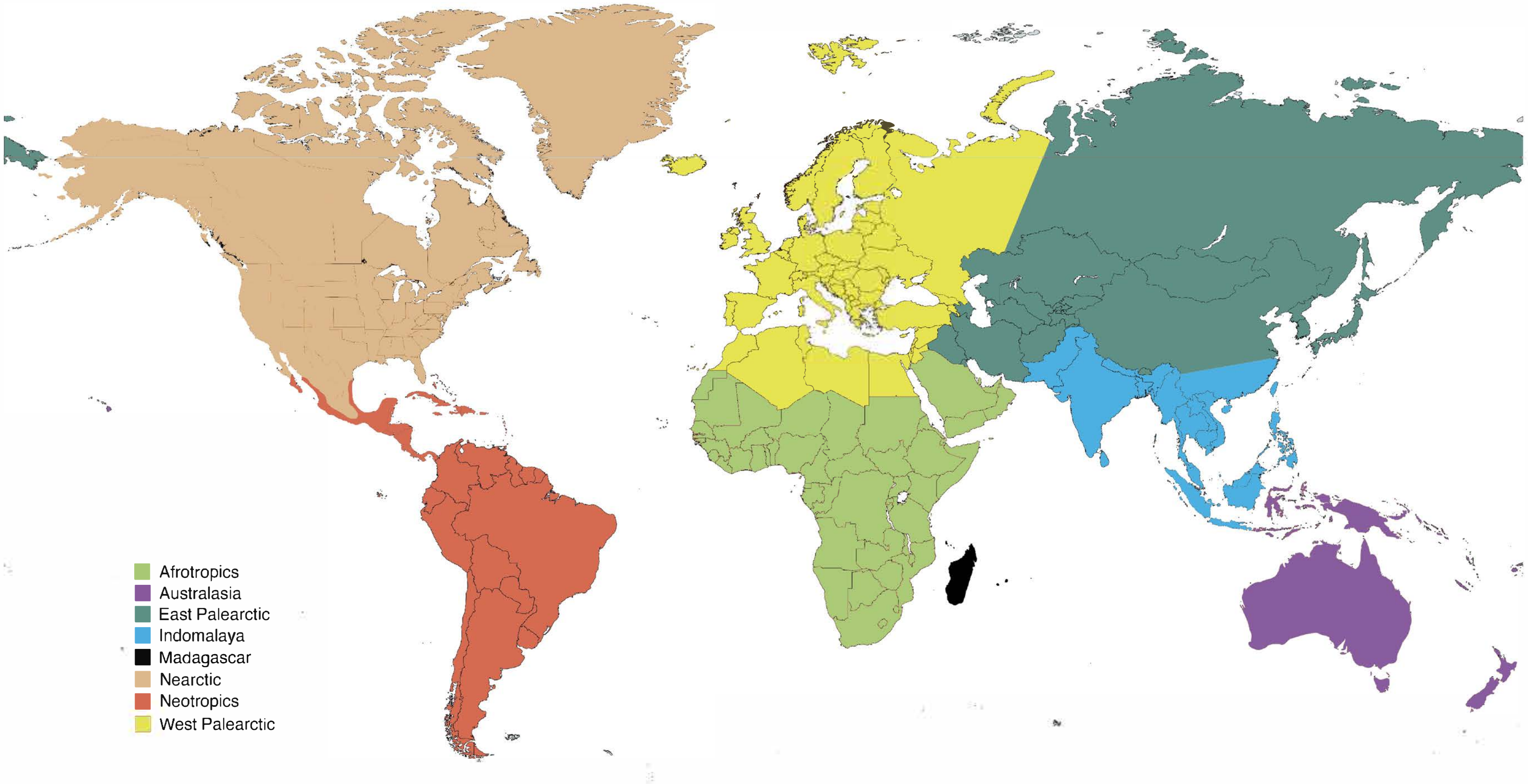

Figure S13. Delineations of areas in the 8-bioregion scheme for DECX.

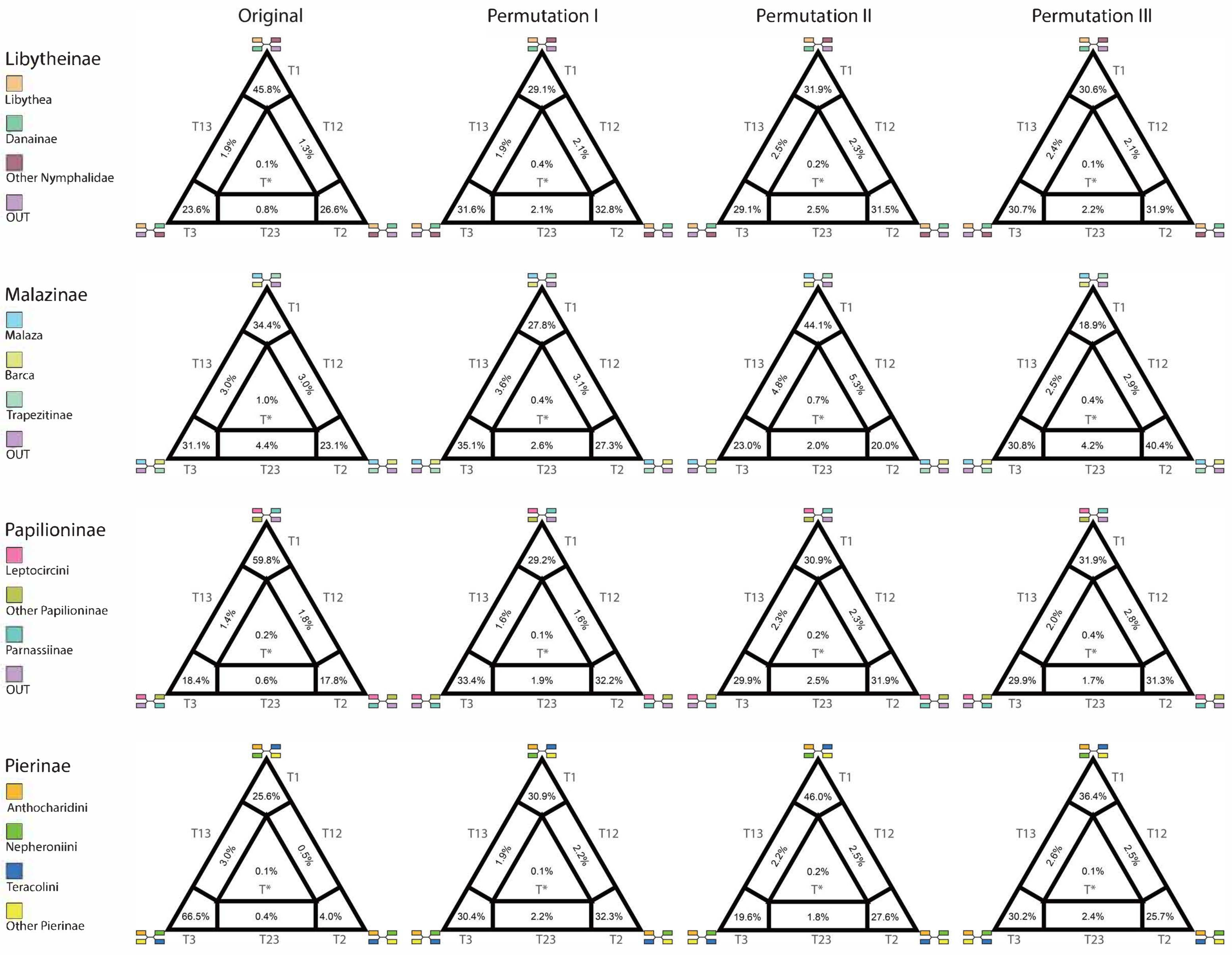

Figure S14. Results of the amino acid Four-cluster Likelihood Mapping (FcLM) analyses, showing quartet support (in %) of all drawn quartets mapped onto 2D simplex graphs for possible topologies.
2D simplex graphs from left to right: original data, permutation I, II, III.

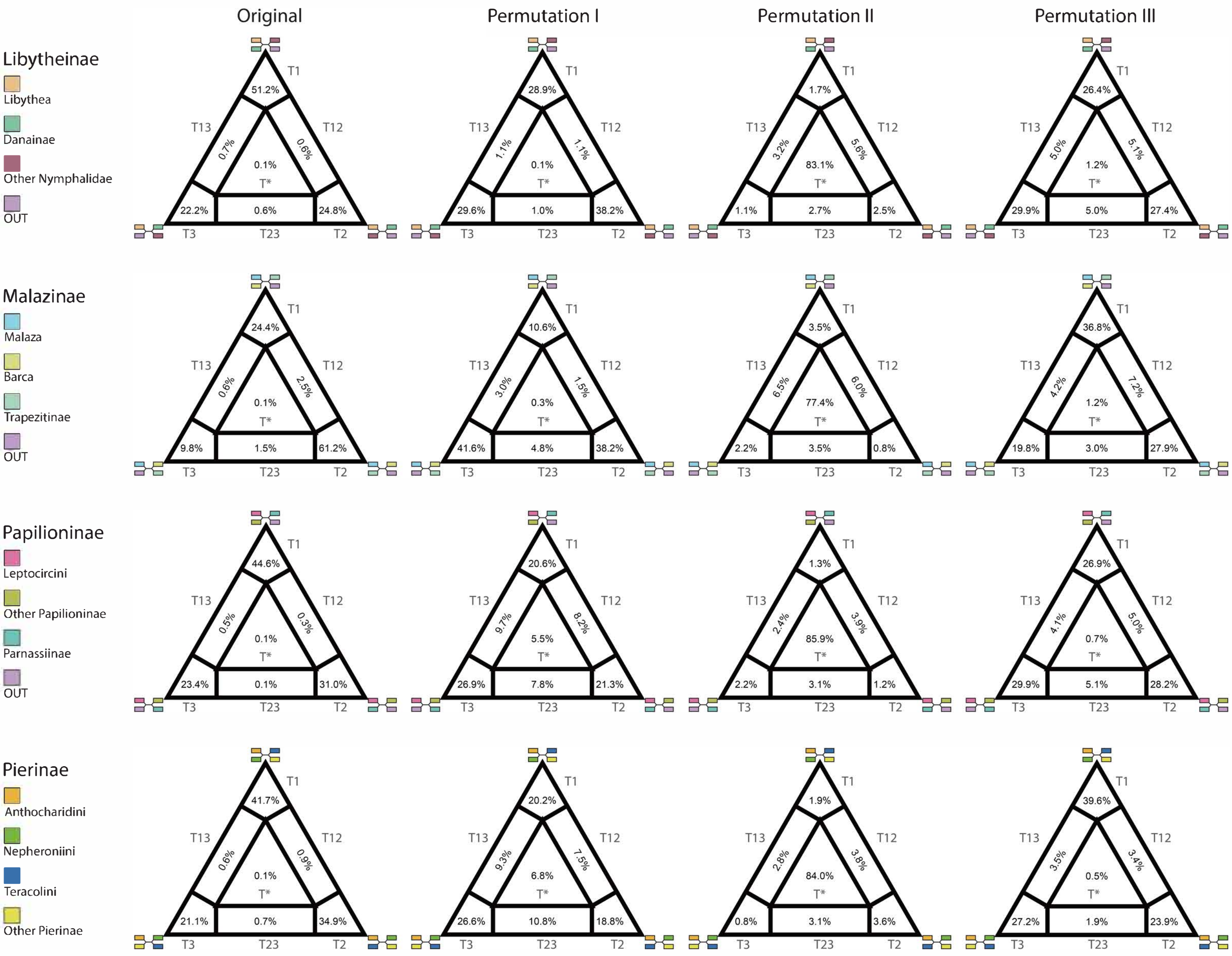

Figure S15. Results of the degen Four-cluster Likelihood Mapping (FcLM) analyses, showing quartet support (in %) of all drawn quartets mapped onto 2D simplex graphs for possible topologies. 2D simplex graphs from left to right: original data, permutation I, II, III.

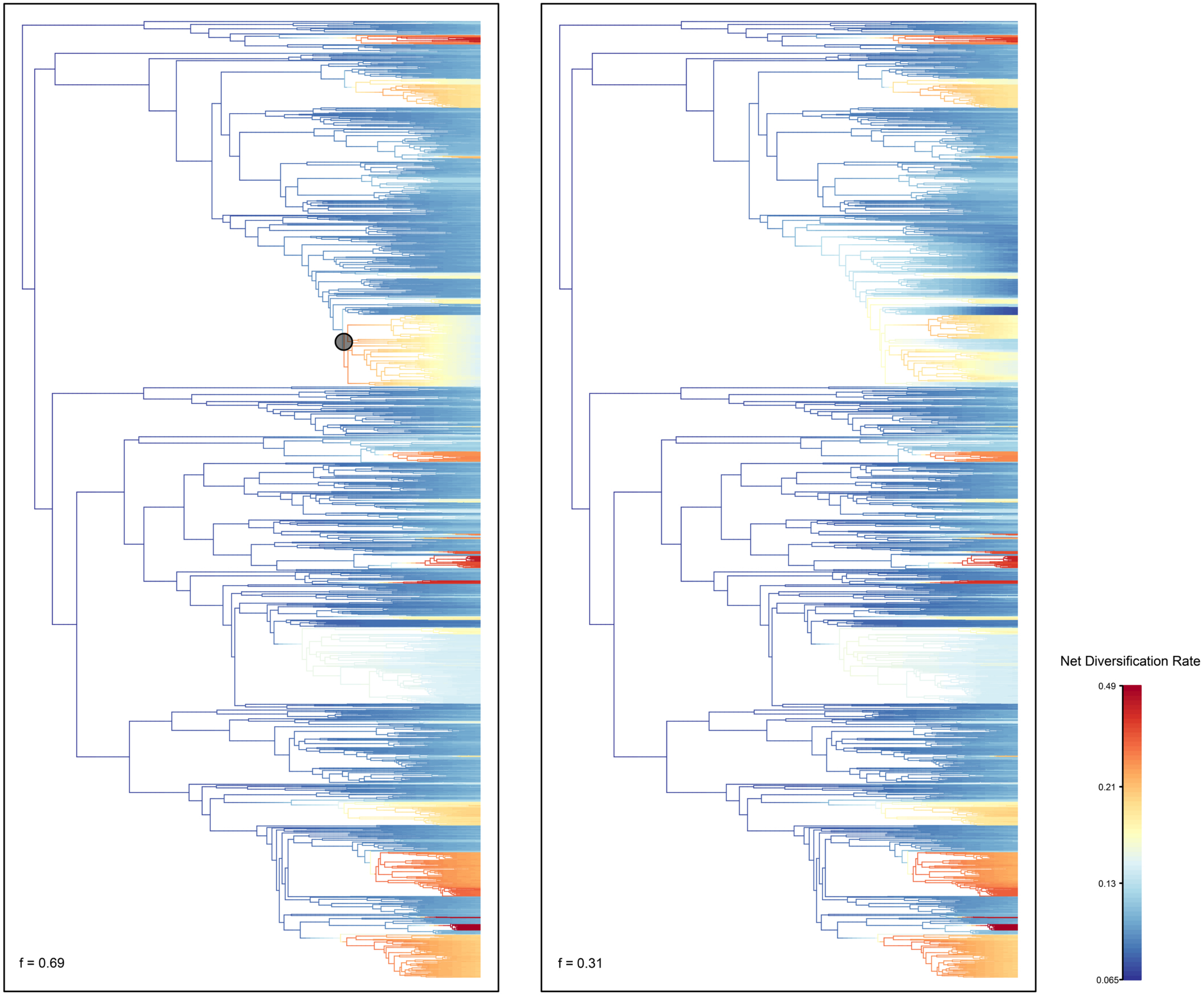

Figure S16. Ninety-five percent credible shift set in BAMM with an expected number of shifts set to 30 and a threshold of 1000, set generated with the ‘credibleShiftSet’ command.

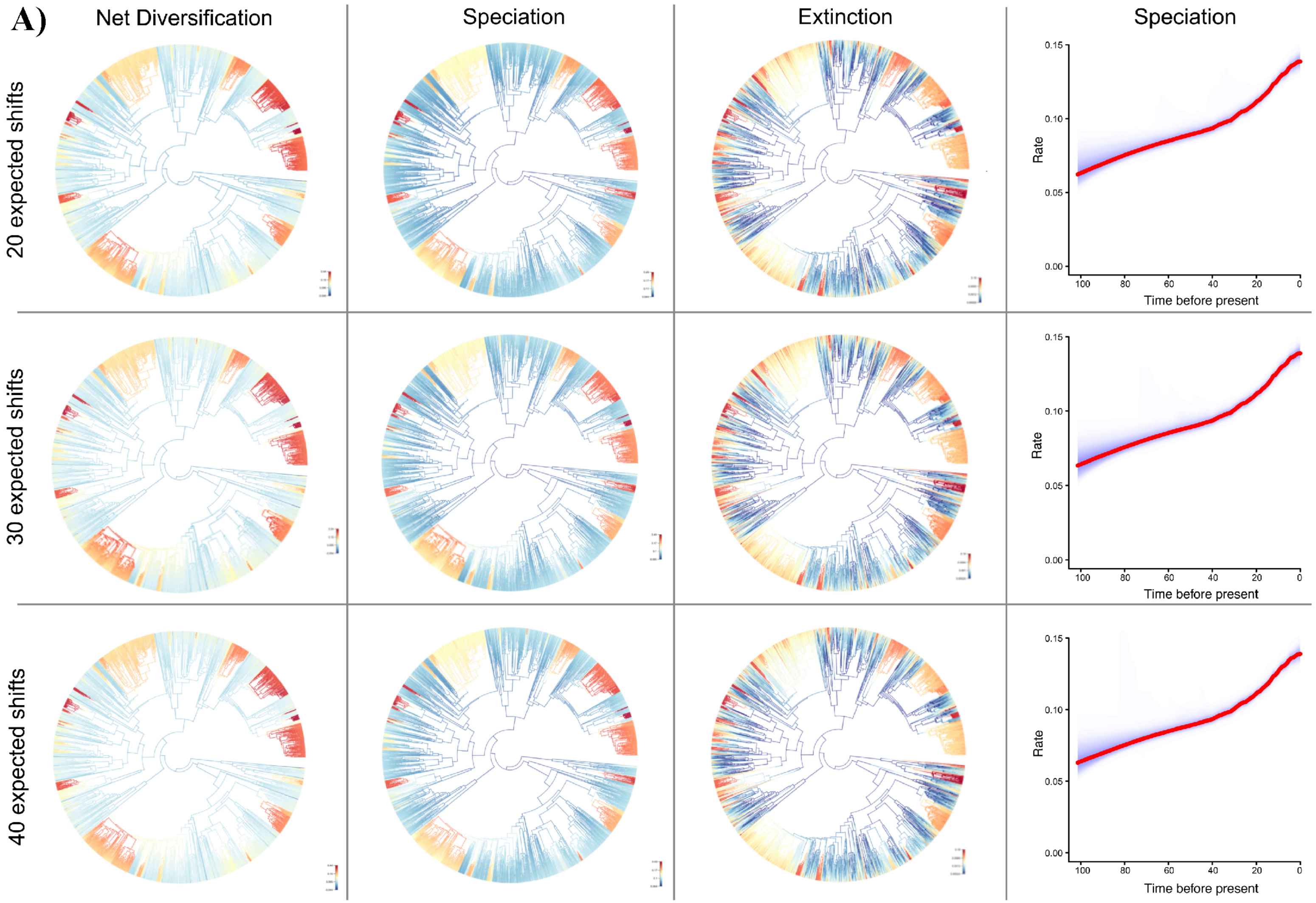

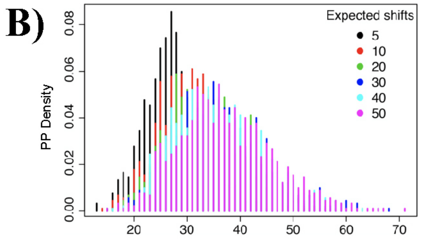

Figure S17. A) BAMM outputs showing how applying three different expected numbers of shift priors (20, 30, 40) does not significantly impact estimates of net diversification, speciation, and extinction rate through time. **B)** Posterior probability distribution of the BAMM tree shown in Fig. 1, with expected number of diversification rate shifts, converging around 30.

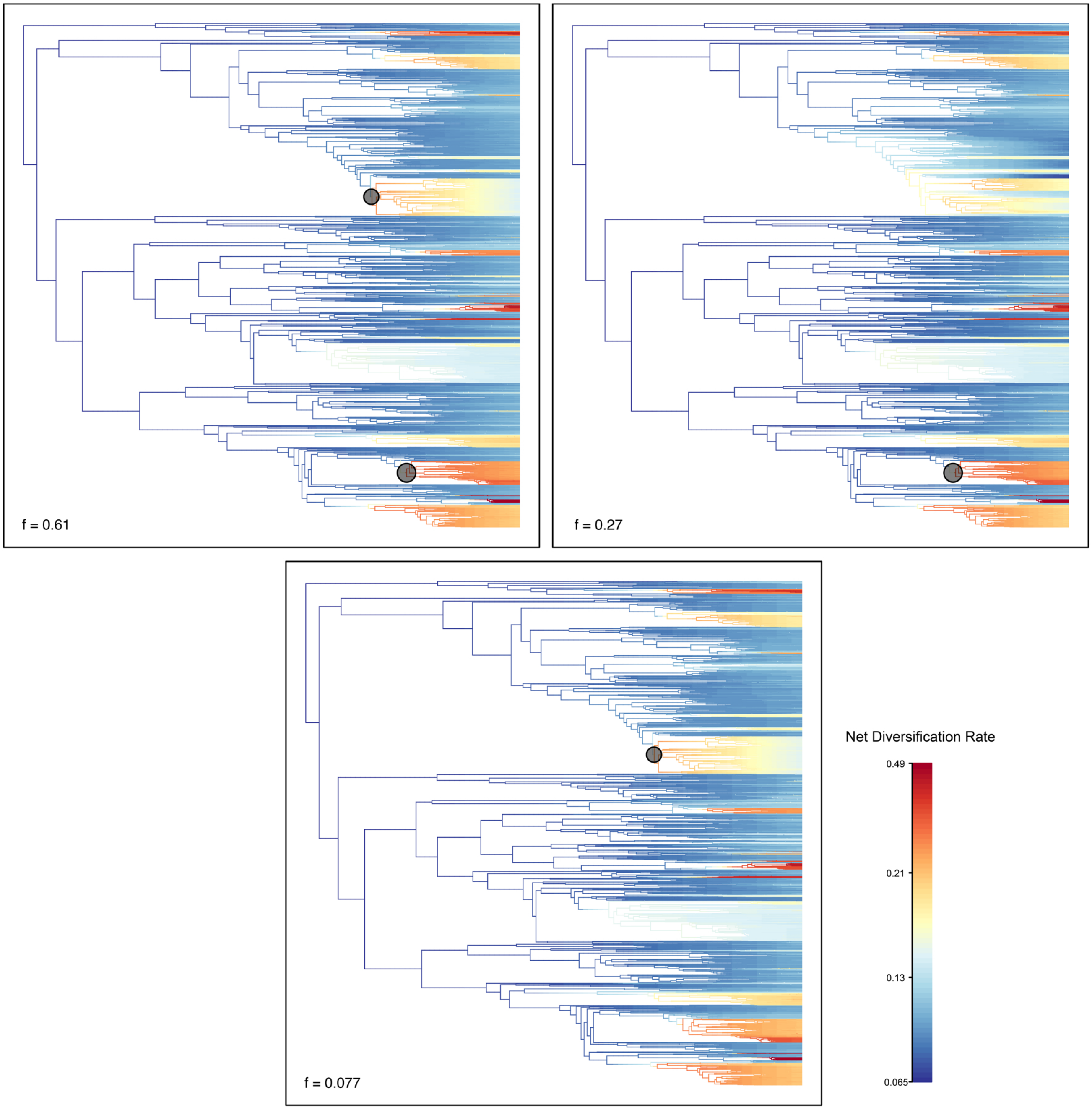

Figure S18. Ninety-five percent credible shift set in BAMM with an expected number of shifts set to 30 and a threshold of 500, set generated with the 'credibleShiftSet' command.

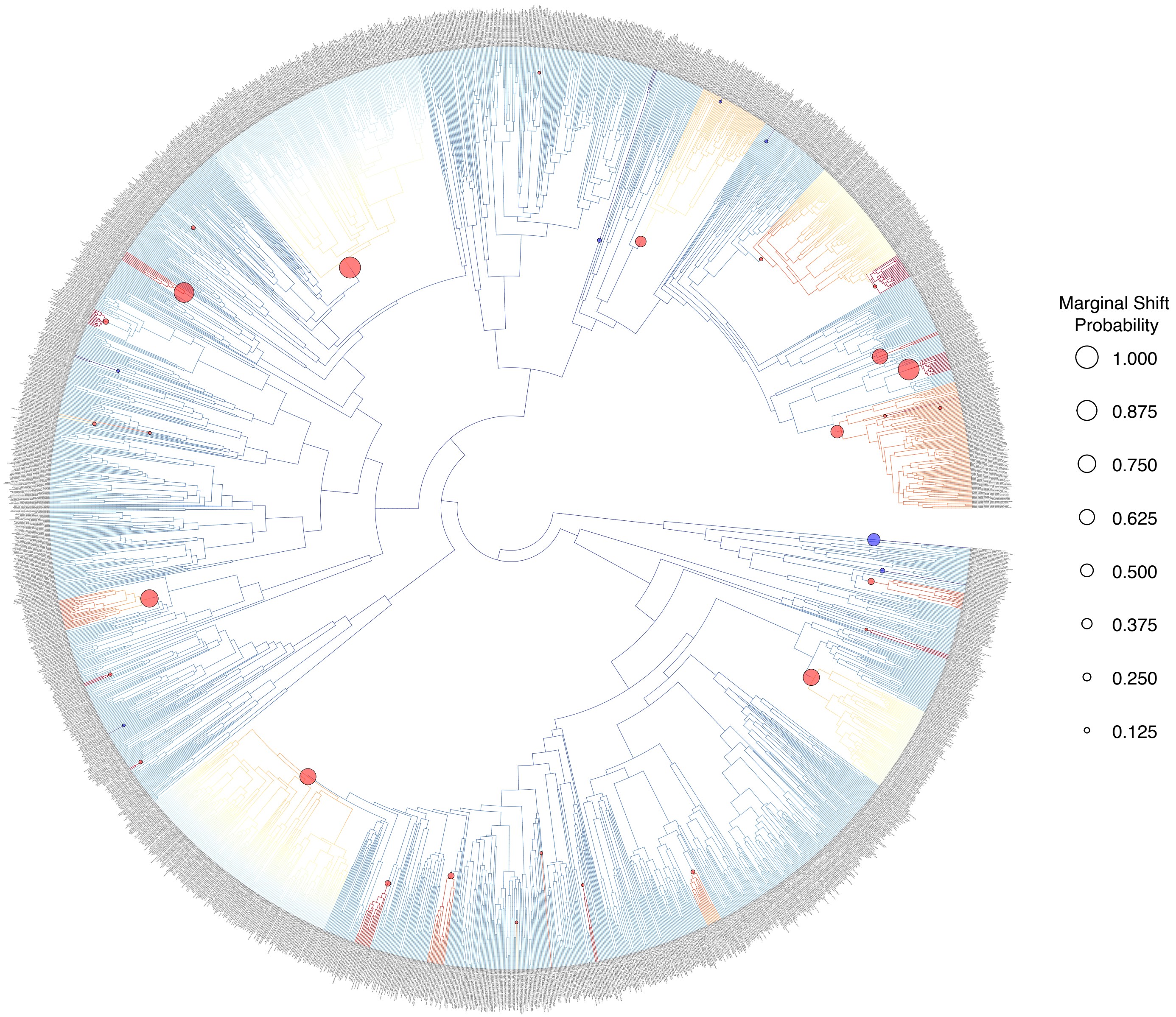

Figure S19. Distinct shift configuration of BAMM, as shown in Fig. 1.

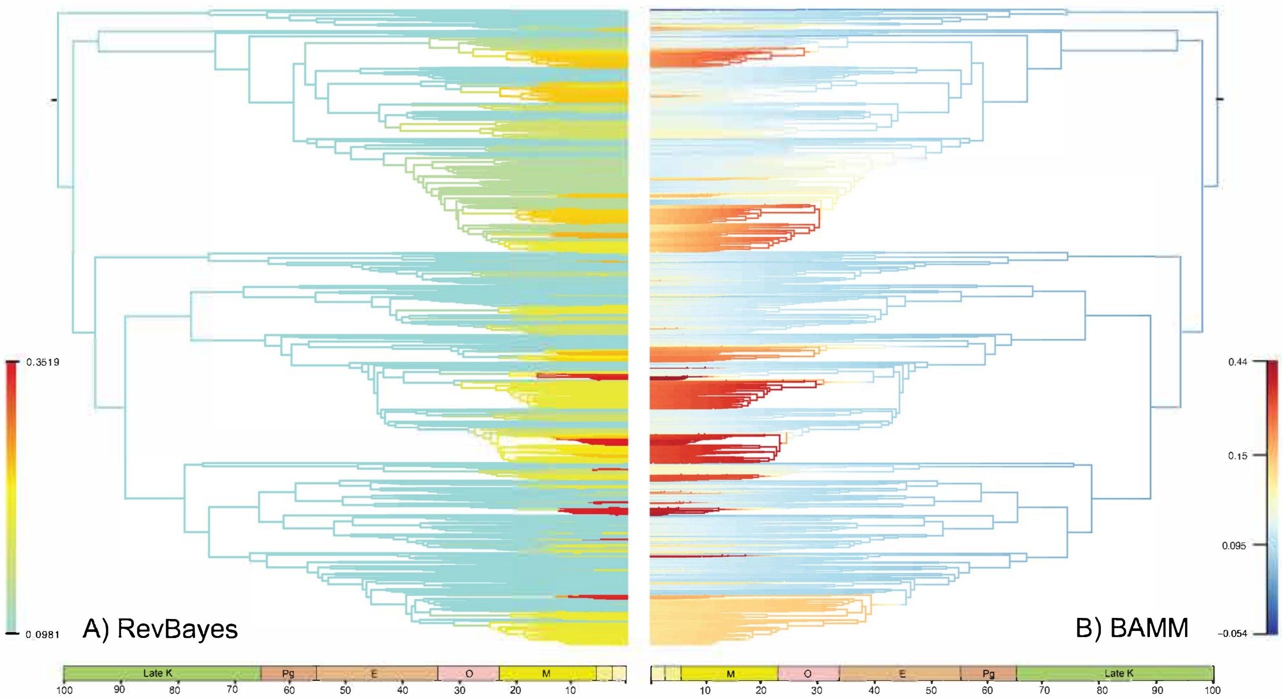

Figure S20. Diversification rate estimations from lineage-specific birth-death shift analyses in: A) RevBayes and B) BAMM.

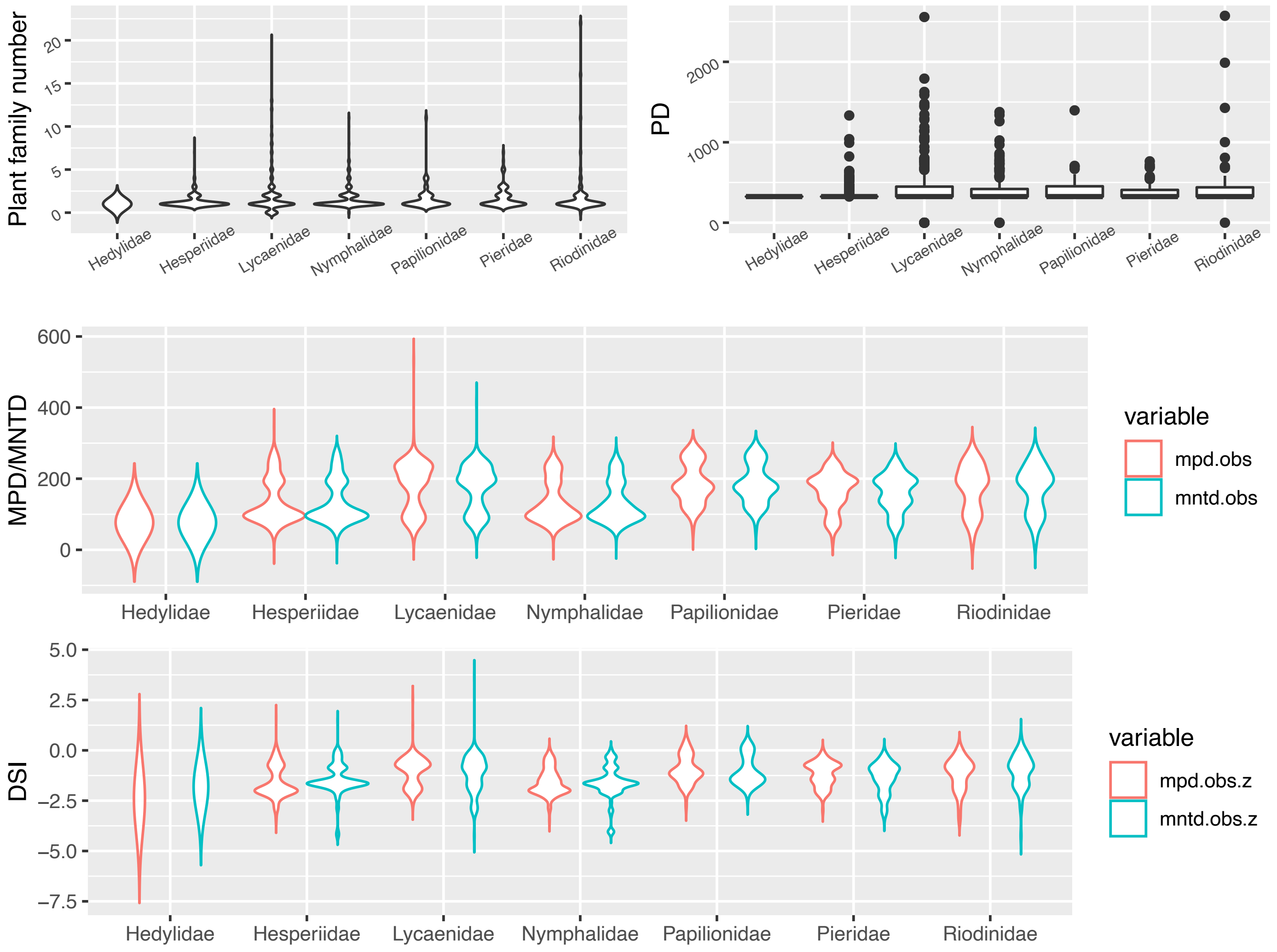

Figure S21. Hostplant richness and divergence across butterfly families. Distribution of hostplant richness measured by the **A)** number of hostplant families, **B)** phylogenetic distance (PD) of hostplant families, **C)** mean pairwise distance (MPD, red) and the mean nearest taxon distance (MNTD, blue), and **D)** distance-based speciation index (DSI) calculated from the normalized MPD (red) and MNTD (blue).

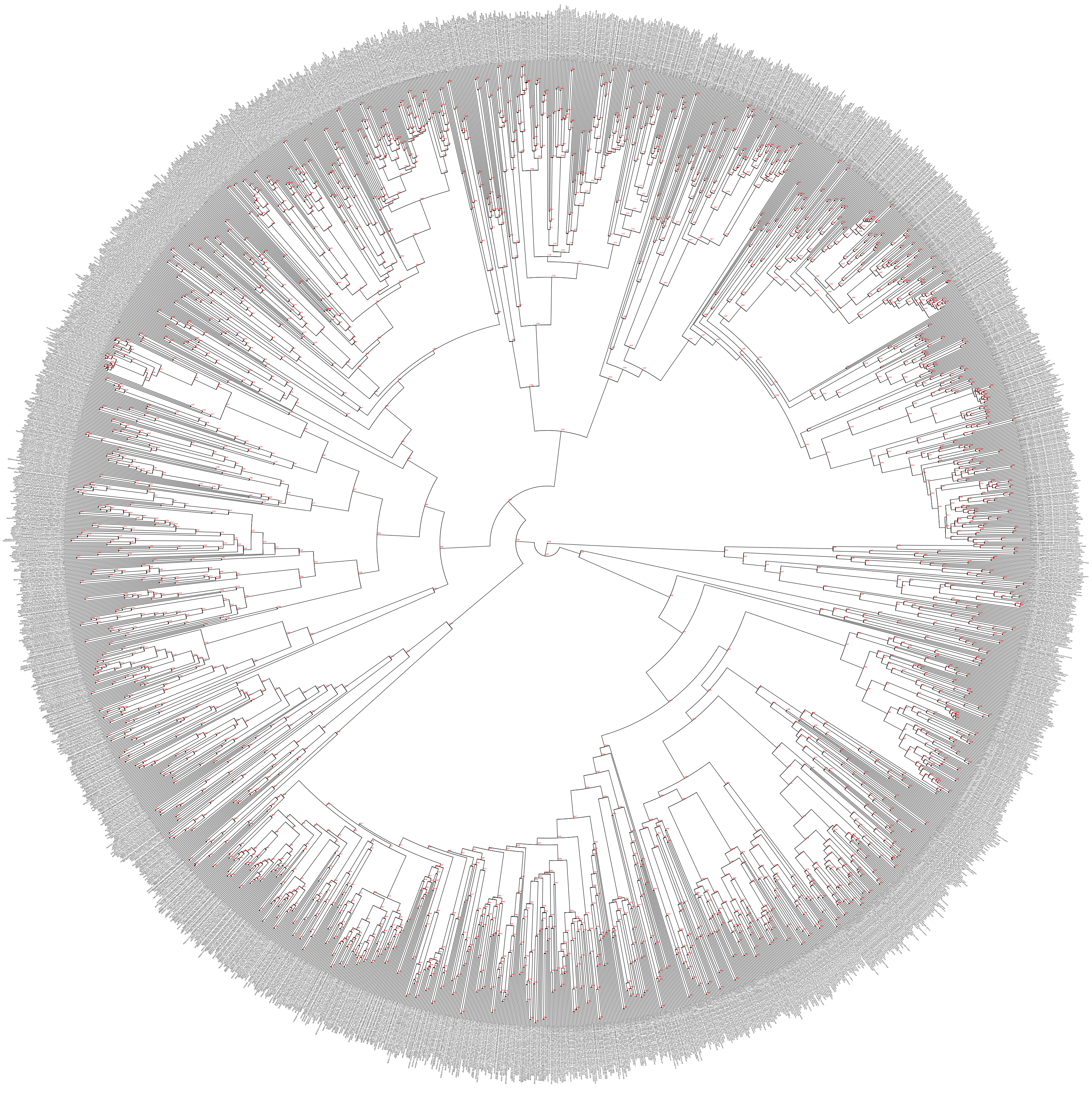

Figure S22. Dated amino acid (aa154) tree showing node labels at all splits. Tree excludes *Baronia brevicornis*.

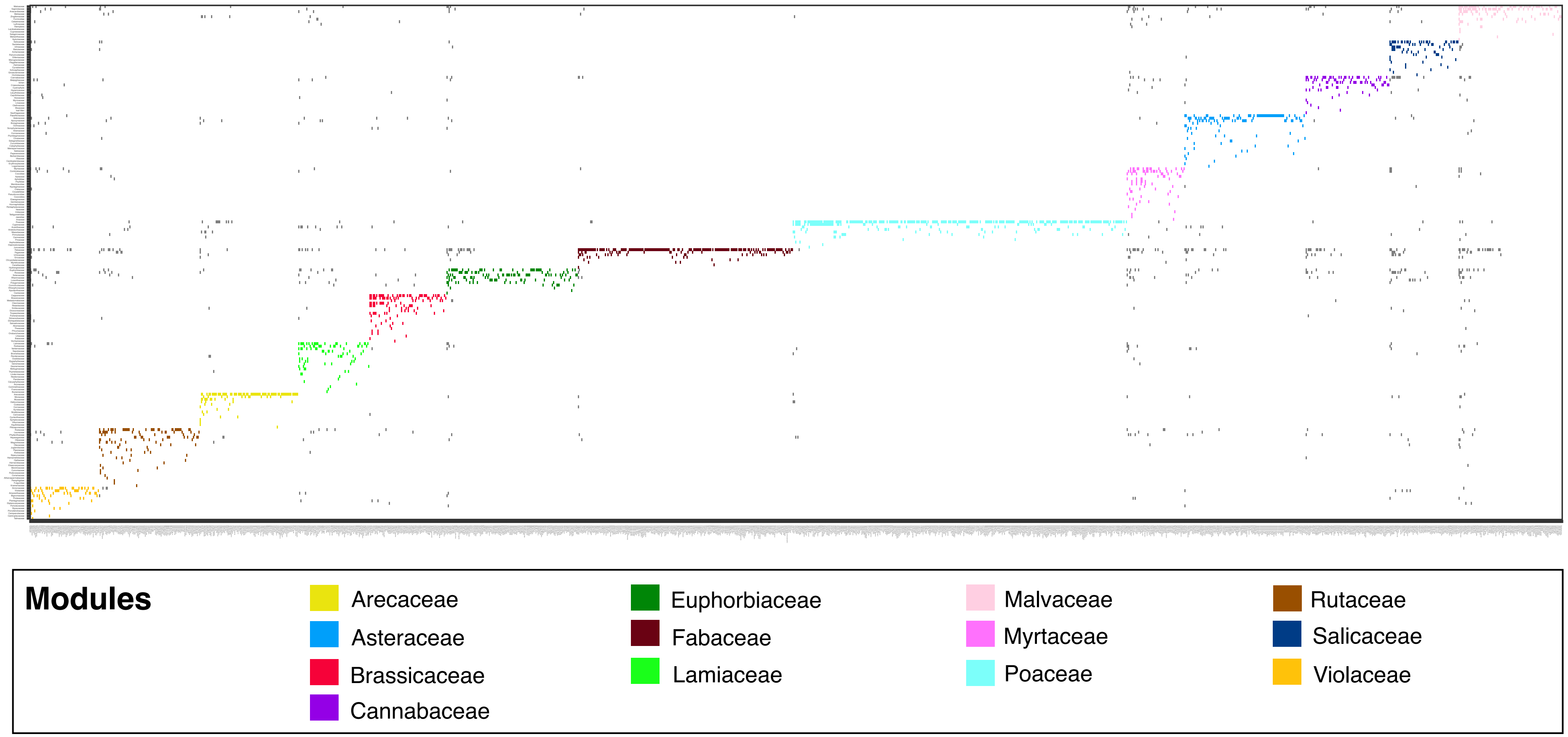

Figure S23. Network of interactions showing associations between butterfly species in our dataset (x-axis) and plants (y-axis). Squares indicate the presence of a butterfly-host interaction. Colors represent the 13 modules identified in the network, so that interactions between taxa assigned to the same module are colored by module, while grey squares show interactions between taxa assigned to different modules.

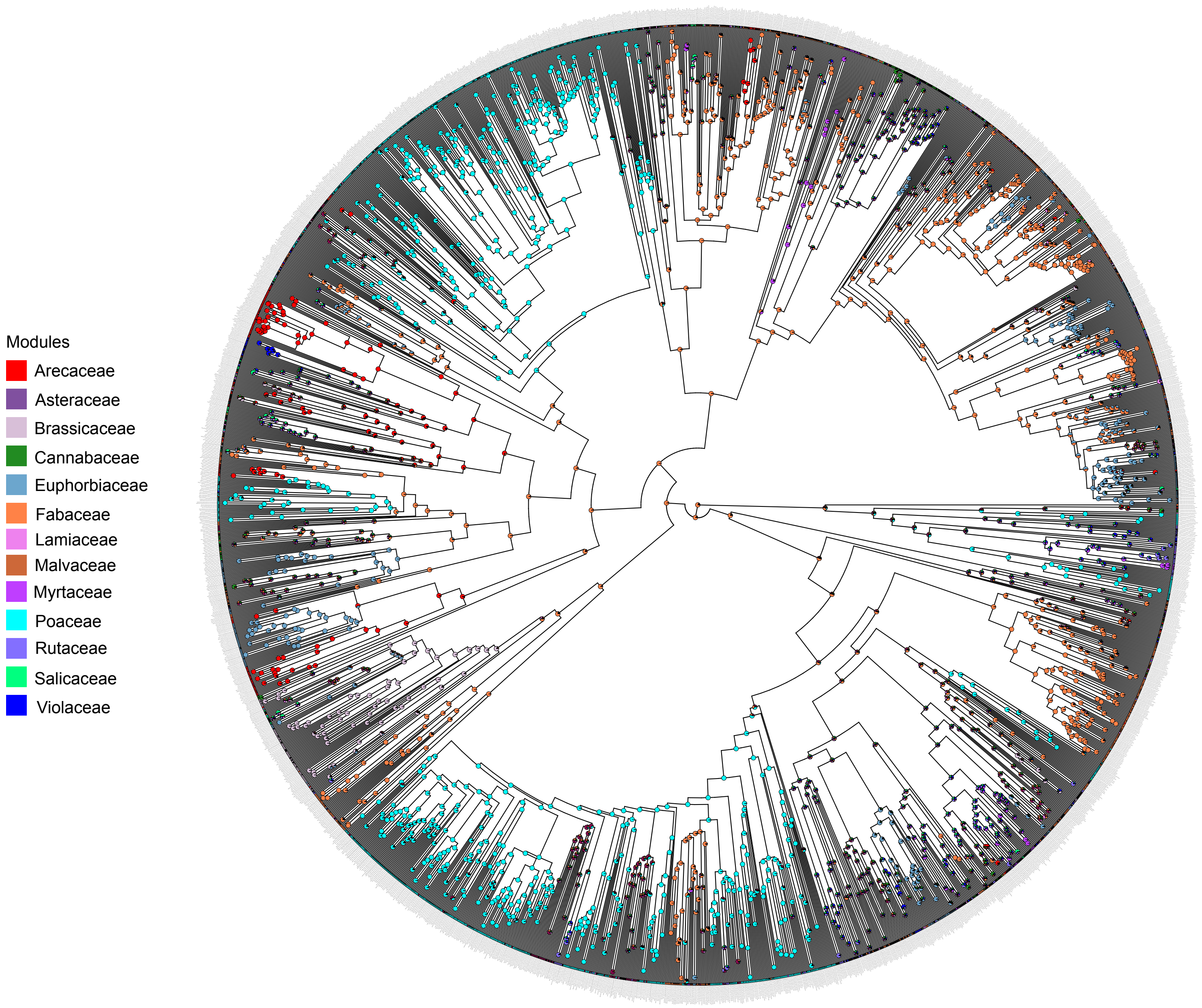

Figure S24. Ancestral state reconstruction of host feeding module, conducted in SIMMAP. Modules are named after the most dominant host group in the module. For a complete description of each module, see Table S33.

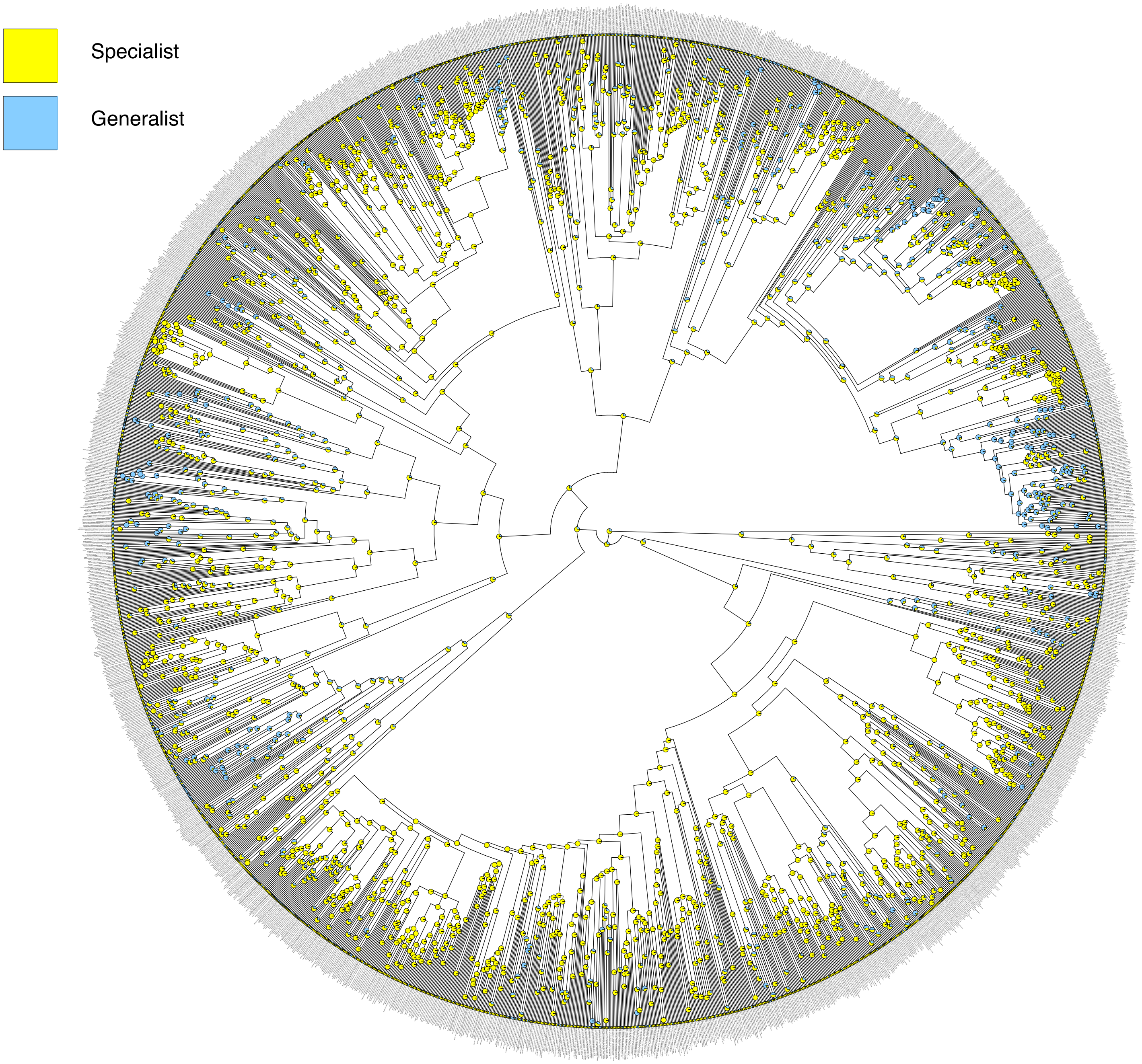

Figure S25. Evolution of specialist and generalist larval feeding behavior, as estimated in SIMMAP.

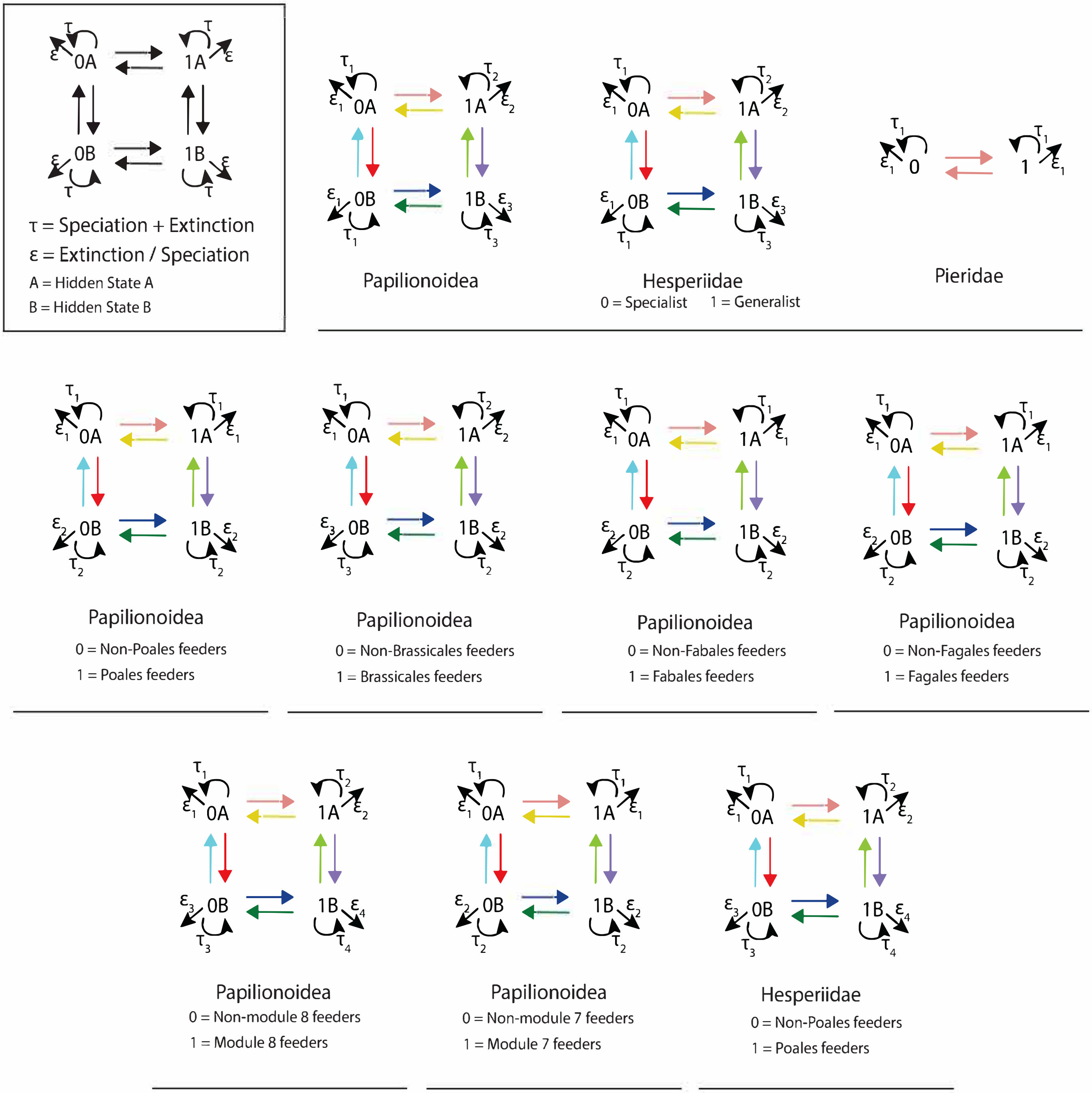

Figure S26. Best fit HiSSE models suggest no link between butterfly diversification rates and plants. The figure only shows results in which the ΔAIC between the first and second best model was > 2. Colors indicate different rates (observed and hidden states: 0/1; A/B).
