# Supplementary material for: A global phylogeny of butterflies reveals their evolutionary history, ancestral hosts and biogeographic origins
Source: Nat Ecol Evol. 2023 May 15;7(6):903–13. doi: 10.1038/s41559-023-02041-9 (PMC10250192; doi:10.1038/s41559-023-02041-9)
Supplement: Supplementary file 2 — Reporting Summary [file 41559_2023_2041_MOESM2_ESM.pdf]

nature portfolio

Corresponding author(s): Akito Y. Kawahara
Naomi E. Pierce
David J. Lohman

Last updated by author(s): Feb 2, 2023

Reporting Summary

Nature Portfolio wishes to improve the reproducibility of the work that we publish. This form provides structure for consistency and transparency in reporting. For further information on Nature Portfolio policies, see our Editorial Policies and the Editorial Policy Checklist.

Statistics

For all statistical analyses, confirm that the following items are present in the figure legend, table legend, main text, or Methods section.

n/a	Confirmed	
☒	☐	The exact sample size (*n*) for each experimental group/condition, given as a discrete number and unit of measurement
☒	☐	A statement on whether measurements were taken from distinct samples or whether the same sample was measured repeatedly
☒	☐	The statistical test(s) used AND whether they are one- or two-sided<br>*Only common tests should be described solely by name; describe more complex techniques in the Methods section.*
☒	☐	A description of all covariates tested
☒	☐	A description of any assumptions or corrections, such as tests of normality and adjustment for multiple comparisons
☒	☐	A full description of the statistical parameters including central tendency (e.g. means) or other basic estimates (e.g. regression coefficient) AND variation (e.g. standard deviation) or associated estimates of uncertainty (e.g. confidence intervals)
☒	☐	For null hypothesis testing, the test statistic (e.g. *F*, *t*, *r*) with confidence intervals, effect sizes, degrees of freedom and *P* value noted<br>*Give P values as exact values whenever suitable.*
☒	☐	For Bayesian analysis, information on the choice of priors and Markov chain Monte Carlo settings
☒	☐	For hierarchical and complex designs, identification of the appropriate level for tests and full reporting of outcomes
☒	☐	Estimates of effect sizes (e.g. Cohen's *d*, Pearson's *r*), indicating how they were calculated

Our web collection on statistics for biologists contains articles on many of the points above.

Software and code

Policy information about availability of computer code

Data collection	No software was used
Data analysis	The following programs and code were used for data analysis: R, TrimGalore!, MAFFT, FASconCAT, Iq-TREE, published scripts from Breinholt et al. (2018), SPAdes, BLAST+, FastTree, PhyloTreePruner, Trimal, MACSE, PartitionFinder2, FastTree, catfasta2phyml, MonoPhy, Degen, ASTRAL, TreePL, BAMM, BAMMtools, RevBayes, picante, bipartite, Phytools, hisse, BioGeoBEARS, DECX.<br>A new R script for running analyses in picante was created by co-authors of this manuscript and is publicly available on GitHub (https://github.com/lmcai/BNet-PD-analysis)<br>A new R script for partially automating TreePL analyses was created by co-authors of this manuscript and is publicly available on GitHub (https://github.com/sunray1/treepl)<br><br>References for all existing programs, and github links for all novel code, have been provided in the main text and/or the Extended Online Methods.

For manuscripts utilizing custom algorithms or software that are central to the research but not yet described in published literature, software must be made available to editors and reviewers. We strongly encourage code deposition in a community repository (e.g. GitHub). See the Nature Portfolio guidelines for submitting code & software for further information.

Data

Policy information about availability of data

All manuscripts must include a data availability statement. This statement should provide the following information, where applicable:

- Accession codes, unique identifiers, or web links for publicly available datasets
- A description of any restrictions on data availability
- For clinical datasets or third party data, please ensure that the statement adheres to our policy

All supplementary figures, tables, and data archives are available on Figshare (https://doi.org/10.6084/m9.figshare.21774899). Genomic data for all newly sequenced specimens in this study have been uploaded to GenBank as part of BioProject PRJNA714105. Individual BioSample accession numbers for each specimen are provided in Table S1.

Human research participants

Policy information about studies involving human research participants and Sex and Gender in Research.

Reporting on sex and gender	this information has not been collected
Population characteristics	see above
Recruitment	see above
Ethics oversight	see above

Note that full information on the approval of the study protocol must also be provided in the manuscript.

Field-specific reporting

Please select the one below that is the best fit for your research. If you are not sure, read the appropriate sections before making your selection.

☐ Life sciences ☐ Behavioural & social sciences ☒ Ecological, evolutionary & environmental sciences

For a reference copy of the document with all sections, see nature.com/documents/nr-reporting-summary-flat.pdf

Ecological, evolutionary & environmental sciences study design

All studies must disclose on these points even when the disclosure is negative.

Study description	We sequenced 391 genes from nearly 2,300 butterfly species to reconstruct a new, phylogenomic tree of butterflies
Research sample	A total of 2,248 butterfly specimens representing 2,244 species in 1,644 genera was included for the molecular component of this study, along with 10 outgroups from other lepidopteran superfamilies. We aimed to include at least one species from every valid genus and sequenced the type species of each genus whenever possible.
Sampling strategy	Specimens were collected in 90 countries over a 70-year period by over 300 people, preserved and deposited in one of the 28 specimen collections from which we obtained new tissue samples.<br>We also obtained sequence data from 343 published genomes and transcriptomes.
Data collection	We extracted DNA from 1,915 specimens that were 1) stored in ethanol and frozen; 2) dried and stored in glassine envelopes under ambient conditions (papered); or 3) dried, spread and pinned in a museum collection. For ethanol-preserved and enveloped specimens, we prioritized tissue from the thorax, but if thoracic tissue was unavailable, we used legs or tissue from the proximal region of the abdomen. We removed abdomens and/or up to three legs from museum specimens. In all cases, when multiple specimens of the same species were available, the most-recently collected specimen was used for DNA extraction.<br>All tissues were extracted with the OmniPrep DNA Extraction Kit for High Quality Genomic DNA Extraction<br>All DNA extractions were stored in 1.5 mL microcentrifuge tubes at -20° C prior to sequencing. Most extractions were performed at the McGuire Center for Lepidoptera and Biodiversity, Florida Museum of Natural History, University of Florida; the Museum of Comparative Zoology, Harvard University; or the Biology Department, City College of New York, City University of New York. Chinese samples that could not be exported to the US were sequenced at Annoroad Gene Technology Co., Ltd, Beijing, China.
Timing and spatial scale	DNA extractions took place between 2015-2019
Data exclusions	Multiple tests were conducted to determine whether sequences were contaminated. All samples that were included in the final dataset were checked first by BLAST, comparing its COI locus via the command-line blastn in Genbank. A minimum e-value of 0.0001 and maximum of five target sequences were specified to filter the search. The length of the sequence matching the query, its bit score, and taxonomy of resulting matched sequences were used to determine contamination. Samples with a low bit score were

flagged, and if the top three hits for that sequence was a species in a different butterfly family, the voucher specimen was examined for confirmation. Identification was confirmed by sharing specimen images with taxonomists, and in some cases, genitalia were dissected for verification.
We took three additional steps to identify contaminated or mislabeled sequences. (A) We generated gene trees in FastTree v2.1.744 for every locus. Along with visual inspection, branch tip lengths were compared in all gene trees using LongBranchFinder.pl50. If a particular gene tree branch was found to be significantly long (i.e., ≥ 8 SD from the mean branch length) the corresponding sequence for that taxon was removed from that locus (Table S31). (B) The R package MonoPhy v1.351 was used to perform a thorough search for any non-monophyletic groups present in an initial unpartitioned ML tree generated in IQ-TREE52, using our subfamily, tribe, and genus-level butterfly taxonomy. Questionable placements of taxa in the tree were verified by expert taxonomists and removed if there was a significant concern for contamination. (C) We calculated Quartet Fidelity (QF) scores53 and specimens that were deemed outliers were removed.

Reproducibility	Input files used in our analyses (as described in sections 9-20 of Extended Online Methods), have been provided in supplementary Data Archives and uploaded to Figshare, for the purposes of reproducibility.
Randomization	Random allocation of samples into groups is not necessary prior to a phylogenetic analysis. A random starting tree is automatically generated as part of the tree-searching algorithm in the program used for our primary phylogenetic analysis, IQ-tree.
Blinding	Blinding is not relevant to our study. The program used for our primary phylogenetic analysis, IQ-tree, is not provided any information on previous phylogenetic hypotheses, it interprets the dataset strictly based on the DNA sequences and partition information in the input files. No prior taxonomic knowledge was used when performing this analysis.

Did the study involve field work? ☒ Yes ☐ No

Field work, collection and transport

Field conditions	The majority of specimens that were used for the genetic portion of this study were obtained from legs and abdomens of museum specimens . These were collected before enforcement of the Nagoya Protocol on Access to Genetic Resources and the Fair and Equitable Sharing of Benefits Arising from their Utilization to the Convention on Biological Diversity (https://www.cbd.int/abs/). However, some specimens were obtained more recently through collecting efforts by our team and collaborators. Some countries, such as the USA, Chile, and many European countries, do not require permits for collecting non-protected species outside of protected areas, and some of our specimens were obtained in this manner. Other specimens were obtained in areas where permits were required. We list permits and their relevant countries and regions in Table S29; a pdf of permits can be found in Data S10.
Location	Locations where each specimen in our study was collected are provided in Table S1. As our dataset was comprised of over 2,000 specimens, there is not enough space to exhaustively list all locations here.
Access & import/export	We list permits and their relevant countries and regions in Table S29; a pdf of permits can be found in Data S10.
Disturbance	Butterfly specimens were collected individually with nets or by hand; this minimizes disturbance and prevents the death of other surrounding organisms that may otherwise happen with mass insect trapping techniques

Reporting for specific materials, systems and methods

We require information from authors about some types of materials, experimental systems and methods used in many studies. Here, indicate whether each material, system or method listed is relevant to your study. If you are not sure if a list item applies to your research, read the appropriate section before selecting a response.

Materials & experimental systems

n/a	Involved in the study	
☒	☐	Antibodies
☒	☐	Eukaryotic cell lines
☒	☐	Palaeontology and archaeology
☐	☒	Animals and other organisms
☒	☐	Clinical data
☒	☐	Dual use research of concern

Methods

n/a	Involved in the study	
☒	☐	ChIP-seq
☒	☐	Flow cytometry
☒	☐	MRI-based neuroimaging

Animals and other research organisms

Policy information about studies involving animals; ARRIVE guidelines recommended for reporting animal research, and Sex and Gender in Research

Laboratory animals	the study did not involve laboratory animals
Wild animals	All wild insects collected in the field were killed via a painless chemical killing agent (e.g., potassium cyanide) or via flash-freezing if such equipment was available at the field site. All insects were deceased prior to shipping.
Reporting on sex	this information has can be inferred via examination of the voucher specimens, but is not relevant to the study and not reported here. Sex is not relevant to a genus-level phylogenetic analysis.
Field-collected samples	all field-collected samples were deceased prior to being brought into a laboratory setting. No special parameters were necessary, beyond the basic sterilization and hygiene protocols used to minimize cross-contamination and ensure safety of people working in the lab.
Ethics oversight	no ethical approval was required for working with non-endangered invertebrate specimens.

Note that full information on the approval of the study protocol must also be provided in the manuscript.
